# ﻿Beyond nutmeg, mace, and cloves: Checklist of the liverworts and hornworts of Maluku Islands (Moluccas), Indonesia

**DOI:** 10.3897/phytokeys.239.116679

**Published:** 2024-03-19

**Authors:** Ainun Nadhifah, Ida Haerida, Fandri Sofiana Fastanti, Lars Söderström, Anders Hagborg, Matt von Konrat

**Affiliations:** 1 Department of Ecology and Evolutionary Biology, University of Tennessee, Knoxville, Tennessee, USA University of Tennessee Knoxville United States of America; 2 Research Center for Biosystematics and Evolution, National Research and Innovation Agency (BRIN), West Java, Indonesia National Research and Innovation Agency West Java Indonesia; 3 Norwegian University of Science and Technology, N-7491, Trondheim, Norway Norwegian University of Science and Technology Trondheim Norway; 4 Gantz Family Collections Center, Science & Education, Field Museum, 400 South Lake Shore Drive, Chicago, IL 60605– 2496, USA Gantz Family Collections Center, Science & Education, Field Museum Chicago United States of America

**Keywords:** Checklist, hornworts, Indonesia, liverworts, Maluku, Moluccas

## Abstract

The first ever liverwort and hornwort checklist is provided for the Maluku Islands (Moluccas/Spice Islands) of Indonesia. We report 355 accepted and 16 doubtful species and reject 22 species previously reported for Maluku Islands. The list is based on the specimens housed in the Herbarium Bogoriense (BO) and reports from over 500 literature references, including monographs, regional studies, and molecular investigations. The Maluku Islands are part of the Wallacea Biodiversity Hotspot with many unique species found only in Wallacea. Publications focusing on liverworts and hornworts of Maluku Islands are few and scattered. Considering regionally widespread species that have been recorded elsewhere, we predict that further fieldwork exploring the diversity of habitats coupled with collections unveiled from regional herbaria, a number of new records remain to be reported.

## ﻿Introduction

The Maluku Islands (Moluccas) is an island archipelago of over 1,000 islands situated in eastern Indonesia south of the Philippine island of Mindanao and east of Sulawesi, scattered in a series of irregular arcs spanning the Maluku, the Banda, and the Seram Seas (Zerner 1994; Leunufna and Evans 2014). The Maluku Islands were also formerly known as the Spice Islands because the archipelago held great geopolitical significance due to the abundance in spices as a commodity (Van Gils and Cox 1994). For instance, the spice nutmeg (*Myristicafragrans* Houtt.) a small-holder’s crop, with 3500 years of antiquity, is indigenous to the Banda Islands, and is now grown in about 12 countries including some secondary centers of domestication (Peter 2001; Sasikumar 2021).

**Figure 1.**
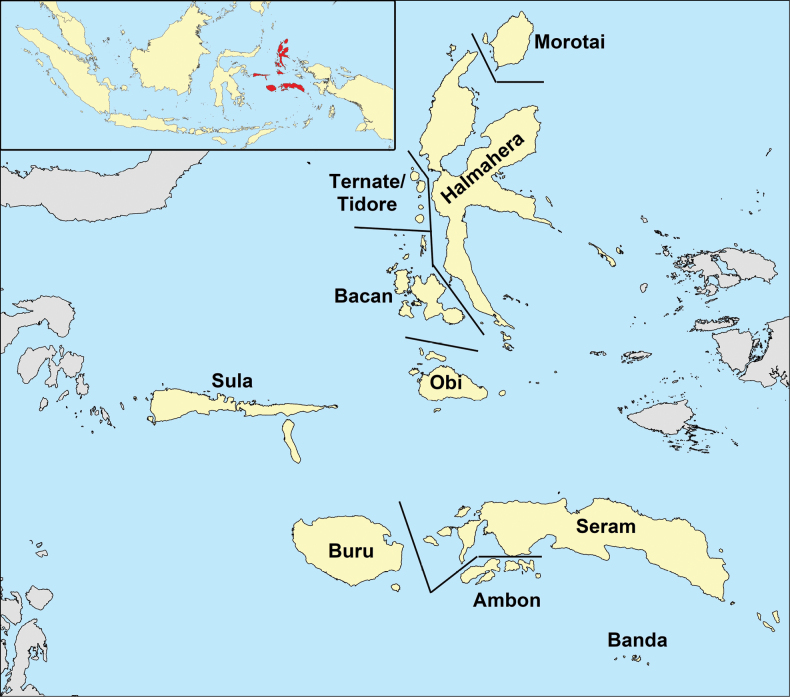
Moluccas in the Indonesian archipelago (inset) as defined in the present paper and the islands and archipelagos here used.

The biogeographical region of Maluku Islands (Fig. 1) encompasses the Indonesian geopolitical provinces of North Moluccas (Maluku Utara Province) and part of South Moluccas (Maluku Province) (Rutgrink et al. 2018). The major island groups in the north Moluccas are Halmahera, Bacan, Morotai, Obi, Sula, Ternate, and Tidore whereas Seram (previously known as Ceram), Buru, Banda, and Ambon are situated in the south. Many of the islands of Maluku are comprised of fragments of continental crusts (Monk et al. 1997) and are one of the most complex biogeographical and oceanographic areas on Earth (Pitriana et al. 2020). The island groups have experienced considerable, but very different tectonic displacements. During the last 10 million years, the North Moluccas moved westwards along the north coast of New Guinea to their current position. The Southern Moluccas moved northwards from Australia, west of New Guinea (Rutgrink et al. 2018). Not surprisingly the flora shows a marked similarity to those of New Guinea and Australia but also includes many endemic species and at least two endemic genera (*Parakibara* Philipson and *Siphokentia* Burret) (Burret 1927; Philipson 1985).

Famously, western exploration of the natural history of the Maluku Islands dates back to the 17^th^ century, starting with Georg Everhard Rumphius, and later, for example, Alfred Russel Wallace (Strack 1993; Lamoureux 1990). Notably, Wallace (1863), proposed a sharp boundary of the Indo-Australian Archipelago based upon land-mammal and land-bird distributions. It has since achieved iconic status and today its significance is recognized well beyond the confines of the biogeography community (Ali and Heaney 2021). Yet, it is noteworthy that the application in recent years of modern analytical techniques has not led to a consensus view on where the lines/areas should run/be placed (Ali and Heaney 2021). The Maluku Islands are part of what is termed as the Wallacea Biodiversity Hotspot, a region that covers an area of 338 thousand square kilometers with thousands of islands supporting highly diverse biological communities with many unique species found only in Wallacea (van Welzen et al. 2005; CEPF 2014). Wallacea was identified as one of the original 25 biodiversity hotspots in the world (Myers et al. 2000).

**Figure 2.**
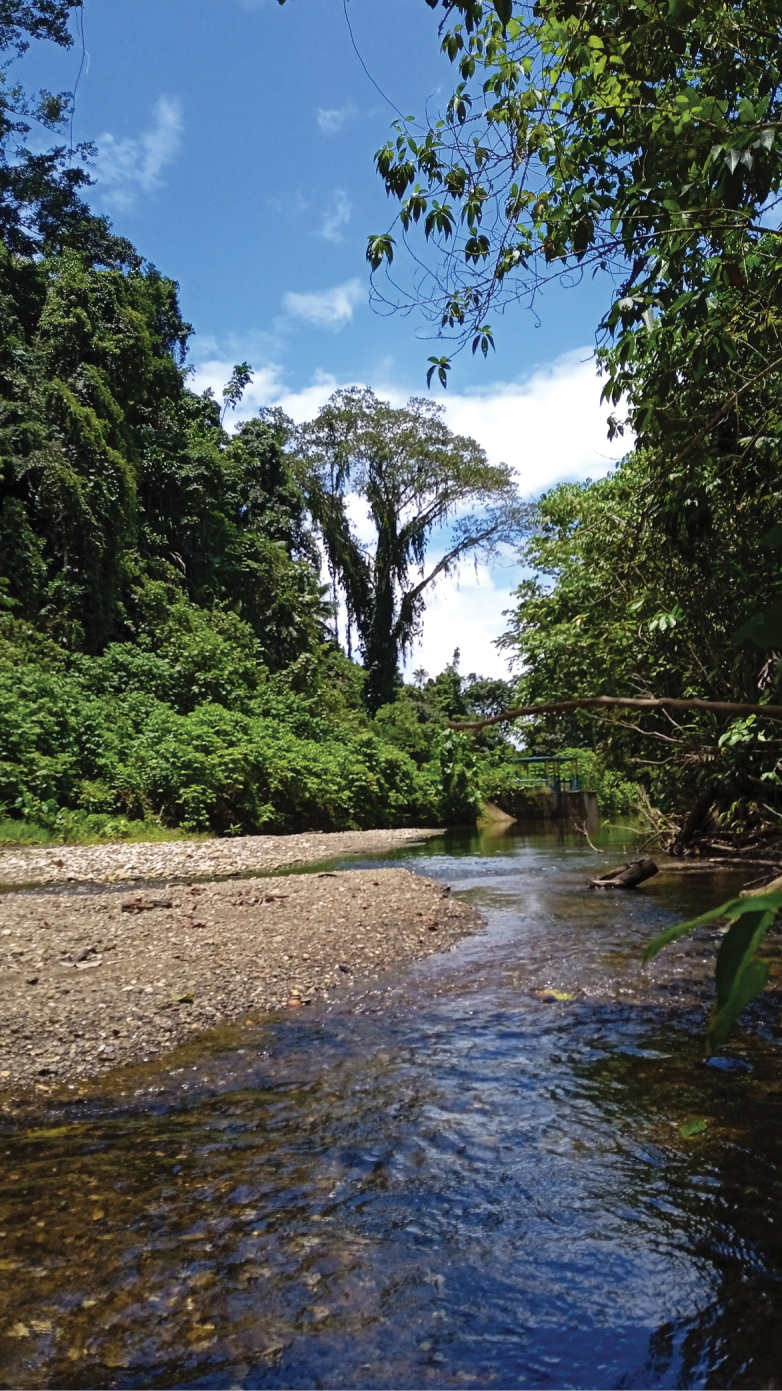
Vegetation of the Aketajawe-Lolobata National Park in Halmahera. Photo: Ida Haerida.

**Figure 3.**
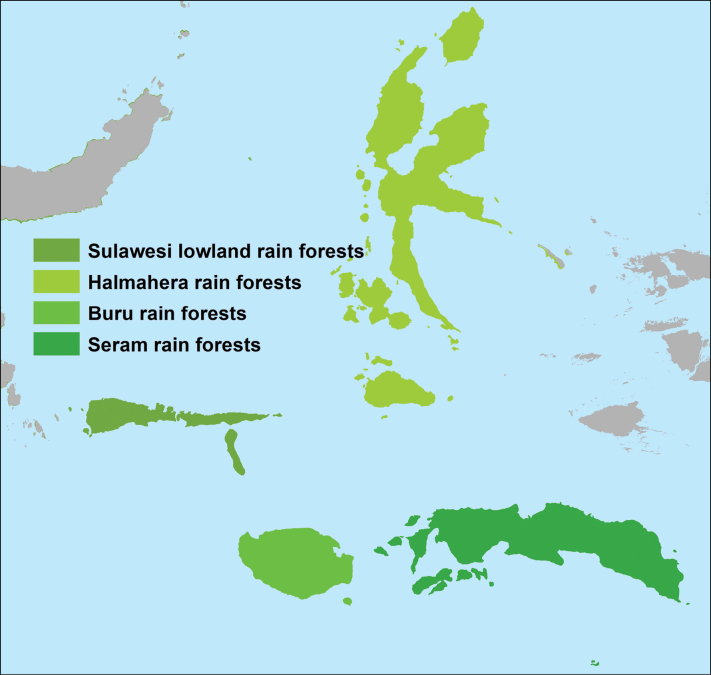
Ecoregions of the Moluccas Islands.

Maluku Islands are situated in the “Tropical and Subtropical Moist Broadleaved Forests” biome (Olson et al. 2001) and contribute approximately 24% of forest cover of the Wallacea land surface. The archipelago as here defined is separated into four ecoregions, eastern part of “Sulawesi Lowland Rainforests”, “Buru Rainforests”, “Halmahera Rainforests” and “Seram Rainforests” (Fig. 3, https://www.oneearth.org/bioregions/sulawesi-maluku-islands-au14/) and is considered home to much endemic flora (CEPF 2014). The Maluku Islands are one of the few places in the Indonesian archipelago where it is possible to find a complete altitudinal sequence of vegetation, with few places elsewhere in the tropics providing a comparable range (Edwards et al. 1993). Many of the forests in Maluku are lowland evergreen and semi-evergreen forests (Fig. 2), especially on Halmahera and adjacent islands, tropical montane forests, limestone (karst) geologies, and ultrabasic (serpentine) geologies (Ellen 1997; Abdo 2017).

Liverworts and hornworts are, together with mosses, commonly referred to as bryophytes. They represent three early diverging clades of land plants forming the second largest group of green land plants after flowering plants and are of critical biological, ecological, and phylogenetic significance. Bryophytes serve as the “macrophytes,” providing a matrix where many microscopic organisms live, including tardigrades, mites, rotifers, micro-molluscs, microalgae, microfungi, and prokaryotes (Gerson 1982; Huttunen et al. 2017; Muggia and Grube 2018). They also play an important role as possible indicators of climate change (e.g., Lindo et al. 2013; Ruklani et al. 2021), in nutrient cycling (Rieley et al. 1979) and through their water retention reducing soil nutrient loss and flooding risk (Anderson et al. 2010). Because of their physiological properties they have therefore been used extensively as ecological indicators (e.g., Pakeman et al. 2019; Déleg et al. 2021). Resolving relationships among bryophytes and their relationships to the remaining land plants is critical for understanding the evolution of fundamental innovations within land plants (Leebens-Mack et al. 2019).

Historically, relatively few publications have focused on plant collections from the Maluku Islands, despite the significance of the flora. Approximately 4,442 species of plants and fungi were recorded from Maluku Islands until 2017 (Retnowati and Rugayah 2019). Herbarium Amboinense, a classic work by Rumphius (1741–1755), contains descriptions of plants commonly found in Ambon and the surrounding islands. However, the floristic knowledge of liverworts and hornworts remains poor, especially compared to seed plants, and has scarcely been published. Seram and Ambon have been the most investigated areas for liverworts and hornworts and have been reported by several authors (e.g. Akiyama 2009; Doei 1987a, 1987b; Hasegawa 1986; Hattori 1986b; Inoue 1986; Mizutani 1986a). One of the most significant contributions to bryophytes of the Maluku Islands is the checklist of bryophytes from Seram and Ambon by Akiyama (2009) who recorded as many as 226 species of liverworts. One species, *Lepidoziaintegrifolia* Doei, is an endemic species of Seram (Doei 1987a).

We here present the first ever checklist of liverworts and hornworts for Maluku Islands in an effort to further promote bryological research, especially in the eastern part of Indonesia. The checklist complements recently published checklists for Indonesia including Söderström et al. (2010) for Java and Nadhifah et al. (2021) for the Lesser Sunda Islands. The significance of checklists is summarized by Söderström et al. (2008), including outlining the utility of checklists as powerful and important tools, and their applicability to taxonomy, systematics, and conservation.

## ﻿Liverwort types from Maluku

Not less than 113 names (9 of them invalid) have original material from the Maluku Islands. Of the validly published names, 13 have holotypes and 47 of the rest are lectotypified. However, 43 names remain to be typified. Here we designate lectotypes, or validate earlier unsuccessful lectotypifications, for 13 names. For a discussion on lectotypification procedures, including the concept of inadvertent lectotypifications, see Söderström and Hentschel (2023). In the list below we cite the lectotypification source and indicate where further typifications are needed.

***Chandonanthusgracilis* Herzog, *Hedwigia* 66 (6): 341, 1926 (Herzog 1926b).**

**Original citation.** Ceram: Hoale-Paß, ca. 1600 m, leg. E. Stresemann, 1911.

**Lectotype** (here designated, cf. Váňa et al. 2013: 30 as “holotype”). Indonesia, Moluccas, Seram, Hoale Pass, 1600 m., 1911, *Stresemann s.n.* (JE04005601, https://je.jacq.org/JE04005601).

**Note.** Only one specimen has been located but it cannot be ruled out that there exist duplicates elsewhere. Váňa et al. (2013: 30) call it “holotype” but this is too late for an inadvertent lectotypification. The specimen is also annotated as holotype by K. Amamoto in 2020. In case duplicates are found, we here formally designate it as a lectotype.

**Currently accepted name.***Plicanthushirtellus* (F.Weber) R.M.Schust. (see Váňa et al. 2013).

***Lepidozialongifolia* Steph., *Sp. Hepat. (Stephani)* 3: 606, 1909 (Stephani 1909a).**

**Original citation.** Hab. *Amboina* (Karsten).

**Lectotype** (here designated). Indonesia, Moluccas, Ambon, *Karsten* (G00069659 https://www.ville-ge.ch/musinfo/bd/cjb/chg/adetail.php?id=123550&lang=en).

**Note.** The lectotype is annotated “type” by Mizutani in Feb. 1975.

***Lophocoleadeningeri* Herzog, *Beih. Bot. Centralbl.* 38 (2): 321, 1921 (Herzog 1921).**

**Original citation.** Buru: Im Urwald des Kapalamadang, leg. Dr. K. Deninger †, 1906.

**Lectotype** (here designated). Indonesia, Moluccas, Buru, Im Urwald des Kapalamadang, *Deninger* 1906 (JE04005314 https://je.jacq.org/JE04005314).

**Note.** JE04005314 is marked as “holotype” by R. Grolle. We are not aware of any other type material.

**Lopholejeuneasagranavar.dentistipula Schiffn. ex P.Syd., *Just’s Bot. Jahresber.* 19 (1): 246, 1894 (Sydow 1894) (‘ *sagraeana* ’).**

**Original citation.** Ins. Amboina, in iisdem locis ac typica, pauca tantum frustula sed c. per. et ♂ inventa sunt (7. 6. 75).

**Lectotype** (here designated, Zhu and Gradstein 2005: 85 as “holotype”). Indonesia, Moluccas, Ambon, 7 Jun 1875, *D. Naumann* (FH).

**Note.**Zhu and Gradstein (2005: 85) call it “holotype” but this is too late for an inadvertent lectotypification.

**Currently accepted name.***Caudalejeuneareniloba* (Gottsche) Steph. (see Zhu and Gradstein 2005).

***Madothecacrenilobula* Herzog, *Beih. Bot. Centralbl.* 38 (2): 328, 1921 (Herzog 1921).**

**Original citation.** Buru: Im Urwald des Kapalamadang, leg. Dr. K. Deninger †, 1906.

**Lectotype** (here designated). Indonesia, Moluccas, Buru, Im Urwald des Kapalamadang, 1906, *K. Deninger* (JE04001514 https://je.jacq.org/JE04001514, isotype JE04001515 https://je.jacq.org/JE04001515).

**Note.** The lectotype was annotated “Type!” and the isotype “isotype” by S. Hattori in 1968 but has apparently never been published.

**Currently accepted name.***Porellajavanica* (Gottsche) Inoue (see Hattori 1975e).

***Mastigobryumdeningeri* Herzog, *Beih. Bot. Centralbl.* 38 (2): 322, 1921 (Herzog 1921).**

**Original citation.** Buru: In den Bergen Mittelburus, ca. 1500 m, leg. Dr. K. Deninger †, 1911.

**Lectotype** (here designated). Indonesia, Moluccas, Buru, Mittelburu, ca. 1500 m, 1911, *Deninger 10* (JE04006158 https://je.jacq.org/JE04006158, isolectotype JE04006157 https://je.jacq.org/JE04006157).

**Note.** The selected lectotype is richer than the isolectotype and has a label in Herzog’s handwriting marked “n.sp.”. The isolectotype is marked “Typus” and annotated as type and syn. to *Bazzanialongicaulis* by Kitagawa 18 Nov 1978 but no typification seems to have been published.

**Currently accepted name.***Bazzanialongicaulis* (Sande Lac.) Schiffn., syn. nov.>

***Mastigobryumnigricans* Herzog, *Beih. Bot. Centralbl.* 38 (2): 322, 1921 (Herzog 1921).**

**Original citation.** Buru: Im Urwald des Kapalamadang, leg. Dr. K. Deninger †, 1906.

**Lectotype** (here designated). Indonesia, Moluccas, Buru, Kapalamadang, 1906, *K. Deninger 112* (JE04006129 https://je.jacq.org/JE04006129).

**Note.** The lectotype is marked “n.sp.” on the label and annotated by N. Kitagawa 1991 as type.

***Mastigobryumstresemannii* Herzog, *Beih. Bot. Centralbl.* 38 (2): 324, 1921 (Herzog 1921).**

**Original citation.** Ceram: Paßhöhe zwischen Mansela und Wolu (Useahánpaß), ca. 1750 m, leg. E. Stresemann, 1911.

**Lectotype** (here designated). Indonesia, Moluccas, Seram, between Mansela and Wolu ca. 1750 m, 1911, *E. Stresemann 110*, (JE04005944 https://je.jacq.org/JE04005944).

**Note.** The lectotype is marked “n.sp.” on the label by Herzog and annotated as type by N. Kitagawa in 1978.

**Currently accepted name.***Bazzaniastresemannii* (Herzog) N.Kitag.

***Mastigobryumvermiculare* Herzog, *Hedwigia* 66 (6): 339, 1926 (Herzog 1926b).**

**Original citation.** Ceram: Hoale, ca.1100m, leg. E. Stresemann, 1911.

**Lectotype** (here designated). Indonesia, Moluccas, Seram, Hoale, ca. 1100 m, 1911, *Stresemann* (JE04006130 https://je.jacq.org/JE04006130).

**Note.** Only one specimen is online in JE and without any annotation.

***Pycnolejeuneaventricosa* Schiffn. ex P.Syd., *Just’s Bot. Jahresber.* 19 (1): 246, 1894 (Sydow 1894).**

**Original citation.** In insula Amboina cum aliis Lejeuniis parcissime, cum fl. ♀ et periantbiis vetustis (7. 6. 75) [cf. Schiffner 1890: 32].

**Lectotype** (here designated, cf. Zhu and Lai 2005: 200 as “holotype”). Indonesia, Moluccas, Ambon, Gazellen Expedition, 7.6.1875, *Naumann* (FH00284228 https://kiki.huh.harvard.edu/databases/specimen_search.php?mode=details&id=206370).

**Note.** “Holotype” by Zhu and Lai (2005: 200) is too late to be an inadvertent lectotype (cf. ICN2018 Art. 7.11) and is here designated as lectotype.

**Currently accepted name.***Cheilolejeuneaventricosa* (Schiffn. ex P.Syd.) Xiao L.He

***Schistochilaamboinensis* Steph.**, ***Sp. Hepat. (Stephani)* 4: 77, 1909 (Stephani 1909c).**

**Original citation.** Hab. *Amboina*.

**Lectotype** (here designated, cf. So 2003a: 87 as “holotype”). Indonesia, Moluccas, Ambon, 1883, *Luerssen 1270* (G00067852 https://www.ville-ge.ch/musinfo/bd/cjb/chg/adetail.php?id=131418&lang=en).

**Note.**So (2003a: 87 as “holotype”) is too late for an inadvertent lectotypification.

**Currently accepted name.***Schistochilabeccariana* (De Not.) Trevis. (see So 2003a).

***Schistochilainversa* Herzog, *Hedwigia* 66 (6): 342, 1926 (Herzog 1926b).**

**Original citation.** Ceram: Hoale-Paß, ca. 1600 m, leg. E. Stresemann, 1911; in Bruchstücken aus *Lepidoziaceramensis* und *L. Ferdinandi Mülleri* herausgelesen.

**Lectotype** (here designated, cf. So 2003a: 98 as “holotype”). Indonesia, Moluccas, Seram, Hoale-Pass, ca. 1600 m, 1911, *E. Stresemann s.n.* (JE04002842 https://je.jacq.org/JE04002842).

**Note.**So (2003a: 98 as “holotype”) is too late to be an inadvertent lectotypification.

**Currently accepted name.***Schistochilasciurea* (Nees) Schiffn. (see So 2003a).

***Schistochilapurpurascens* Herzog, *Hedwigia* 66 (6): 341, 1926 (Herzog 1926b).**

**Original citation.** Ceram: Hoale-Paß, ca. 1600 m, leg. E. Stresemann, 1911.

**Lectotype** (here designated, cf. So 2003a: 80 as “holotype”). Indonesia, Moluccas, Seram, Hoale-Pass, ca. 1000 m, Mar-Aug 1911, *E. Stresemann 201* (JE04002840 https://je.jacq.org/JE04002840, isotype L-937.235.12 https://data.biodiversitydata.nl/naturalis/specimen/L%20%200444630).

**Note.** “holotype” by So (2003a: 80) is too late for an inadvertent lectotypification.

**Currently accepted name.***Schistochilaacuminata* Steph. (see So 2003a).

## ﻿Materials and methods

Nomenclature and taxonomy follow the world checklist of hornworts and liverworts (Söderström et al. 2016) with some updates from recent literature. Sources include more than 500 publications found through the work with Early Land Plants Today (ELPT) database of liverwort taxonomy and distribution, and with some consultation with taxonomic experts. In addition, we also examined the specimens collected from the Maluku Islands in the Herbarium Bogoriense (BO). The checklist follows a similar format of a series of previous liverwort and hornwort regional checklists by the authors, e.g., Java, Lesser Sunda Islands (Söderström et al. 2010; Nadhifah et al. 2021). References citing primary reports (citing specimens or material seen by the author) are given in bold. Secondary references not citing any material seen are given in normal font. In cases where we are not aware of any primary source, or the earliest report is a secondary source without any clear connection to a primary source published later, we comment on the situation. In the current checklist we note under which name a taxon is reported except when it is reported under the currently accepted name.

**Figure 4.**
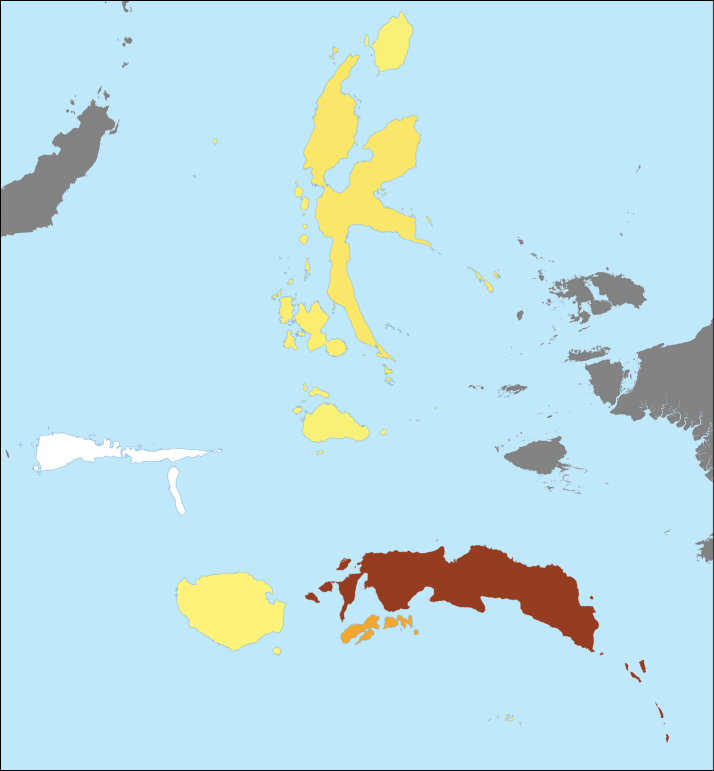
Species richness on the different islands/archipelagos of Moluccas. The darker the more species known (cf. Table 1). White (Sulu I) means that no liverwort or hornwort species are known from the area.

We here accept reports of 355 species from the area in addition to 16 reported species that we consider as doubtful and 21 species reported whose occurrence we reject from the islands. Species number known varies a lot between the islands/archipelagos (Table 1, Fig. 4). We are not aware of a single report from Sula while 258 species are known from Seram. Thus, we expect that with further investigations the number of species will rise considerably for many of the islands in the region.

**Table 1.** Number of accepted, doubtful and rejected species of liverworts and hornworts from Moluccas and the recognized areas (islands/archipelagos) within the Moluccas.

Area	Accepted	Doubtful	Rejected
Ambon	143	11	10
Bacan	26	0	0
Banda	7	2	0
Buru	13	0	2
Halmahera	31	3	0
Morotai	2	0	0
Obi	2	0	0
Seram	258	7	5
Sulu	0	0	0
Ternate/Tidore	22	0	2
Moluccas	355	16	22

## ﻿Taxa accepted for Moluccas

### ﻿Anthocerotophyta

***Dendroceros* Nees**

*Dendrocerosacutilobus* Steph. **MOLUCCAS**: Ambon: **LECTOTYPE** [Piippo 1993a: 44], **LECTOTYPE** of *Dendroceroskarstenii* [to be designated], **Schiffner 1898** as *Dendroceroskarstenii*, **Stephani 1909b, Stephani 1917** as *Dendroceroskarstenii*, Stephani 1917, **Bonner 1965** also as *Dendroceroskarstenii*, **Hasegawa 1980**, 1982, 1986, 2002, Piippo 1993a, **Peñalosa et al. 2019**. Seram: A**kiyama 1986, Hasegawa 1986**, 2002, **Akiyama 2009**.

*Dendroceroscavernosus* J.Haseg. **MOLUCCAS**: Seram: **Akiyama 1986, Hasegawa 1986**, 2002, **Akiyama 2009**.

*Dendroceroscrispus* (Sw.) Nees. **MOLUCCAS**: Amélio et al. 2021. Ambon: **Schiffner 1893b**.

*Dendrocerosfoliicola* J.Haseg. **MOLUCCAS**: Seram: **Akiyama 1986, Hasegawa 1986** as *Dendroceros* ‘*foliicala*’, **Akiyama 2009**.

*Dendrocerosjavanicus* (Nees) Nees. **MOLUCCAS**: Seram: **Akiyama 1986, 2009, Hasegawa 1986**.

*Dendrocerospedunculatus* Steph. **MOLUCCAS**: Ambon: **LECTOTYPE** [Hasegawa 1980: 306], **Stephani 1909b**, 1917, **Bonner 1965**, Hasegawa 1980, Piippo 1993a, **Peñalosa et al. 2019**.

*Dendrocerosseramensis* J.Haseg. **MOLUCCAS**: Seram: **HOLOTYPE, Akiyama 1986, 2009, Hasegawa 1986, Engel 1993, Crosby and Engel 2006, Peñalosa et al. 2019**.

***Folioceros* D.C.Bharadwaj**

*Foliocerosamboinensis* (Schiffn.) Piippo. **MOLUCCAS**: Ambon: **Schiffner 1890, 1898** both as *Anthocerosamboinensis*, **Sydow 1894** as *Anthocerosamboinensis*, Stephani 1916 as *Aspiromitusamboinensis*, Meijer 1954 as *Anthocerosamboinensis*, **Bonner 1962b** as *Aspiromitusamboinensis* and *Anthocerosamboinensis*, Hasegawa 1993 as *Anthocerosamboinensis*.

*Foliocerosappendiculatus* (Steph.) J.Haseg. **MOLUCCAS**: Seram: **Akiyama 1986, Hasegawa 1986**, 2002 as *Anthocerosappendiculatus*, **Akiyama 2009**.

*Foliocerosglandulosus* (Lehm. et Lindenb.) D.C.Bharadwaj. **MOLUCCAS**: Inoue and Miller 1965 as *Anthocerosglandulosus*, Inoue 1965 as *Anthocerosglandulosus*, Miller et al. 1983 as *Anthocerosglandulosus*, Bapna and Kachroo 2000a. Banda: **Meijer 1957** as *Anthocerosglandulosus*. ?Seram: Piippo 1993a. Note: we are not aware of any first hand report from Seram.

***Megaceros* Campb.**

*Megacerosflagellaris* (Mitt.) Steph. **MOLUCCAS**: Ambon: **Schiffner 1893b, 1898** both as *Anthocerosgrandis*, Merrill 1906 as *Anthocerosgrandis*, Miller et al. 1983 as *Megacerosgrandis*. Seram: **Akiyama 1986, 2009, Hasegawa 1986**.

***Phaeoceros* Prosk.**

*Phaeoceroscarolinianus* (Michx.) Prosk. **MOLUCCAS**: Seram: **Akiyama 1986, 2009** both as Phaeoceroslaevissubsp.carolinianus, **Hasegawa 1986** as Phaeoceroslaevissubsp.carolinianus.

***Phaeomegaceros* R.J.Duff, J.C.Villarreal, Cargill et Renzaglia**

*Phaeomegaceroshirticalyx* (Steph.) R.J.Duff, J.C.Villarreal, Cargill et Renzaglia. **MOLUCCAS**: Ambon: **Hasegawa 1986** as *Phaeocerospolyandrus*, Campbell and Hasegawa 1993 as *Phaeoceroshirticalyx*, **Akiyama 2009** as *Phaeocerospolyandrus*.

### ﻿Marchantiophyta

***Acrolejeunea* (Spruce) Steph.**

*Acrolejeuneafertilis* (Reinw., Blume et Nees) Schiffn. **MOLUCCAS**: **LECTOTYPE** of *Ptychocoleustener* [Gradstein 1975: 86], Bapna and Kachroo 2000b, Singh et al. 2010. Ambon: **Sande Lacoste 1864** as *Phragmicomafertilis*, **Schiffner 1898, Gradstein 1975**.

*Acrolejeuneapycnoclada* (Taylor) Schiffn. **MOLUCCAS**: Mizutani 1977, Bapna and Kachroo 2000b, Haerida et al. 2010, Siregar et al. 2014, Siregar 2015. Ambon: Verdoorn 1934a, **1934b** both as *Ptychocoleuspycnocladus*, Swanson and Miller 1969 as *Ptychocoleuspycnocladus*, Kamimura 1974 as *Ptychocoleuspycnocladus*, Miller et al. 1983 also as *Ptychocoleuspycnocladus*. Seram: **Mizutani 1986a, Akiyama 2009**.

— subsp. pycnoclada. **MOLUCCAS**: Ambon: **LECTOTYPE** of *Acrolejeunearostrata* [Gradstein 1975: 109], **LECTOTYPE** of *Ptychocoleusbrunneus* [Gradstein 1975: 109 as “holotype”], **ORIGINAL MATERIAL** of Acrolejeunearostratavar.major, **ORIGINAL MATERIAL** of Acrolejeunearostratavar.minor, **Schiffner 1890** as Acrolejeunearostrata and its var. major and var. minor, **Bonner 1962b** as *Acrolejeunearostrata* and its var. Major and var. *minor*, **Gradstein 1975**, Miller et al. 1983 as *Ptychocoleusbrunneus*, **Geissler and Bischler 1987** as *Lejeunearostrata* var. *Minor*, **Geissler and Bischler 1989** as *Ptychocoleusbrunneus*.

*Acrolejeuneasecurifolia* (Nees) Steph. **MOLUCCAS**: Seram: **Mizutani 1986a** as *Acrolejeuneasecurifolia*, **Akiyama 2009**.

— subsp. hartmannii (Steph.) Gradst. **MOLUCCAS**: Ambon: **Gradstein 1975**, Bonner 1977, Gradstein et al. 2002, Piippo et al. 2002.

***Acromastigum* A.Evans**

*Acromastigumaurescens* A.Evans. **MOLUCCAS**: Ambon: **Grolle 1978**.

*Acromastigumbancanum* (Sande Lac.) A.Evans. **MOLUCCAS**: Ambon: **Grolle 1978** as *Acromastigumbidenticulatum*.

*Acromastigumdivaricatum* (Nees) A.Evans ex Reimers. **MOLUCCAS**: Ambon: **Schiffner 1893b, 1898** both as *Bazzaniadivaricata*, **Evans 1934**, Brown and Renner 2014. Seram: **Akiyama 1986, 2009**.

*Acromastigumechinatiforme* (De Not.) A.Evans. **MOLUCCAS**: Ambon: **Schiffner 1893b, 1898** as *Bazzaniaechinatiformis*, **Stephani 1909a** as *Mastigobryumechinatiforme*, **Evans 1934**, Bonner 1962b, 1963a as *Bazzaniaechinatiformis*, Grolle 1965, 1978, Pócs 1971, Miller et al. 1983, Piippo 1991, Schuster 2000a. Seram: **Grolle 1978**, Miller et al. 1983, Piippo 1991.

*Acromastiguminaequilaterum* (Lehm. et Lindenb.) A.Evans. **MOLUCCAS**: Ambon: **Evans 1934**, Jovet-Ast 1958, Bonner 1962b, Sharma and Srivastava 1993, Schuster 2000a.

*Acromastigumlobuliferum* A.Evans. **MOLUCCAS**: Ambon: **Grolle 1978**.

***Acroscyphella* N.Kitag. et Grolle**

*Acroscyphellatjiwideiensis* (Sande Lac.) N.Kitag. et Grolle. **MOLUCCAS**: Piippo 1985 as *Clasmatocoleatjiwideiensis*. Ambon: **LECTOTYPE** of *Chiloscyphusfalcifolius* [Bonner 1963b: 733], **Stephani 1907c** as *Chiloscyphusfalcifolius*, **Bonner 1963b** as *Chiloscyphusfalcifolius*, **Grolle 1984** as *Clasmatocoleatjiwideiensis*, **1986**, Enroth 1991 as *Clasmatocoleatjiwideiensis*.

***Allorgella* Tixier**

*Allorgellasemperiana* (Steph.) Bechteler, G.E.Lee, Schäf.-Verw. et Heinrichs. **MOLUCCAS**: Seram: **Mizutani 1986a** as *Otolejeuneasemperiana*, **Akiyama 2009** as *Otolejeuneasemperiana*.

*Allorgellazantenii* (Grolle) Bechteler, G.E.Lee, Schäf.-Verw. et Heinrichs. **MOLUCCAS**: Seram: **Bi et al. 2019**.

***Anastrophyllopsis* (R.M.Schust.) Váňa et L.Söderstr.**

*Anastrophyllopsisrevoluta* (Steph.) Váňa et L.Söderstr. **MOLUCCAS**: Ah-Peng et al. 2010 as *Anastrophyllumrevolutum*. Seram: **Akiyama 1986** as *Anastrophyllumrevolutum*, Váňa and Piippo 1989b as *Anastrophyllumrevolutum*, Váňa 1991b as *Anastrophyllumrevolutum*, **Akiyama 2009** as *Anastrophyllumrevolutum*.

***Anastrophyllum* (Spruce) Steph.**

*Anastrophyllumpiligerum* (Nees) Steph. **MOLUCCAS**: Váňa and Engel 2013. Halmahera: **Váňa and Piippo 1989b**, Váňa 1991b.

***Aneura* Dumort.**

*Aneuraamboinensis* Steph. **MOLUCCAS**: Ambon: **LECTOTYPE** [Bonner 1962b: 86], **Stephani 1899b, Bonner 1962b**.

*Aneurapinguis* (L.) Dumort. **MOLUCCAS**: Ambon: **Schiffner 1893b** as Aneurapinguisf.normalis, **1898** as *Riccardiapinguis*.

***Balantiopsis* Mitt.**

BalantiopsisciliarisS.Hatt.subsp.novoguineensis S.Hatt. **MOLUCCAS**: Seram: **Akiyama 1986, 2009**.

***Bazzania* Gray**

*Bazzaniaasymmetrica* (Steph.) N.Kitag. **MOLUCCAS**: Seram: **Herzog 1926b** as *Mastigobryumasymmetricum*.

*Bazzaniacadens* N.Kitag. **MOLUCCAS**: Seram: **Akiyama 1986, 2009**.

*Bazzaniacincinnata* (De Not.) Trevis. **MOLUCCAS**: Seram: **Akiyama 1986, 2009**.

*Bazzaniacommutata* (Lindenb. et Gottsche) Schiffn. **MOLUCCAS**: Seram: **Akiyama 1986, 2009**.

*Bazzaniaconophylla* (Sande Lac.) Schiffn. **MOLUCCAS**: Ambon: **LECTOTYPE** of *Mastigobryumlongidens* [Kitagawa 1973: 263 as “type”], **Stephani 1886a**, 1886b, **1908c** all as *Mastigobryumlongidens*, **Schiffner 1893b, 1898** as *Bazzanialongidens*, Bonner 1963a as *Bazzanialongidens*, Kitagawa 1973. Seram: **Akiyama 1986, 2009**.

*Bazzaniacrassitexta* Steph. **MOLUCCAS**: Ambon: **LECTOTYPE** [Bonner 1963a: 340], **Stephani 1893c, 1908c** as *Mastigobryumcrassitextum*, **Schiffner 1898, Bonner 1963a**, Kitagawa 1979b.

*Bazzaniadistans* (Nees) Trevis. **MOLUCCAS**: Seram: **Akiyama 1986, 2009**.

*Bazzaniaerosa* (Reinw., Blume et Nees) Trevis. **MOLUCCAS**: Del Rosario 1975b. Halmahera: **Meijer 1960**, Kitagawa 1978, Miller et al. 1983, Meagher 2015, Lestari and Ariyanti 2017, Siregar et al. 2018, Haerida et al. 2023. Seram: **Akiyama 1986, 2009**.

*Bazzaniafallax* (Sande Lac.) Schiffn. **MOLUCCAS**: Ambon: **Stephani 1908c** as *Mastigobryumfallax*.

*Bazzaniahorridula* Schiffn. **MOLUCCAS**: Geissler and Bischler 1985 as *Mastigobryumhorridulum*. Ambon: **LECTOTYPE** [Kitagawa 1967: 254], **Schiffner 1893b, 1898, Stephani 1908d** as *Mastigobryumhorridulum*, **Bonner 1963a, Kitagawa 1967**, Grolle 1968b, Del Rosario 1975b, He et al. 2013, Cheah and Yong 2016, Lestari and Ariyanti 2017.

*Bazzaniainsignis* (De Not.) Trevis. **MOLUCCAS**: Ambon: **Schiffner 1893b, 1898, Stephani 1908c** as *Mastigobryuminsigne*, Evans 1932, Del Rosario 1975b. Seram: **Akiyama 1986, 2009**.

*Bazzaniaintermedia* (Gottsche et Lindenb.) Trevis. **MOLUCCAS**: Ambon: **Schiffner 1893b, 1898, Stephani 1908c** as *Mastigobryumintermedium*, **1908d** as *Mastigobryumconcinnum*, Bonner 1963a also as *Bazzaniaconcinna*, Inoue and Miller 1965, Pócs et al. 1967, Pócs 1968, Tixier 1971, Miller et al. 1983 as *Bazzaniaconcinna*, Bapna and Kachroo 2000a. ?Banda: Pócs 1968. Note: we are not aware of any first hand report from Banda,

— var. sarawakiana (De Not.) Schiffn. **MOLUCCAS**: ?Ambon: Bonner 1963a. Note: we are not aware of any first hand report of this variety from **Moluccas** and we are do not know if it is worth recognizing.

*Bazzaniairregularis* (Steph.) Schiffn. **MOLUCCAS**: Ambon: **LECTOTYPE** of *Mastigobryumirregulare* [to be designated], Stephani 1886b, **1908c** both as *Mastigobryumirregulare*, **Schiffner 1898**, Bonner 1963a.

*Bazzaniajavanica* (Sande Lac.) Schiffn. **MOLUCCAS**: Khotimperwati et al. 2018. Seram: **Sande Lacoste 1864** as *Mastigobryumjavanicum*, **Schiffner 1898, Stephani 1908e** as *Mastigobryumjavanicum*, **Meijer 1960**, Kitagawa 1967, Del Rosario 1975b, Miller et al. 1983, Meagher 2015.

*Bazzaniakernii* Steph. **MOLUCCAS**: Seram: **Akiyama 1986, 2009**.

*Bazzaniakokawana* N.Kitag. et T.Kodama. **MOLUCCAS**: Seram: **Akiyama 1986, 2009**.

*Bazzanialongicaulis* (Sande Lac.) Schiffn. **MOLUCCAS**: Buru: **LECTOTYPE** of *Mastigobryumdeningeri* [here designated], **Herzog 1921** as *Mastigobryumdeningeri*, Herzog 1926a as *Mastigobryumdeningeri*. Seram: **Akiyama 1986, 2009**.

*Bazzanialoricata* (Reinw., Blume et Nees) Trevis. **MOLUCCAS**: Kitagawa 1967, Siregar and Pasaribu 2019. Seram: **Akiyama 1986, 2009**.

*Bazzaniamerrillana* (Steph.) Inoue ex Bonner. **MOLUCCAS**: ?Buru: Herzog 1926a as *Mastigobryummerrillanum*. Seram: **Herzog 1921** as *Mastigobryum* ‘*Merillanum*’. Note: we are not aware of any first hand report from Buru.

*Bazzaniaparvitexta* Steph. **MOLUCCAS**: Seram: **Akiyama 1986, 2009**.

*Bazzaniapectinata* (Lindenb. et Gottsche) Schiffn. **MOLUCCAS**: Siregar and Pasaribu 2019. Ambon: **Schiffner 1893b, 1898**, Stephani 1908c as *Mastigobryumpectinatum*, Evans 1932, Bonner 1963a, 1977, Sari 2014, Khotimperwati et al. 2018. Seram: **Akiyama 1986, 2009**.

*Bazzaniapraerupta* (Reinw., Blume et Nees) Trevis. **MOLUCCAS**: Khotimperwati et al. 2018. Seram: **Meijer 1960**, Miller et al. 1983.

*Bazzaniaspiralis* (Reinw., Blume et Nees) Meijer. **MOLUCCAS**: Halmahera: **Meijer 1960**, Miller et al. 1983. Seram: **Akiyama 1986, 2009**.

*Bazzaniastresemannii* (Herzog) N.Kitag. **MOLUCCAS**: Seram: **LECTOTYPE** of *Mastigobryumstresemannii* [here designated], **Herzog 1921**, 1926a both as *Mastigobryumstresemannii*, Kitagawa 1979b.

*Bazzaniasubtilis* (Sande Lac.) Trevis. **MOLUCCAS**: Ambon: **Stephani 1908d** as *Mastigobryumsubtile*, Meijer 1960 [with doubt], Bonner 1963a, Del Rosario 1975b, **Meagher 2010**, Sari 2014, Thouvenot et al. 2018.

Bazzaniatridens(Reinw., Blume et Nees)Trevis.var.tridens. **MOLUCCAS**: Khotimperwati et al. 2018, Siregar and Pasaribu 2019. Ambon: **LECTOTYPE** of *Mastigobryumamboinense* [Kitagawa 1972: 449 as “type”], **Stephani 1924** as *Mastigobryumamboinense*, **Kitagawa 1972** as *Bazzaniaoshimensis*, Miller et al. 1983 as *Bazzaniaaustralis* and *Bazzaniaintermedia*, Sharma and Srivastava 1993. ?Banda: Pócs 1969b. Seram: **Sande Lacoste 1864** as *Mastigobryumtridens*, **Schiffner 1898**, Stephani 1908c as *Mastigobryumtridens*, Evans 1932, **Meijer 1960**, Bonner 1963a, Inoue and Miller 1965, Inoue 1965, Mizutani 1967, Hattori 1968, Del Rosario 1975b, Pócs 1971, Kamimura 1975, Miller et al. 1983, **Akiyama 1986, Akiyama 2009, 2009** both also as *Bazzaniaaustralis*, Sharma and Srivastava 1993, Bapna and Kachroo 2000a, Khotimperwati et al. 2018.

*Bazzaniauncigera* (Reinw., Blume et Nees) Trevis. **MOLUCCAS**: Ambon: **Schiffner 1893b, 1898**, Stephani 1908e as *Mastigobryumuncigerum*, Bonner 1963a, Hattori 1975b, Miller et al. 1983.

*Bazzaniavittata* (Gottsche) Trevis. **MOLUCCAS**: ?Ambon: Stephani 1909a as *Mastigobryumvittatum*, Hattori 1951, Miller et al. 1983, Sari 2014, Siregar et al. 2018. Seram: **Akiyama 1986, 2009**. Note: we are not aware of any first hand report from Ambon.

*Bazzaniawallichiana* (Lindenb.) Trevis. **MOLUCCAS**: Ambon: **Stephani 1908d** as *Mastigobryumwallichianum*, Hattori 1951.

***Calatholejeunea* K.I.Goebel**

*Calatholejeuneaparadoxa* (Schiffn.) K.I.Goebel. **MOLUCCAS**: Ambon: **LECTOTYPE** of *Lejeuneaparadoxa* [Mizutani 1984: 331 as “holotype”], **Schiffner 1893b** as *Lejeuneaparadoxa*, **1898** as *Colurolejeuneaparadoxa*, Stephani 1916 as *Coluraparadoxa*, **Jovet-Ast 1954, Bischler 1961**, Bonner 1963a, 1963b as *Coluraparadoxa*, Grolle 1966a, Schuster 1969, **Mizutani 1984**, Gradstein 1985, Piippo 1992.

***Calypogeia* Raddi**

*Calypogeiagoebelii* (Schiffn.) Steph. **MOLUCCAS**: Seram: **Akiyama 1986, 2009**.

***Caudalejeunea* Schiffn.**

*Caudalejeuneacristiloba* (Steph.) Gradst. **MOLUCCAS**: Mizutani 1977 as *Caudalejeuneacircinata*. ?Ambon: Thiers and Gradstein 1989, Gradstein et al. 2002, Sass-Gyarmati 2003. Seram: Verdoorn 1934a as *Caudalejeuneacircinata*, **1934b** as *Caudalejeuneacircinata*, Jovet-Ast 1958 as *Caudalejeuneacircinata*, Miller et al. 1983 as *Caudalejeuneacircinata*, **Mizutani 1986a** as *Caudalejeuneacircinata*, **Akiyama 2009** as *Caudalejeuneacircinata*. Note: we are not aware of any first hand report from Ambon.

*Caudalejeuneareniloba* (Gottsche) Steph. **MOLUCCAS**: Mizutani 1977, Siregar 2015. Ambon: **LECTOTYPE** of Lopholejeuneasagranavar.dentistipula [here designated], **Sande Lacoste 1864** as *Phragmicomareniloba*, **Schiffner 1890** as *Lopholejeunea* ‘Sagraeana’var.dentistipula, **1898** as *Lopholejeuneadentistipula* and *Thysananthusrenilobus*, **Sydow 1894** as *Lopholejeunea* ‘Sagraeana’var.dentistipula, Miller et al. 1983 also as *Thysananthusrenilobus*, **Geissler and Bischler 1987** as Lejeuneasagranavar.dentistipula, **Mizutani 1988**, Bapna and Kachroo 2000b, **Zhu and Gradstein 2005** as *Lopholejeuneadentistipula*, Siregar et al. 2014, Rosyanti et al. 2018. Seram: **Sande Lacoste 1864** as *Lejeunearecurvistipula* and *Phragmicomareniloba*, **Schiffner 1898** as *Caudalejeunearecurvistipula* and *Thysananthusrenilobus*, Verdoorn 1934a, **1934b**, Jovet-Ast 1958, Miller 1968, Swanson and Miller 1969, Tixier 1970a, 1970b as *Caudalejeunea* ‘*renilyba*’, Miller et al. 1983 also as *Thysananthusrenilobus*, **Mizutani 1986a, Mizutani 1988** also as *Caudalejeunearecurvistipula*, Bapna and Kachroo 2000b, **Akiyama 2009**, Siregar et al. 2014, Siregar 2015, Rosyanti et al. 2018, Ginting and Batubara 2019.

***Cephalozia* (Dumort.) Dumort.**

*Cephaloziastolonacea* (Herzog) Váňa. **MOLUCCAS**: Ambon: **Váňa 1993** as *Metahygrobiellastolonacea*, Schuster 2002 as *Metahygrobiellastolonacea*.

***Ceratolejeunea* (Spruce) J.B.Jack et Steph.**

*Ceratolejeuneabelangeriana* (Gottsche) Steph. **MOLUCCAS**: Seram: **Mizutani 1986a**, Zhu et al. 2005, **Akiyama 2009**.

*Ceratolejeuneamoniliata* Herzog. **MOLUCCAS**: Seram: **Mizutani 1986a**, **Akiyama 2009**.

***Cheilolejeunea* (Spruce) Steph.**

*Cheilolejeuneaceylanica* (Gottsche) R.M.Schust. et Kachroo. **MOLUCCAS**: Mizutani 1978. Bacan: **Hoffmann 1935** as *Pycnolejeuneaceylanica*. Ambon: **Schiffner 1893b** as *Lejeuneaconnivens*, **1898** as *Pycnolejeuneaceylanica*, Tixier 1967b, 1972, 1973b, Kamimura 1974, Miller et al. 1983 also as *Xenolejeuneaceylanica*, **Braggins et al. 2014**. Seram: **Mizutani 1986a**, 2009.

*Cheilolejeuneafalsinervis* (Sande Lac.) R.M.Schust. et Kachroo. **MOLUCCAS**: Bacan: **Zwickel 1932** as *Pycnolejeuneafalsinervis*, **Hoffmann 1935** as *Pycnolejeuneafalsinervis*.

*Cheilolejeuneagigantea* (Steph.) R.M.Schust. et Kachroo. **MOLUCCAS**: Ambon: **LECTOTYPE** of *Pycnolejeuneagigantea* [Thiers 1992: 16 as “holotype”], **Stephani 1896b** as *Pycnolejeuneagigantea*, **Schiffner 1898** as *Pycnolejeuneagigantea*, Stephani 1914 as *Pycnolejeuneagigantea*, **Hoffmann 1935** as *Pycnolejeuneagigantea*, **Thiers 1992**.

*Cheilolejeuneaincisa* (Gottsche) R.M.Schust. et Kachroo. **MOLUCCAS**: Bacan: **Hoffmann 1935** as *Pycnolejeuneaincisa*.

*Cheilolejeuneaintertexta* (Lindenb.) Steph. **MOLUCCAS**: Ambon: **Grolle 1979b**, Mizutani 1982, Miller et al. 1983, Asthana et al. 1995, Zhu and So 1999, Piippo et al. 2002. Seram: **Mizutani 1986a**, **Akiyama 2009**.

*Cheilolejeunealindenbergii* (Gottsche) Mizut. **MOLUCCAS**: Ambon: **LECTOTYPE** of *Euosmolejeuneaintegristipula* [Bonner 1965: 153], **Stephani 1896b** as *Euosmolejeuneaintegristipula*, **Schiffner 1898** as *Euosmolejeuneaintegristipula*, **Bonner 1965** as *Euosmolejeuneaintegristipula*, **Mizutani 1972**, Pócs et al. 1994, Szabó 1997.

*Cheilolejeunealongidens* (Steph.) R.M.Schust. et Kachroo. **MOLUCCAS**: Ambon: **Thiers 1986** as *Pycnolejeuneademissa*.

*Cheilolejeuneaocclusa* (Herzog) T.Kodama et N.Kitag. **MOLUCCAS**: Seram: **Mizutani 1986a**, **Akiyama 2009**.

*Cheilolejeuneatrapezia* (Nees) Kachroo et R.M.Schust. **MOLUCCAS**: Ambon: **Schiffner 1893b** as *Lejeuneatrapezia*, **1898** as *Pycnolejeuneatrapezia*, **Hoffmann 1935** as *Pycnolejeuneameyeniana*, Zhu and Grolle 2004. Seram: **Mizutani 1986a** as *Cheilolejeuneameyeniana* and *Cheilolejeuneaimbricata*, **Akiyama 2009** as *Cheilolejeuneaimbricata* and *Cheilolejeuneameyeniana*.

*Cheilolejeuneatrifaria* (Reinw., Blume et Nees) Mizut. **MOLUCCAS**: Mizutani 1977. Ternate/Tidore: **LECTOTYPE** of *Lejeuneaheterophylla* [to be designated], **Sande Lacoste 1856a, 1856b** both as *Lejeuneaheterophylla*, **Schiffner 1898** as *Euosmolejeuneaheterophylla*, Bonner 1965 as *Euosmolejeuneaheterophylla*. Ambon: **Schiffner 1890** as *Euosmolejeuneatrifaria*, **1898** as *Lejeuneatrifaria*, **Sydow 1894** as *Euosmolejeuneatrifaria*, Miller et al. 1983. Seram: **Sande Lacoste 1864** as *Lejeuneaheterophylla*, **Schiffner 1898** as *Euosmolejeuneaheterophylla*, Bonner 1965 as *Euosmolejeuneaheterophylla*.

*Cheilolejeuneaventricosa* (Schiffn. ex P.Syd.) Xiao L.He. **MOLUCCAS**: Furuki and Yamaguchi 2005, Pócs et al. 2013. Ambon: **LECTOTYPE** of *Pycnolejeuneaventricosa* [here designated], **Schiffner 1890, 1898** both as *Pycnolejeuneaventricosa*, **Sydow 1894** as *Pycnolejeuneaventricosa*, Stephani 1914 as *Pycnolejeuneaventricosa*, **Zhu et al. 2002, Zhu and Lai 2005**, Pócs and Streimann 2006, **Renner 2011**.

*Cheilolejeuneavittata* (Steph. ex G.Hoffm.) R.M.Schust. et Kachroo. **MOLUCCAS**: Seram: **Mizutani 1986a**, Zhu and So 1999, Zhu et al. 2002, **Akiyama 2009**.

***Chiastocaulon* Carl**

*Chiastocaulonbraunianum* (Nees) S.D.F.Patzak, M.A.M.Renner, Schäf.-Verw. et Heinrichs. **MOLUCCAS**: Halmahera: **Sande Lacoste 1864** as *Plagiochilabrauniana*, **Schiffner 1898**, 1900 both as *Plagiochilabrauniana*, Hattori 1952 as *Plagiochilion* ‘*Braunianus*’, Miller et al. 1983 as *Plagiochilionbraunianum*.

*Chiastocaulondendroides* (Nees) Carl. **MOLUCCAS**: Jovet-Ast 1951, Grolle 1965. Bacan: **Carl 1931a**. Ternate/Tidore: **New to Ternate**: Ternate Piek v. Ternate Oosthelling (Ake abdas), 1951.09.02, *D.R. Pleyte 33* (BO11333). Ambon: **Stephani 1903a** as *Plagiochiladendroides*, Bonner 1963b, Miller et al. 1983. Seram: **Inoue 1986** as *Plagiochiladendroides*, **Akiyama 2009** as *Plagiochiladendroides*.

*Chiastocaulonoppositum* (Reinw., Blume et Nees) S.D.F.Patzak, M.A.M.Renner, Schäf.-Verw. et Heinrichs. **MOLUCCAS**: Ternate/Tidore: **LECTOTYPE** of *Plagiochilaopposita* γ *filiformis* [to be designated], **Reinwardt et al. 1824** as *Jungermanniaopposita*, **Lindenberg 1843** as *Plagiochilaopposita* γ *filiformis*, Gottsche et al. 1844 as *Plagiochilaopposita* γ *filiformis*, **Sande Lacoste 1856a** as *Plagiochilaopposite* and *Plagiochilaopposita* γ *filiformis*, **1864** as *Plagiochilaopposita*, **Schiffner 1898** as *Plagiochilaopposite* and Plagiochilaoppositavar.filiformis, Hattori 1942 as *Plagiochilaopposita*, Inoue 1958 as *Noguchiaopposita*, Miller et al. 1983 as *Plagiochilionoppositum*, Zheng et al. 2005 as *Plagiochilionoppositum*. Ambon: **Sande Lacoste 1864** as *Plagiochilaopposita*, **Schiffner 1893b, 1898** both as *Plagiochilaopposita*, Stephani 1904b as *Plagiochilaopposita*, Hattori 1942 as *Plagiochilaopposita*, Inoue 1958 as *Noguchiaopposita*, Miller et al. 1983 as *Plagiochilionoppositum*, Bapna and Kachroo 2000b as *Plagiochilionoppositum*, Zheng et al. 2005 as *Plagiochilionoppositum*. Seram: **Herzog 1921** as *Plagiochilaopposita*, **Carl 1931b** as *Plagiochilaopposita*, **Akiyama 1986, Akiyama 2009** both as *Plagiochilion* ‘*oppositus*’.

***Cladoradula* (Spruce) M.A.M.Renner, Gradst., Ilk.-Borg. et F.R.Oliveira-da-Silva**

*Cladoradulacampanigera* (Mont.) M.A.M.Renner, Gradst., Ilk.-Borg. et F.R.Oliveira-da-Silva. **MOLUCCAS**: Seram: **Akiyama 1986, 2009** both as *Radulacampanigera*, Yamada 1989 as *Radulacampanigera*.

***Cololejeunea* (Spruce) Steph.**

*Cololejeuneaaequabilis* (Sande Lac.) Schiffn. **MOLUCCAS**: Inoue and Miller 1965 as *Cololejeuneayulensis*, Miller et al. 1983 as *Cololejeuneayulensis*. Seram: **Mizutani 1986a** as *Cololejeuneayulensis*, **Akiyama 2009** as *Cololejeuneayulensis*.

*Cololejeuneaangustiflora* (Steph.) Mizut. **MOLUCCAS**: Seram: **Mizutani 1986a** as *Cololejeuneacrenulata* and *Cololejeuneajavanica*, **Akiyama 2009** as *Cololejeuneajavanica* and *Cololejeuneacrenulata*.

*Cololejeuneadozyana* (Sande Lac.) Schiffn. **MOLUCCAS**: Bonner 1963b, Mizutani 1977, Tixier 1980, Pócs and Piippo 2011. Bacan: **Benedix 1953**. Seram: **Mizutani 1986a**, Zhu and So 1998b, Zhu and So 1999, **Akiyama 2009**, Müller et al. 2016.

*Cololejeuneaequialbi* Tixier. **MOLUCCAS**: Pócs et al. 2011. Seram: **Mizutani 1986a**, Pócs et al. 1994, Zhu and So 2001, Eggers 2006, **Akiyama 2009** as *Cololejeunea* ‘*equalbi*’, Pócs and Piippo 2011, Pócs and Podani 2015.

*Cololejeuneafalcata* (Horik.) Benedix. **MOLUCCAS**: Seram: **Mizutani 1986a** as *Cololejeuneafalcatoides*, **Akiyama 2009** as *Cololejeuneafalcatoides*.

*Cololejeuneafloccosa* (Lehm. et Lindenb.) Schiffn. **MOLUCCAS**: Seram: **Mizutani 1986a**, **Akiyama 2009**.

*Cololejeuneahamata* Steph. **MOLUCCAS**: Ambon: **LECTOTYPE** [Bonner 1963b: 830], **Stephani 1895, Schiffner 1898**, Stephani 1916 as *Physocoleahamata*, **Bonner 1963b**.

*Cololejeuneahaskarliana* (Lehm.) Schiffn. **MOLUCCAS**: Seram: **Mizutani 1986a**, **Akiyama 2009** as *Cololejeunea* ‘*hasskarliana*’.

*Cololejeuneainflectens* (Mitt.) Benedix. **MOLUCCAS**: Jovet-Ast and Tixier 1962 as *Cololejeuneaciliatilobula*, Tixier 1967b, 1971, 1979 all as *Cololejeuneaciliatilobula*, Mizutani 1975 as *Campylolejeuneainflectens*, Bapna and Kachroo 2000b as *Campylolejeuneainflectens*. Ambon: Stephani 1916 as *Physocoleaciliatilobula*, **Benedix 1953** as *Cololejeuneaciliatilobula*, **Bischler 1961** as *Campylolejeuneaciliatilobula*, Bonner 1963a as *Campylolejeuneaciliatilobula*, 1963b as *Cololejeuneaciliatilobula*. Seram: **Mizutani 1986a** as *Cololejeuneapeculiaris*, **Akiyama 2009** as *Cololejeuneapeculiaris*.

*Cololejeuneakoponenii* (Pócs) Pócs. **MOLUCCAS**: Seram: **Bi et al. 2019**.

*Cololejeunealanciloba* Steph. **MOLUCCAS**: Seram: **Mizutani 1986a**, Pócs et al. 1994, **Akiyama 2009**.

*Cololejeuneametzgeriopsis* (K.I.Goebel) Gradst., R.Wilson, Ilk.-Borg. et Heinrichs. **MOLUCCAS**: Miller et al. 1983 as *Metzgeriopsispusilla*. Bacan: Schiffner 1893a as *Metzgeriopsispusilla*, **Schiffner 1893c** as *Lejeuneapusilla*, **1898** as *Metzgeriopsispusilla*, Stephani 1916 as *Metzgeriopsispusilla*, Gradstein et al. 2006. Seram: **Mizutani 1986a** as *Metzgeriopsispusilla*, Gradstein et al. 2006, **Akiyama 2009** as *Metzgeriopsispusilla*.

*Cololejeuneaobliqua* (Nees et Mont.) Schiffn. **MOLUCCAS**: Seram: **Mizutani 1986a** as *Cololejeuneanymannii*, **Akiyama 2009** as *Cololejeunea* ‘*nymanii*’.

*Cololejeuneapapillosa* (K.I.Goebel) Mizut. **MOLUCCAS**: Seram: **Mizutani 1986a**, **Akiyama 2009**.

*Cololejeuneaplagiophylla* Benedix. **MOLUCCAS**: Seram: **Mizutani 1986a**, **Akiyama 2009**.

Cololejeuneaplanissima(Mitt.)Abeyw.var.planissima. **MOLUCCAS**: Seram: **Mizutani 1986a** as *Cololejeuneaplanissima*, **Akiyama 2009** as *Cololejeuneaplanissima*.

*Cololejeunearaduliloba* Steph. **MOLUCCAS**: Seram: **Mizutani 1986a**, **Akiyama 2009**.

*Cololejeuneastephanii* Schiffn. ex Benedix. **MOLUCCAS**: Seram: **Mizutani 1986a**, **Akiyama 2009**.

*Cololejeuneatriapiculata* (Herzog) Tixier. **MOLUCCAS**: Seram: **Mizutani 1986a**, **Akiyama 2009**.

*Cololejeuneatrichomanis* (Gottsche) Besch. **MOLUCCAS**: Seram: **Mizutani 1986a** as *Cololejeuneagoebelii*, **Akiyama 2009** as *Cololejeuneagoebelii*.

***Colura* (Dumort.) Dumort.**

*Coluraacroloba* (Prantl) Jovet-Ast. **MOLUCCAS**: Seram: **Mizutani 1986a**, Eggers et al. 1998, **Akiyama 2009**.

*Coluraamboinensis* Steph. **MOLUCCAS**: Ambon: **LECTOTYPE** [to be designated], ORIGINAL MATERIAL of Colurolejeuneasuperbavar.typica, **Schiffner 1890** as ‘*Coluro-Lejeunea*’ *superba* var. typica, **Stephani 1916**, Bonner 1963b, Miller et al. 1983 as *Colurolejeuneasuperba*.

*Coluraari* (Steph.) Steph. **MOLUCCAS**: Halmahera: **Jovet-Ast 1953**, Bonner 1963b, Grolle 1965, Pócs et al. 1967, Tixier 1967a, Onraedt 1979, Miller et al. 1983. Seram: **Mizutani 1986a**, **Akiyama 2009**.

*Coluraconica* (Sande Lac.) K.I.Goebel. **MOLUCCAS**: Seram: **Mizutani 1986a**, **Akiyama 2009**.

*Coluraimperfecta* Steph. **MOLUCCAS**: Seram: **Mizutani 1986a**, **Akiyama 2009**.

*Colurakarstenii* K.I.Goebel. **MOLUCCAS**: Ambon: **LECTOTYPE** [Jovet-Ast 1953: 230, Bonner 1963b: 869], **Stephani 1896b** as *Colurolejeuneakarstenii*, 1916, **Schiffner 1898** as *Colurolejeuneakarstenii*, Schiffner and Stephani 1901 as Colurolejeuneakarsteniivar.latifolia, **Jovet-Ast 1953, Bonner 1963b**, Tixier 1970a, **Zhu and So 2001**, Pócs 2013, **Sangrattanaprasert et al. 2018**.

*Coluramaxima* Jovet-Ast. **MOLUCCAS**: Halmahera: **LECTOTYPE** [to be designated], **Jovet-Ast 1953, Bonner 1963b**.

*Coluraornata* K.I.Goebel. **MOLUCCAS**: Pócs and Ninh 2012. Seram: **Mizutani 1986a**, Pócs et al. 1994, **Akiyama 2009**.

*Colurasuperba* (Mont.) Steph. **MOLUCCAS**: Reiner-Drehwald 1999 as Lejeuneasuperbavar.typica. Ambon: Bonner 1963b as *Colurolejeuneasuperba*, **Geissler and Bischler 1987** as Lejeuneasuperbavar.typica. Seram: **Mizutani 1986a**, **Akiyama 2009**.

***Conoscyphus* Mitt.**

*Conoscyphustrapezioides* (Sande Lac.) Schiffn. **MOLUCCAS**: Seram: **Akiyama 1986, Akiyama 2009**.

*Cryptolophocolea* L.Söderstr., Crand.-Stotl., Stotler et Váňa

*Cryptolophocoleacostata* (Nees) L.Söderstr. **MOLUCCAS**: Seram: **Akiyama 1986, 2009** both as *Lophocoleagiulianettii*.

***Cuspidatula* Steph.**

*Cuspidatulacontracta* (Reinw., Blume et Nees) Steph. **MOLUCCAS**: Ambon: **Schiffner 1893b, 1898** both as *Anastrophyllumcontractum*, Schiffner 1900 as *Anastrophyllumcontractum*, **Stephani 1901c**, Bonner 1962b as *Anastrophyllumcontractum*, Bonner 1963b, Kitagawa 1970, Grolle 1971, Bizot and Pócs 1974, Miller et al. 1983, Engel and Glenny 2008. Seram: **Akiyama 1986** as *Jamesoniellacontracta*, **Akiyama 2009** as *Jungermanniacontracta*.

*Cuspidatulaflaccida* (Steph.) K.Feldberg, Váňa, Hentschel et Heinrichs. **MOLUCCAS**: Seram: **Akiyama 1986, 2009** both as *Anomacaulisflaccidus*.

*Cuspidatulaflexicaulis* (Nees) Váňa et L.Söderstr. **MOLUCCAS**: Miller et al. 1983 as *Jamesoniellaflexicaulis*. Ambon: **Grolle 1971** as *Jamesoniellaflexicaulis*. Seram: **Akiyama 1986, 2009** both as *Jungermanniaflexicaulis*.

***Denotarisia* Grolle**

*Denotarisialinguifolia* (De Not.) Grolle. **MOLUCCAS**: Ambon: **LECTOTYPE** of *Jungermanniaovifolia* [Grolle 1971: 11 as “holotype”], **Schiffner 1893b, 1898**, 1900 all as *Jamesoniellaovifolia*, **Stephani 1901b** as *Jamesoniellaovifolia*, Grolle 1966a as *Jamesoniellaovifolia*, **1971, Bonner 1976** as *Jungermanniaovifolia*, Miller et al. 1983 also as *Jamesoniellaovifolia* and *Jungermanniaovifolia*, Váňa and Piippo 1989b, Váňa 1991b. Seram: **Akiyama 1986, 2009**.

***Dinckleria* Trevis.**

*Dinckleriasingularis* (Schiffn.) M.A.M.Renner, Schäf.-Verw. et Heinrichs. **MOLUCCAS**: Ternate/Tidore: **Gottsche et al. 1844** as *Plagiochilatrapezoidea* β *tenera*, **Sande Lacoste 1856a**, 1856b both as *Plagiochilatrapezoidea* β *tenera*, **Schiffner 1898** as Plagiochilatrapezoideavar.tenera. Seram: **So 2000a, 2001a, 2001b** all as *Plagiochilasingularis*, Renner et al. 2016.

***Diplasiolejeunea* (Spruce) Schiffn.**

*Diplasiolejeuneacavifolia* Steph. **MOLUCCAS**: Seram: **Mizutani 1986a**, **Akiyama 2009**.

*Diplasiolejeuneapatelligera* Herzog. **MOLUCCAS**: Seram: **Mizutani 1986a**, **Akiyama 2009**.

***Drepanolejeunea* (Spruce) Steph.**

DrepanolejeuneablumeiSteph.var.angustistipa Herzog. **MOLUCCAS**: Bonner 1965. Bacan: **Herzog 1936**.

*Drepanolejeuneadactylophora* (Nees, Lindenb. et Gottsche) J.B.Jack et Steph. **MOLUCCAS**: Mizutani 1961 as *Drepanolejeuneadactylophora*, Inoue 1965, Kamimura 1974, Mizutani 1975 as *Drepanolejeuneadactylophora*, Mizutani 1977 as *Drepanolejeuneadactylophora*, Mizutani 1978 as *Drepanolejeuneadactylophora*. Bacan: **Schiffner 1893c**. ?Ambon: Stephani 1913 as *Drepanolejeuneadactylophora*, Tixier 1962 as *Drepanolejeuneadactylophora*, 1972, Bonner 1965 as *Drepanolejeuneadactylophora*, Pócs et al. 1967, Miller et al. 1983 as *Drepanolejeuneadactylophora*, Zhu and So 2001 as *Drepanolejeuneadactylophora*. Seram: **Mizutani 1986a** as *Drepanolejeuneadactylophora*, Zhu and So 2001 as *Drepanolejeuneadactylophora*, **Akiyama 2009**. Note: we are not aware of any first hand report from Ambon.

*Drepanolejeuneafoliicola* Horik. **MOLUCCAS**: Seram: **Mizutani 1986a** as *Leptolejeuneafoliicola*.

*Drepanolejeuneagrandis* Herzog. **MOLUCCAS**: **LECTOTYPE** [to be designated], **Herzog 1939, Bonner 1965**, Pócs et al. 1995, Pócs et al. 2019a. Seram: **Mizutani 1986a**, **Akiyama 2009**.

*Drepanolejeuneahampeana* Steph. **MOLUCCAS**: Ambon: Stephani 1913, Bonner 1965, Miller et al. 1983.

*Drepanolejeuneaintermedia* Zwickel. **MOLUCCAS**: O’Shea et al. 1997. Bacan: **Grolle 1976**. Seram: **Mizutani 1986a**, **Akiyama 2009**.

*Drepanolejeunealevicornua* Steph. **MOLUCCAS**: Seram: **Mizutani 1986a** as Drepanolejeunealevicornuavar.levicornua, **1990**, Streimann 1991, Szabó 1997, Eggers et al. 1998, Zhu and So 2001, **Akiyama 2009** as Drepanolejeunealevicornuavar.levicornua, Siregar et al. 2017, Pócs et al. 2019b, Susilo et al. 2023.

*Drepanolejeunealongicornua* (Herzog) Mizut. **MOLUCCAS**: Seram: **Mizutani 1986a** as Drepanolejeunealevicornuavar.longicornua, **1990, Akiyama 2009** as Drepanolejeunealevicornuavar.longicornua.

*Drepanolejeunealongicruris* (Steph.) Grolle et R.L.Zhu. **MOLUCCAS**: Seram: **Mizutani 1986a** as *Rhaphidolejeunealongicruris*, Grolle and Zhu 2000, **Akiyama 2009** as *Rhaphidolejeunealongicruris*.

*Drepanolejeunealyrata* Grolle. **MOLUCCAS**: Seram: **Mizutani 1986a**, **Akiyama 2009**.

*Drepanolejeuneamoluccensis* Herzog. **MOLUCCAS**: Bacan: **LECTOTYPE** [Nadhifah et al. 2021: 6], **Herzog 1934, Nadhifah et al. 2021**.

*Drepanolejeuneaobliqua* Steph. **MOLUCCAS**: Pócs et al. 1967. Bacan: **Herzog 1939**.

*Drepanolejeuneapentadactyla* (Mont.) Steph. **MOLUCCAS**: Jovet-Ast 1958 as *Drepanolejeuneamicholitzii*, Jovet-Ast and Tixier 1962 as Drepanolejeuneamicholitziivar.genuina, Bonner 1965 as Drepanolejeuneamicholitziivar.genuina, Miller 1968 as *Drepanolejeuneamicholitzii*, Tixier 1970b as *Drepanolejeuneamicholitzii*, Kitagawa 1981 as *Drepanolejeuneamicholitzii*, Miller et al. 1983 as *Drepanolejeuneamicholitzii*. Bacan: **ORIGINAL MATERIAL** of Drepanolejeuneamicholitziivar.genuina, **Herzog 1934** as Drepanolejeuneamicholitziivar.genuina. Seram: **Mizutani 1986a** as *Drepanolejeuneamicholitzii*, Zhu and So 2001, **Akiyama 2009** as *Drepanolejeuneamicholitzii*, Asthana and Shukla 2009, Dey et al. 2013, Susilo et al. 2023.

— var. dactylophoroides (Herzog) Pócs. ?**MOLUCCAS**: Tixier 1979 as Drepanolejeuneamicholitziivar.dactylophoroides. Note: we are not aware of any first hand report of this variety from Moluccas although it is reported to occur on neighbouring islands.

*Drepanolejeuneaspinosocornuta* Steph. **MOLUCCAS**: Seram: **Mizutani 1986a**, **1990, Akiyama 2009**, Pócs et al. 2019a.

*Drepanolejeuneatenera* K.I.Goebel. **MOLUCCAS**: Jovet-Ast 1958, Bonner 1965 as Drepanolejeuneateneravar.genuina, Pócs 1969a, Tixier 1974b. Bacan: **ORIGINAL MATERIAL** of Drepanolejeuneateneravar.genuina, **Herzog 1934** as Drepanolejeuneateneravar.genuina. Seram: **Mizutani 1986a**, **Akiyama 2009**.

*Drepanolejeuneaternatensis* (Gottsche) Schiffn. **MOLUCCAS**: Inoue 1965, Miller 1968, Pócs 1969a, Kamimura 1974, Mizutani 1975, 1977, 1978, Udar and Awasthi 1982 as Drepanolejeuneaternatensisvar.ternatensis, Bapna and Kachroo 2000b as Drepanolejeuneaternatensisvar.ternatensis, Xiong and Cao 2018, Siregar et al. 2020a. Bacan: **Herzog 1939** as Drepanolejeuneaternatensisvar.ternatensis as *Drepanolejeuneaternatensis*. Ternate/Tidore: **LECTOTYPE** of *Lejeuneaternatensis* [to be designated], **Gottsche et al. 1845b** as *Lejeuneaternatensis*, **Sande Lacoste 1856a**, 1856b both as *Lejeuneaternatensis*, **Stephani 1890b** as *Lejeuneaternatensis*, **Schiffner 1898, Mizutani 1961**, Miller et al. 1983 as *Lejeuneaternatensis*, *Microlejeuneaternatensis* and *Drepanolejeuneaternatensis*, **Zhu and So 2001, Dey et al. 2013**. Seram: **Mizutani 1986a** as *Drepanolejeuneaternatensis*, Zhu and So 2001, **Akiyama 2009**, Dey et al. 2013, Siregar et al. 2017, Susilo et al. 2023.

Drepanolejeuneathwaitesiana(Mitt.)Steph.var.thwaitesiana. **MOLUCCAS**: Seram: **Mizutani 1990**, Zhu and So 2001, Haerida 2009, Siregar et al. 2017, Susilo et al. 2023.

*Drepanolejeuneatricornua* Herzog. **MOLUCCAS**: Seram: **LECTOTYPE** [Bonner 1965: 105], **Herzog 1936, Bonner 1965**, Tixier 1979, Mizutani 1990, **Pócs et al. 2011**, Pócs et al. 2013, Pócs et al. 2014, Siregar et al. 2017, **Samarakkody et al. 2018**, Kasiani et al. 2019.

***Dumortiera* Nees**

*Dumortierahirsuta* (Sw.) Nees. **MOLUCCAS**: Ambon: **Sopacua et al. 2020** as *Dumortiera* ‘*hirsute*’ [Saparua I]. Note: we are not aware of any first hand report from **Moluccas** although it is reported to occur on neighbouring islands.

***Frullania* Raddi**

*Frullaniaakiyamae* S.Hatt. **MOLUCCAS**: Seram: HOLOTYPE, **Hattori 1986b, Engel 1993, Crosby and Engel 2006, Akiyama 2009**.

*Frullaniaapiculata* (Reinw., Blume et Nees) Nees. **MOLUCCAS**: Verdoorn 1930, Inoue and Miller 1965, Inoue 1965, Pócs et al. 1967, Miller 1968, Swanson and Miller 1969, Tixier 1972, Haerida et al. 2023. Halmahera: **Sande Lacoste 1864**, **Schiffner 1898, Umagap 2019**. Ambon: **Schiffner 1890, 1898, Sydow 1894**. Seram: **Sande Lacoste 1864, Schiffner 1898, Hattori 1986b, Akiyama 2009**.

— var. apiculata. **MOLUCCAS**: Ambon: Schiffner 1893b, Miller et al. 1983. Seram: Miller et al. 1983.

— var. goebelii Schiffn. **MOLUCCAS**: Tixier 1971. Ambon: **LECTOTYPE** of *Frullaniakarstenii* [to be designated], **Schiffner 1893b, 1898** both as *Frullaniakarstenii*, Stephani 1911 as *Frullaniakarstenii*, **Bonner 1965** as *Frullaniakarstenii*.

*Frullaniaarmatifolia* Verd. **MOLUCCAS**: Seram: **Hattori 1986b, Akiyama 2009**.

*Frullaniaattenuata* Steph. **MOLUCCAS**: Seram: **Hattori 1986b, Akiyama 2009**.

*Frullaniaberthoumieui* Steph. **MOLUCCAS**: Ambon: **Hattori 1975a**, 1976, Hattori et al. 1977, Bapna and Kachroo 2000b.

*Frullaniaclaviloba* Steph. **MOLUCCAS**: Ambon: **LECTOTYPE** [Bonner 1965: 266], **Stephani 1911, Verdoorn 1930, Bonner 1965**, Pócs et al. 1967, Kamimura 1974, Hattori 1975a, **1975d**, 1976, 1982, Hattori et al. 1977, **Sukkharak 2018**.

*Frullaniacordistipula* (Reinw., Blume et Nees) Nees. **MOLUCCAS**: Halmahera: **Sande Lacoste 1864**, **Schiffner 1898**, Merrill and Merritt 1910, Hattori 1977, Miller et al. 1983.

— var. dentistipula S.Hatt. **MOLUCCAS**: Seram: **HOLOTYPE**, **Hattori 1986b, Engel 1993, Crosby and Engel 2006, Akiyama 2009**.

*Frullaniaericoides* (Nees) Mont. **MOLUCCAS**: Verdoorn 1930 as Frullaniasquarrosavar.planescens, Bonner 1965 as Frullaniasquarrosaf.campanuloides, Bai 2002 as Frullaniaericoidesvar.planescens, Haerida et al. 2023. Seram: **Sande Lacoste 1864** as *Frullaniasquarrosa*, **Schiffner 1898** as *Frullaniasquarrosa*, Miller et al. 1983 as *Frullaniasquarrosa*, **Hattori 1986b, Akiyama 2009**.

*Frullaniafallax* Gottsche. **MOLUCCAS**: Halmahera: **Sande Lacoste 1864**, **Schiffner 1898**, Hattori 1975a as Frullaniafallaxvar.fallax.

*Frullaniagaudichaudii* (Nees et Mont.) Nees et Mont. **MOLUCCAS**: Ambon: **Schiffner 1893b**, Schiffner and Stephani 1901, **Verdoorn 1930**, Bonner 1965, Swanson and Miller 1969, Kamimura 1971, Miller et al. 1983, Haerida et al. 2023. Seram: **Hattori 1986b, Akiyama 2009**.

*Frullaniagracilis* (Reinw., Blume et Nees) Nees. **MOLUCCAS**: Ambon: **LECTOTYPE** of *Frullanialacerifolia* [Bonner 1965: 345], **Stephani 1911** as *Frullanialacerifolia*, Verdoorn 1930 as Frullaniagracilisvar.lacerifolia, **Bonner 1965** as Frullaniagracilisvar.lacerifolia and *Frullanialacerifolia*, Kamimura 1974, Miller et al. 1983. Seram: **Hattori 1986b**.

— var. gracilis. **MOLUCCAS**: Seram: **Akiyama 2009**.

— var. vittata S.Hatt. **MOLUCCAS**: Seram: **HOLOTYPE, Hattori 1986b, Engel 1993, Crosby and Engel 2006, Akiyama 2009**.

*Frullaniagrandistipula* Lindenb. **MOLUCCAS**: Bacan: **Verdoorn 1930**. Halmahera: **Sande Lacoste 1864** as *Frullaniarugosa*, **Schiffner 1898** as *Frullaniarugosa*, Pócs et al. 1967, Hattori 1974b, 1975a, 1976 as Frullaniagrandistipulavar.grandistipula, 1979, 1982, Miller et al. 1983, Hattori and Piippo 1986.

*Frullaniahasskarliana* Lindenb. **MOLUCCAS**: Haerida et al. 2023.

— var. gracilis S.Hatt. **MOLUCCAS**: Seram: **HOLOTYPE, Hattori 1986b, Engel 1993, Crosby and Engel 2006, Akiyama 2009**.

— var. hasskarliana. **MOLUCCAS**: Seram: **Hattori 1986b, Akiyama 2009**.

— var. parvidentata S.Hatt. **MOLUCCAS**: Seram: **HOLOTYPE, Hattori 1986b, Engel 1993, Crosby and Engel 2006, Akiyama 2009**.

*Frullaniaheteromorpha* Schiffn. **MOLUCCAS**: Seram: **Hattori 1986b, Akiyama 2009**.

*Frullaniaintegristipula* (Nees) Nees

— var. emarginata Verd. **MOLUCCAS**: Halmahera: **Verdoorn 1930, Hattori 1980**, Hattori 1982, Hattori and Piippo 1986.

— var. integristipula. **MOLUCCAS**: Halmahera: **Hattori 1980** as *Frullaniaintegristipula*.

*Frullaniaintermedia* (Reinw., Blume et Nees) Nees. **MOLUCCAS**: Inoue and Miller 1965, Bapna and Kachroo 2000b. Seram: Miller et al. 1983.

— subsp. intermedia. **MOLUCCAS**: Ambon: **LECTOTYPE** of *Frullaniaamboinensis* [Bonner 1965: 228], **LECTOTYPE** of *Frullaniabillardiereana* [Bonner 1965: 248], **Montagne 1843, 1856** both as *Frullaniabillardiereana*, **Gottsche et al. 1845b** as *Frullaniabillardiereana*, **Schiffner 1890, 1898** both as *Frullaniaamboinensis*, **1898** as *Frullaniabillardiereana*, **Sydow 1894** as *Frullaniaamboinensis*, Schiffner and Stephani 1901 as *Frullaniaamboinensis*, Stephani 1911 as *Frullaniaamboinensis*, Verdoorn 1930 as *Frullaniaintermedia* f. ‘*billardieriana*’, **Verdoorn 1930** as Frullaniaintermediavar.amboinensis, **Bonner 1965** as *Frullaniaintermedia* f. ‘*billardierianaa*’, Bonner 1965 as Frullaniaintermediavar.amboinensis, *Frullaniaamboinensis* and *Frullaniabillardiereana*, Hattori 1976 as *Frullaniaintermedia* f. ‘*billardierianaa*’, **1980** as *Frullaniabillardiereana*, 1982, 1986 both as *Frullaniaintermedia* f. ‘*billardierianaa*’, Miller et al. 1983 as *Frullaniaintermedia* f. ‘*billardierana*’. Seram: **Sande Lacoste 1864** as *Frullaniabillardiereana*, **Schiffner 1898** as *Frullaniabillardiereana*, **Verdoorn 1930** as *Frullaniaintermedia* f. ‘*billardierianaa*’, Bonner 1965 as *Frullaniaintermedia* f. ‘*billardierianaa*’, Hattori 1976 as *Frullaniaintermedia* f. ‘*billardieriana*’, 1980 as *Frullaniabillardiereana*, 1982, 1986 both as *Frullaniaintermedia* f. ‘*billardieriana*’, Miller et al. 1983 as *Frullaniaintermedia* f. ‘*billardierana*’.

*Frullaniajunghuhniana* Gottsche

— var. junghuhniana. **MOLUCCAS**: Seram: **Hattori 1986b, Akiyama 2009**.

— var. tenella (Sande Lac.) Grolle et S.Hatt. **MOLUCCAS**: Seram: **Hattori 1986b, Akiyama 2009**.

*Frullaniamultilacera* Steph. **MOLUCCAS**: Seram: **Hattori 1986b, Akiyama 2009**.

*Frullanianepalensis* (Spreng.) Lehm. et Lindenb. **MOLUCCAS**: Bacan: **Verdoorn 1930**, Hattori 1952, 1973b, Kamimura 1961, Pócs et al. 1967.

*Frullanianigricaulis* (Reinw., Blume et Nees) Nees. **MOLUCCAS**: Halmahera: **Verdoorn 1930**, Hattori 1974b, 1975c, Hattori et al. 1977.

FrullanianobilisSteph.var.cochleata (Steph.) S.Hatt. **MOLUCCAS**: Seram: **Hattori 1986b, Akiyama 2009**.

*Frullanianodulosa* (Reinw., Blume et Nees) Nees. **MOLUCCAS**: Verdoorn 1930 as *Frullanianodulosa* β *plana*, Frullanianodulosaf.dapitana and Frullanianodulosaf.irreflexa, Kamimura 1943 as Frullanianodulosaf.dapitana, Bonner 1965 as Frullanianodulosaf.dapitana, Bonner 1965 as Frullanianodulosaf.irreflexa, Miller et al. 1983 as *Frullaniadapitana* and Frullanianodulosaf.irreflexa, Haerida et al. 2023. Morotai: **Hattori 1975a, Hattori 1980** as Frullanianodulosavar.nodulosa, Miller et al. 1983. Ambon: **LECTOTYPE** of *Frullanianodulosa* β *plana* [to be designated], **Sande Lacoste 1864** also as *Frullaniathuillieri*, **Schiffner 1890** as Frullanianodulosavar.nodulosa and *Frullanianodulosa* β *plana*, **1893b, 1898** also as *Frullaniareplicate* and *Frullanianodulosa* β *plana*, **Sydow 1894** as *Frullanianodulosa* β *plana*, **Verdoorn 1930, 1932** as Frullanianodulosaf.irreflexa, Hattori 1951, **Bonner 1965** as *Frullanianodulosa* β *plana*, Swanson and Miller 1969, **Hattori 1975a, 1980** as Frullanianodulosavar.nodulosa, Miller et al. 1983. Seram: **Sande Lacoste 1864, Verdoorn 1930**, Hattori 1951, Swanson and Miller 1969, **Hattori 1980** as Frullanianodulosavar.nodulosa, **1986b**, Miller et al. 1983, **Akiyama 2009**.

*Frullaniaorientalis* Sande Lac. **MOLUCCAS**: Bapna and Kachroo 2000b, Haerida et al. 2023. Seram: **Hattori 1986b, Akiyama 2009**.

*Frullaniaornithocephala* (Reinw., Blume et Nees) Nees. **MOLUCCAS**: Haerida 2015. Ambon: Merrill and Merritt 1910, **Verdoorn 1930**, Bonner 1965, Hattori 1974c, 1975c, 1976, Hattori et al. 1977. Seram: **Verdoorn 1930**, Bonner 1965, Hattori 1974c, 1975c, 1976, Hattori et al. 1977.

— var. major (Nees) Schiffn. **MOLUCCAS**: Ambon: **Schiffner 1893b, 1898**.

— var. ornithocephala. **MOLUCCAS**: Ambon: Hattori 1974a, 1975a. Seram: Hattori 1974a, 1975a as *Frullaniaornithocephala*.

*Frullaniapauciramea* Steph. **MOLUCCAS**: Seram: **Hattori 1986b, Akiyama 2009**.

FrullaniapiptophyllaS.Hatt.var.minor S.Hatt. **MOLUCCAS**: Seram: **HOLOTYPE, Hattori 1986b, Engel 1993, Crosby and Engel 2006, Akiyama 2009**.

*Frullaniapulogensis* Steph. **MOLUCCAS**: Seram: **Hattori 1986b, Akiyama 2009**.

*Frullaniaramuligera* (Nees) Mont. **MOLUCCAS**: Seram: **Hattori 1986b, Akiyama 2009**.

FrullaniareflexistipulaSande Lac.var.reflexistipula**MOLUCCAS**: Haerida et al. 2023. Seram: **Sande Lacoste 1864**, **Schiffner 1898, Verdoorn 1930**, Hattori 1973b, 1974a, 1974c, 1975a, 1976, 1982, **1986b**, Miller et al. 1983, Hattori and Piippo 1986, **Akiyama 2009**.

*Frullaniarepandistipula* Sande Lac. **MOLUCCAS**: Ambon: Stephani 1911, Verdoorn 1930 [rejecting Stephani’s reports], Bonner 1965, Hattori 1973a [rejecting earlier reports], Bizot et al. 1978. Seram: **Hattori 1986b, Akiyama 2009**. Note: the only report from Ambon is rejected as *Frullaniatricarinata* by Verdoorn (1930).

*Frullaniaserrata* Gottsche. **MOLUCCAS**: Pócs 1971, Hattori 1974a, Miller et al. 1983. Bacan: **Verdoorn 1930**.

— var. ceramensis S.Hatt. **MOLUCCAS**: Seram: **HOLOTYPE, Hattori 1986b, Engel 1993, Crosby and Engel 2006, Akiyama 2009**.

— var. hamatispina (S.Hatt.) S.Hatt. **MOLUCCAS**: Seram: **Hattori 1986b, Akiyama 2009**.

— var. serrata. **MOLUCCAS**: Seram: **Hattori 1986b** as Frullaniaserrataf.crispulo-dentata, **Akiyama 2009** as Frullaniaserrataf.crispulo-dentata.

*Frullaniasubcaduca* S.Hatt. **MOLUCCAS**: Seram: **Hattori 1986b, Akiyama 2009**.

*Frullaniasublignosa* Steph. **MOLUCCAS**: Seram: **Hattori 1986b, Akiyama 2009**.

FrullaniasubnigricaulisS.Hatt.var.subtruncata S.Hatt. **MOLUCCAS**: Seram: **Hattori 1986b, Akiyama 2009**.

*Frullaniasubocellata* S.Hatt. **MOLUCCAS**: Seram: **HOLOTYPE, Hattori 1986b**, 1988, **Engel 1993, Crosby and Engel 2006, Akiyama 2009**, Pócs et al. 2014.

*Frullaniaternatensis* Gottsche. **MOLUCCAS**: Haerida 2015, Haerida et al. 2023. Bacan: **Verdoorn 1930**, Tixier 1962, Kamimura 1974. Ternate/Tidore: **LECTOTYPE** [Bonner 1965: 451], **Gottsche et al. 1846, Sande Lacoste 1856a**, 1856b, **Schiffner 1898, Verdoorn 1930**, Tixier 1962, 1970a, 1971, 1972, Kamimura 1974, Miller et al. 1983. Ambon: **Verdoorn 1932**. Seram: **Verdoorn 1932**, Miller et al. 1983.

— var. non-appendiculata S.Hatt. **MOLUCCAS**: Seram: **Hattori 1986b, Akiyama 2009**.

— var. ternatensis. **MOLUCCAS**: Ternate/Tidore: Hattori 1974a. Seram: **Hattori 1975a**.

*Frullaniatricarinata* Sande Lac. **MOLUCCAS**: Ambon: **Schiffner 1893b, 1898**, Verdoorn 1930.

*Frullaniatrichodes* Mitt. **MOLUCCAS**: Ambon: **LECTOTYPE** of *Frullaniapicta* [Bonner 1965: 400; here affirmed], **Stephani 1894a** as *Frullaniapicta*, **Schiffner 1898** as *Frullaniapicta*, **Verdoorn 1930** as *Frullaniapicta*, Svihla 1955, 1959 both as *Frullaniapicta*, Kamimura 1961, 1974 both as *Frullaniatenuicaulis*, Arnell 1965 as *Frullaniapicta*, **Bonner 1965** as *Frullaniapicta*, Kitagawa 1969 as *Frullaniapicta*, Tixier 1973b as *Frullaniatenuicaulis*, 1974b as *Frullaniapicta*.

*Frullaniavaginata* (Sw.) Nees. **MOLUCCAS**: Ternate/Tidore: **Reinwardt et al. 1824** as *Jungermanniavaginata*, **Nees 1830** as *Jungermanniavaginata*. Ambon: **Sande Lacoste 1864** [Saparoea I], **Verdoorn 1930**, Hattori 1975a, [Bibr B156][Bibr B164], Hattori and Piippo 1986.

***Gottschelia* Grolle**

*Gottscheliaschizopleura* (Spruce) Grolle. **MOLUCCAS**: Váňa and Piippo 1989b, Váňa 1991b, Schuster 2002, Long and Váňa 2007. Halmahera: **Sande Lacoste 1864** as *Jungermanniaflexicaulis* β *microphylla*. Ternate/Tidore: **Schiffner 1898** as Jamesoniellaflexicaulisvar.microphylla, 1900 as *Jamesoniellamicrophylla*, **Stephani 1901b** as *Jamesoniellamicrophylla*, Bonner 1966 as *Jamesoniellamicrophylla*, **Grolle 1968a**, Bizot and Pócs 1974, Miller et al. 1983. Seram: **Sande Lacoste 1864** as *Jungermanniaflexicaulis* β *microphylla*, **Schiffner 1898** as Jamesoniellaflexicaulisvar.microphylla, 1900 as *Jamesoniellamicrophylla*, **Stephani 1901b** as *Jamesoniellamicrophylla*, Bonner 1966 as *Jamesoniellamicrophylla*, **Grolle 1968a**, Miller et al. 1983, Bapna and Kachroo 2000b.

***Hattoriolejeunea* Mizut.**

*Hattoriolejeuneaakiyamae* Mizut. **MOLUCCAS**: Seram: **HOLOTYPE, Mizutani 1986a, Engel 1993, Crosby and Engel 2006, Akiyama 2009**.

***Herbertus* Gray**

*Herbertuslongifissus* Steph. **MOLUCCAS**: Seram: **Akiyama 1986, 2009**.

*Herbertuslongispinus* J.B.Jack et Steph. **MOLUCCAS**: Ambon: **Schiffner 1893b** as ‘*Herbertalongispina*’ [but represents *Herbertuspilifer*], **1898** as “*Herbertalongispina*’ [but citing only the type of *Herbertuspilifer*]. Seram: **Akiyama 1986, 2009**.

*Herbertuspilifer* (Steph.) H.A.Mill. **MOLUCCAS**: Ambon: **LECTOTYPE** of *Schismapilifera* [Miller 1965: 327], Stephani 1909c as *Schismapilifera*, **Miller 1965** as ‘*Herbertapilifera*’, Miller et al. 1983 as *Herbertuspilifer* and *Schismapiligerum*, **Piippo 1984a** as *Herbertuspilifer*, **So 2003b** as *Herbertuspilifer*, **Juslén 2006** as *Herbertuspilifer*.

*Herbertusramosus* (Steph.) H.A.Mill. **MOLUCCAS**: Seram: **Akiyama 1986, 2009** both as *Herbertusjavanicus*.

*Herbertussendtneri* (Nees) Lindb. **MOLUCCAS**: Seram: **LECTOTYPE** of *Schismadivaricatum* [Miller 1965: 325 as “type”], **Herzog 1921**, 1926a both as *Schismadivaricatum*, **Miller 1965** as ‘*Herbertadivaricata*’, Del Rosario 1975a as ‘*Herbertadivaricata*’, **Piippo 1984a** as *Herbertusarmitanus*, **Akiyama 1986, 2009** both as *Herbertusarmitanus*, **Gao 2003** as *Herbertusdivaricatus*, **So 2003b** as *Herbertusarmitanus*, **Juslén 2006** as *Herbertusarmitanus*.

***Heteroscyphus* Schiffn.**

*Heteroscyphusamboinensis* (Schiffn.) Schiffn. **MOLUCCAS**: Ambon: **LECTOTYPE** of *Chiloscyphusendlicherianus* δ *amboinensis* [to be designated], **Schiffner 1890** as *Chiloscyphusendlicherianus* δ *amboinensis*, **1898** as *Chiloscyphusamboinensis*, **Stephani 1907** as *Chiloscyphusamboinensis*, **Bonner 1963b** as Chiloscyphusdecurrensvar.amboinensis [error for *C.endlicherianus* δ *amboinensis*] and *Chiloscyphusamboinensis*, Miller et al. 1983 as *Chiloscyphusendlicherianus* δ *amboinensis*.

*Heteroscyphusargutus* (Reinw., Blume et Nees) Schiffn. **MOLUCCAS**: Seram: **Akiyama 1986, 2009**.

*Heteroscyphusaselliformis* (Reinw., Blume et Nees) Schiffn. **MOLUCCAS**: Ambon: **Schiffner 1893b, 1898** both as *Chiloscyphusaselliformis*, Stephani 1907 as *Chiloscyphusaselliformis*, Bonner 1963b as *Chiloscyphusaselliformis* and *Gamoscyphusaselliformis*, Grolle 1965 as *Chiloscyphusaselliformis*, Inoue 1965, Bonner 1966, 1976 as *Jungermanniaaselliformis*, Kitagawa 1979a as *Chiloscyphusaselliformis*, Miller et al. 1983, Piippo 1985, 1989b, Yamada and Hayashi 2003. Seram: **Akiyama 1986, 2009**.

Heteroscyphuscoalitus(Hook.)Schiffn.var.coalitus**MOLUCCAS**: Ambon: **Sande Lacoste 1864** as *Chiloscyphuscoalitus*, **Schiffner 1890, 1898** both as *Chiloscyphuscoalitus*, **Sydow 1894** as *Chiloscyphuscoalitus*, Inoue and Miller 1965 as *Heteroscyphus* ‘*coaltitus*’, Miller et al. 1983, Piippo 1985, 1989b, 1993b. Seram: **Akiyama 1986, 2009**.

*Heteroscyphussplendens* (Lehm. et Lindenb.) Grolle. **MOLUCCAS**: Halmahera: **Sande Lacoste 1864** as *Chiloscyphusdecurrens*, De Notaris 1874 as *Chiloscyphusdecurrens*, **Schiffner 1898** as *Chiloscyphusdecurrens*, Pócs 1971 as *Heteroscyphusdecurrens*, Miller et al. 1983 as *Heteroscyphusdecurrens*. Ambon: **Schiffner 1893b, 1898** both as *Chiloscyphusdecurrens*, Grolle 1965 as *Chiloscyphusdecurrens*, Pócs 1971 as *Heteroscyphusdecurrens*, Kitagawa 1979a as *Chiloscyphusdecurrens*, Miller et al. 1983 as *Heteroscyphusdecurrens*, Piippo 1985, 1989b, 1993b, Piippo and Tan 1992, Bapna and Kachroo 2000b as *Chiloscyphusdecurrens*, Yamada and Hayashi 2003, Hayashi and Yamada 2004. Seram: **Sande Lacoste 1864** as *Chiloscyphusdecurrens*, **Schiffner 1898** as *Chiloscyphusdecurrens*, **Herzog 1921** as *Chiloscyphusdecurrens*, Grolle 1965 as *Chiloscyphusdecurrens*, Pócs 1971 as *Heteroscyphusdecurrens*, Kitagawa 1979a as *Chiloscyphusdecurrens*, Miller et al. 1983 as *Heteroscyphusdecurrens*, Piippo 1985, **Akiyama 1986**, Piippo 1989b, 1993b, Piippo and Tan 1992, 1993b, Yamada and Hayashi 2003, Hayashi and Yamada 2004, **Akiyama 2009**.

*Heteroscyphuszollingeri* (Gottsche) Schiffn. **MOLUCCAS**: Seram: **Akiyama 1986, 2009**.

***Kurzia* G.Martens**

*Kurziageniculata* Mizut. **MOLUCCAS**: Seram: **Akiyama 1986, 2009**.

*Kurziagonyotricha* (Sande Lac.) Grolle. **MOLUCCAS**: Del Rosario 1971, 1975b both as *Microlepidoziagonyotricha*, Pócs et al. 2011. Seram: **Akiyama 1986, 2009**.

***Leiomitra* Lindb.**

*Leiomitrabreviseta* (Steph.) R.M.Schust. **MOLUCCAS**: Seram: **Akiyama 1986, 2009** both as *Trichocoleabreviseta*.

***Lejeunea* Lib.**

*Lejeuneaalbescens* (Steph.) Mizut. **MOLUCCAS**: Miller et al. 1983. Bacan: **Eifrig 1936** as *Taxilejeuneaalbescens*. Seram: **Mizutani 1986a**, **Akiyama 2009, Lee 2013**.

*Lejeuneaanisophylla* Mont. **MOLUCCAS**: Ambon: **Pócs 2010, Lee 2013**. Seram: **Mizutani 1986a** as *Lejeuneacatanduana*, **Akiyama 2009** as *Lejeuneacatanduana*.

*Lejeuneadiscreta* Lindenb. **MOLUCCAS**: Mizutani 1971, Tixier 1973a, 1979 both as *Hygrolejeuneadiscreta*, Mizutani 1975, 1977, Miller et al. 1983, Long and Grolle 1990, Parihar et al. 1994, Zhu and So 1999, Bapna and Kachroo 2000b, Chantanaorrapint et al. 2003, 2004, Alam and Srivastava 2009, Haerida 2009, Lee et al. 2010. Bacan: **Eifrig 1936** as *Hygrolejeuneadiscreta*.

*Lejeuneaexilis* (Reinw., Blume et Nees) Grolle. **MOLUCCAS**: Pócs et al. 2011. Ambon: **LECTOTYPE** of *Harpalejeuneaexigua* [Grolle & Reiner-Drehwald 1999: 41 as “holotype”], **Stephani 1913** as *Harpalejeuneaexigua*, **Grolle 1988** as *Lejeuneamicrostipula*, **Grolle and Reiner-Drehwald 1999** as *Microlejeuneamicrostipula*. Seram: **Mizutani 1986a**, **Akiyama 2009**.

*Lejeuneafissistipula* (Steph.) Steph. **MOLUCCAS**: Ambon: **LECTOTYPE** of *Eulejeuneafissistipula* [Bonner 1965: 138], **Stephani 1896b** as *Eulejeuneafissistipula*, 1915, **Schiffner 1898** as *Eulejeuneafissistipula*, **Bonner 1965** as *Eulejeuneafissistipula*, Miller et al. 1983 also as *Eulejeuneafissistipula*. Seram: **Mizutani 1986a**, **Akiyama 2009**.

Lejeuneaflava(Sw.)Neessubsp.orientalis R.M.Schust. **MOLUCCAS**: **LECTOTYPE** of *Jungermanniaindica* [to be designated]. Ternate/Tidore: **Reinwardt and Nees 1823** as *Jungermanniaindica*.

*Lejeunealumbricoides* (Nees) Nees. **MOLUCCAS**: Ambon: **LECTOTYPE** of *Taxilejeuneakarstenii* [to be designated], **Schiffner 1893b, 1898** as *Taxilejeunealumbricoides*, **Stephani 1914** as *Taxilejeuneakarstenii*, **Eifrig 1936** as *Taxilejeunealumbricoides*, Kamimura 1974, Lee et al. 2014. Seram: **Mizutani 1986a**, **Akiyama 2009, Lee 2013**.

*Lejeuneamicroloba* Taylor. **MOLUCCAS**: Seram: **Mizutani 1986a**, Zhu and Reiner-Drehwald 2004 as *Lejeuneachalmersii*, **Akiyama 2009, Lee 2013**.

*Lejeuneasordida* (Nees) Nees. **MOLUCCAS**: Mizutani 1977, Miller et al. 1983 also as *Hygrolejeuneaparkinsonii*, Pócs et al. 2011. Ambon: **LECTOTYPE** of *Hygrolejeuneaamboinensis* [to be designated], **Schiffner 1890, 1898** both as *Hygrolejeuneaamboinensis*, **Sydow 1894** as *Hygrolejeuneaamboinensis*, Stephani 1914 as *Hygrolejeuneaamboinensis*, **Eifrig 1936** as *Taxilejeunealaxiretis* and *Taxilejeuneasordida*, Miller et al. 1963, 1968 both as *Hygrolejeuneasordida*, Mizutani 1964 as *Taxilejeuneasordida*, Inoue and Miller 1965 as *Hygrolejeuneasordida*, Swanson and Miller 1969 as *Hygrolejeuneasordida*, Miller et al. 1983 also as *Hygrolejeuneasordida* and *Hygrolejeuneaamboinensis*, **Thiers 1986, Lee 2013**. Seram: **Mizutani 1986a**, **Akiyama 2009**.

*Lejeuneastenodentata* M.A.M.Renner et Pócs. **MOLUCCAS**: Mizutani 1977 as *Drepanolejeuneadentata*, Thouvenot 2015, Pócs et al. 2019a. Ambon: **LECTOTYPE** of *Drepanolejeuneadentata* [Bonner 1965: 82], **Stephani 1896b**, 1913 both as *Drepanolejeuneadentata*, **Schiffner 1898** as *Drepanolejeuneadentata*, **Bonner 1965** as *Drepanolejeuneadentata*, **Mizutani 1972** as *Drepanolejeuneadentata*, Pócs et al. 1995 as *Stenolejeuneadentata*, Schuster 2000b as *Stenolejeuneadentata*, **Renner et al. 2013**.

*Lejeuneaumbilicata* (Nees) Nees. **MOLUCCAS**: Ternate/Tidore: **Sande Lacoste 1856a, 1956b** both as *Omphalanthusumbilicatus*, **Schiffner 1898** as *Hygrolejeuneaumbilicata*.

***Lepicolea* Dumort.**

*Lepicolearara* (Steph.) Grolle. **MOLUCCAS**: Pócs et al. 2011. Seram: **Akiyama 1986, 2009**.

*Lepicoleayakusimensis* (S.Hatt.) S.Hatt. **MOLUCCAS**: Seram: **Akiyama 1986, 2009**.

***Lepidolejeunea* R.M.Schust.**

*Lepidolejeuneabidentula* (Steph.) R.M.Schust. **MOLUCCAS**: Geissler and Bischler 1989 as *Pycnolejeuneadecurvifolia*. Ambon: **LECTOTYPE** of *Hygrolejeuneadecurvifolia* [Mizutani 1978: 133], **Stephani 1896b**, 1910a both as *Hygrolejeuneadecurvifolia*, **Hoffmann 1935** as *Pycnolejeuneadecurvifolia*, Bonner 1966 as *Hygrolejeuneadecurvifolia*, **Mizutani 1978** as *Pycnolejeuneabadia*, Miller et al. 1983 as *Hygrolejeuneadecurvifolia*. Seram: **Mizutani 1986a**, **Akiyama 2009**.

***Lepidozia* (Dumort.) Dumort.**

*Lepidoziaborneensis* Steph. **MOLUCCAS**: Seram: **Akiyama 1986, 2009, Doei 1987b**.

*Lepidoziabrotheri* Steph. **MOLUCCAS**: Seram: **Doei 1987b, Akiyama 2009**.

*Lepidoziaceramensis* Herzog. **MOLUCCAS**: Seram: **LECTOTYPE** [to be designated], **Herzog 1926b**, Doei 1987b.

*Lepidoziacladorhiza* (Reinw., Blume et Nees) Nees. **MOLUCCAS**: Seram: **Akiyama 1986, 2009, Doei 1987b**.

*Lepidoziaferdinandii-muelleri* Steph. **MOLUCCAS**: Halmahera: Engel 1987. Seram: Herzog 1926a as *Lepidozia* ‘*Friderici Mülleri*’, **1926b** as *Lepidozia* ‘*Ferdinandi Mülleri*’, Grolle 1968b as *Lepidozia* ‘*fernandi-muelleri*’], Mizutani 1968b, 1974, Miller et al. 1983, Piippo 1984b, **Akiyama 1986, 2009** as *Lepidozia* ‘*ferdinandii-muelleri*’, Doei **1987b**, Enroth 1991, Schuster 2000a.

*Lepidoziagriseola* Herzog. **MOLUCCAS**: Seram: **LECTOTYPE** [to be designated], **Herzog 1926b**, Doei 1987b.

*Lepidoziahaskarliana* (Gottsche, Lindenb. et Nees) Steph. **MOLUCCAS**: Siregar et al. 2018. Seram: **Akiyama 1986, 2009** as *Lepidozia* ‘*hasskarliana*’, **Doei 1987b** as *Lepidozia* ‘*hasskarliana*’, von Konrat et al. 2014, Siregar and Pasaribu 2019.

*Lepidoziaintegrifolia* Doei. **MOLUCCAS**: Seram: **HOLOTYPE, Doei 1987a, 1987b, Engel 1993, Crosby and Engel 2006, Akiyama 2009**.

*Lepidozialongifolia* Steph. **MOLUCCAS**: Ambon: **LECTOTYPE** [here designated], **Stephani 1909a**, Miller et al. 1983.

*Lepidoziamassartiana* Schiffn. ex Steph. **MOLUCCAS**: Ambon: **Stephani 1909a**, Kitagawa 1973, Miller et al. 1983, **Geissler and Bischler 1985**.

*Lepidoziasubintegra* Lindenb. **MOLUCCAS**: Inoue and Miller 1965, Mizutani 1968a, 1976, Del Rosario 1975b, Miller et al. 1983, Bapna and Kachroo 2000a, Bakalin et al. 2021. Seram: **Akiyama 1986**, 2009 [misidentification of *Lepidoziasubtrichodes* fide Doei (1987b: 525).].

*Lepidoziasubtrichodes* Steph. **MOLUCCAS**: Seram: **Herzog 1926b, 2009, Doei 1987b**.

*Lepidoziatrichodes* (Reinw., Blume et Nees) Nees. **MOLUCCAS**: Ambon: **Schiffner 1893b, 1898**, Mizutani 1968a, 1974, Pócs 1968, Del Rosario 1975b, Kamimura 1975 as *Lepidozia* ‘*tricodes*’], Miller et al. 1983, Piippo 1984b, Doei 1987b, Siregar et al. 2018. Seram: **Akiyama 1986, 2009, Doei 1987b**.

***Leptolejeunea* (Spruce) Steph.**

*Leptolejeuneadentistipula* Steph. **MOLUCCAS**: Seram: **Mizutani 1986a**, **Akiyama 2009** as ‘Lepidolejeunea’ dentistipula.

*Leptolejeuneaelliptica* (Lehm. et Lindenb.) Besch. **MOLUCCAS**: Dey et al. 2009. Seram: **Mizutani 1986a**, **Akiyama 2009** as ‘Lepidolejeunea’ elliptica.

*Leptolejeuneaepiphylla* (Mitt.) Steph. **MOLUCCAS**: Bapna and Kachroo 2000b. Seram: **Mizutani 1986a**, **Akiyama 2009** as ‘Lepidolejeunea’ epiphylla.

*Leptolejeuneafoliicola* Steph. **MOLUCCAS**: Tixier 1974b, Parihar et al. 1994, Bapna and Kachroo 2000b. Seram: **Akiyama 2009** as ‘Lepidolejeunea’ foliicola.

*Leptolejeuneamaculata* (Mitt.) Schiffn. **MOLUCCAS**: Vanden Berghen 1977, Haerida 2009, Siregar et al. 2020a. Seram: **Mizutani 1986a**, **Akiyama 2009** as ‘Lepidolejeunea’ maculata.

*Leptolejeuneaschiffneri* (Steph. ex Schiffn.) Steph. **MOLUCCAS**: Tixier 1972 as *Leptolejeuneaschiffneri*, Mizutani 1975 as *Leptolejeuneaschiffneri*, Miller et al. 1983 as *Leptolejeuneaschiffneri*, **Geissler and Bischler 1985** as Leptolejeuneaschiffnerif.angustifolia, Bapna and Kachroo 2000b. Bacan: **Herzog 1942** as Leptolejeuneaschiffnerivar.genuina.

*Leptolejeuneavitrea* (Nees) Schiffn. **MOLUCCAS**: Jovet-Ast 1958, Jovet-Ast and Tixier 1962, Tixier 1970a, 1970b, 1971, 1973a, 1977, 1979, 1980, Miller et al. 1983, Pócs et al. 1994, Eggers 2006, Siregar et al. 2020a. Bacan: **Herzog 1942**.

***Lobatiriccardia* (Mizut. et S.Hatt.) Furuki**

*Lobatiriccardiacoronopus* (De Not.) Furuki. **MOLUCCAS**: Seram: **Furuki 1996** as *Lobatiriccardialobata*, Furuki 2006.

— subsp. coronopus. **MOLUCCAS**: Seram: Preussing et al. 2010.

***Lophocolea* (Dumort.) Dumort.**

*Lophocoleadeningeri* Herzog. **MOLUCCAS**: Buru: **LECTOTYPE** [here designated], **Herzog 1921**.

*Lophocoleakurzii* Sande Lac. **MOLUCCAS**: Seram: **Akiyama 1986, 2009**.

***Lopholejeunea* (Spruce) Steph.**

*Lopholejeuneaapplanata* (Reinw., Blume et Nees) Schiffn. **MOLUCCAS**: Ternate/Tidore: **Sande Lacoste 1864** as *Lejeunea* ‘*adplanata*’, **Schiffner 1898**. Ambon: **Sande Lacoste 1864** as *Lejeunea* ‘*adplanata*’, Miller et al. 1983. Seram: **Sande Lacoste 1864** as *Lejeunea* ‘*adplanata*’, **Schiffner 1898**, Miller et al. 1983.

*Lopholejeuneaeulopha* (Taylor) Schiffn. **MOLUCCAS**: Haerida et al. 2010, Siregar et al. 2020a. Halmahera: Verdoorn 1934a, **1934b**, Hattori 1951, Kamimura 1974, Miller et al. 1983. Ternate/Tidore: **Mizutani 1979**, Miller et al. 1983, **Zhu and Gradstein 2005**. Ambon: **Schiffner 1890** as *Lopholejeuneaeulopha*, **Sydow 1894**, Verdoorn 1934a, **1934b**, Hattori 1951, Kamimura 1974, Miller et al. 1983. Seram: **Mizutani 1979, 1986a**, Miller et al. 1983, **Akiyama 2009**.

*Lopholejeuneahorticola* Schiffn. **MOLUCCAS**: Haerida et al. 2010, Siregar et al. 2014, Siregar 2015. Seram: **Mizutani 1986a**, **Akiyama 2009**.

*Lopholejeuneanigricans* (Lindenb.) Schiffn. **MOLUCCAS**: Mizutani 1994, Haerida et al. 2010, Siregar et al. 2014, Siregar et al. 2020a. Ternate/Tidore: **Verdoorn 1934b**. Seram: **Mizutani 1986a**, **Akiyama 2009** also as *Lopholejeuneajavanica*.

*Lopholejeuneasubfusca* (Nees) Schiffn. **MOLUCCAS**: Mizutani 1994, Haerida 2009, Haerida et al. 2010, Siregar et al. 2014, 2020a, Siregar 2015, Rosyanti et al. 2018. Ambon: **Schiffner 1893b** as *Lejeunea* ‘*Sagraeana*’, **1898** as Lopholejeuneasagranavar.subfusca, **Stephani 1912b** as *Lopholejeuneapyriflora*, Verdoorn 1934a, **1934b**, Hattori 1951, Jovet-Ast 1958, Inoue and Miller 1965, Tixier 1966, 1967a, 1967b, Pócs et al. 1967, Swanson and Miller 1969, Miller et al. 1983 also as *Lopholejeuneapyriflora* and *Lopholejeuneasagrana*, **Geissler and Bischler 1985** as *Lopholejeuneapyriflora*, Bapna and Kachroo 2000b, Gradstein et al. 2002, **Zhu and Gradstein 2005**. Banda: **Verdoorn 1934b**, Jovet-Ast 1958, Tixier 1966, 1967a, Swanson and Miller 1969. Seram: **Mizutani 1986a**, Zhu and Gradstein 2005, **Akiyama 2009**.

*Lopholejeuneawiltensii* Steph. **MOLUCCAS**: Haerida et al. 2010, Siregar 2015. Halmahera: **Umagap 2019**. Ternate/Tidore: **Mizutani 1979**, Zhu and Gradstein 2005. Seram: **Mizutani 1979, Zhu and Gradstein 2005**, Siregar et al. 2014, Siregar 2015.

***Marchantia* L.**

*Marchantiaemarginata* Reinw., Blume et Nees. **MOLUCCAS**: Siregar et al. 2013, Siregar 2015, Ginting and Batubara 2019, Siregar et al. 2020b, Ruklani and Rubasinghe 2022. Ternate/Tidore: Bischler and Piippo 1991. Seram: Bischler and Piippo 1991.

— subsp. emarginata. **MOLUCCAS**: **Bischler 1989**, Singh and Singh 2013. Ternate/Tidore: **Bischler-Causse 1989**. Ambon: **LECTOTYPE** of *Marchantiaamboinensis* [Bischler-Causse 1989: 183 as “type”, second-step lectotypification needed], **Montagne 1838, 1856** both as *Marchantiaamboinensis*, **Gottsche et al. 1846** as *Marchantiaamboinensis*, **Schiffner 1898** as *Marchantiaamboinensis*, **Stephani 1899a** as *Marchantiaamboinensis*, Miller et al. 1983 as *Marchantiaamboinensis*, **Bischler-Causse 1989**, Bischler and Piippo 1991. Seram: **Bischler-Causse 1989**.

*Marchantiageminata* Reinw., Blume et Nees. **MOLUCCAS**: Halmahera: **Umagap 2019**.

*Marchantiapolymorpha* L. **MOLUCCAS**: Ambon: **Sopacua et al. 2020** [Saparua I].

*Marchantiarubribarba* Steph. **MOLUCCAS**: Ambon: **Bischler-Causse 1989**, Bischler and Piippo 1991.

***Mastigophora* Nees**

*Mastigophoradiclados* (Brid. ex F.Weber) Nees. **MOLUCCAS**: Buru: **Herzog 1921**. Halmahera: **LECTOTYPE** of *Sendtneravrieseana* [to be designated], **Sande Lacoste 1864** as *Sendtneradiclados* β *calcarata* and *Sendtneravrieseana*, **Schiffner 1898** also as *Mastigophoravrieseana*, Stephani 1909c as *Mastigophoravrieseana*, Inoue and Miller 1965, Miller et al. 1983. Ternate/Tidore: **Sande Lacoste 1864** as *Sendtneradiclados* α *scorpioides*, **Schiffner 1898** as Mastigophoradicladosvar.scorpioides, Inoue and Miller 1965, Miller et al. 1983. Ambon: **Schiffner 1898**, Inoue and Miller 1965, Miller et al. 1983. Seram: **Herzog 1921**, **Akiyama 1986, 2009**.

— var. borneensis (De Not.) Schiffn. **MOLUCCAS**: Ambon: **Schiffner 1893b, 1898**.

— var. diclados. **MOLUCCAS**: Ambon: **Schiffner 1893b** as *Mastigophoradiclados*.

***Metalejeunea* Grolle**

*Metalejeuneacucullata* (Reinw., Blume et Nees) Grolle. **MOLUCCAS**: **Grolle 1995**. Ambon: **SYNTYPE** of *Microlejeuneaparallela* [lectotype to be designated], **Schiffner 1890** as *Microlejeuneaparallela*, 1898 as *Eulejeuneaparallela*, **Sydow 1894** as *Microlejeuneaparallela*, Stephani 1915 as *Microlejeuneaparallela*. Seram: **Mizutani 1986a** as *Lejeuneacucullata*, **Akiyama 2009** as *Lejeuneacucullata*.

***Metzgeria* Raddi**

*Metzgeriaciliata* Raddi. **MOLUCCAS**: Halmahera: **Umagap 2019** as *Metzgeriadecipiens*.

*Metzgeriafoliicola* Schiffn. **MOLUCCAS**: Ternate/Tidore: **So 2003c**. Seram: **Akiyama 1986, 2009**.

*Metzgeriafurcata* (L.) Corda. **MOLUCCAS**: Halmahera: **Umagap 2019**.

MetzgerialeptoneuraSprucevar.leptoneura**MOLUCCAS**: Engel 1976, Schuster 1983. Buru: **So 2003c**. Ambon: **LECTOTYPE** of *Metzgeriahamatiformis* [to be designated], **Schiffner 1893b, 1898** both as *Metzgeriahamatiformis*, **Stephani 1899d** as *Metzgeriahamatiformis*, **Kuwahara 1966** as *Metzgeriahamata*, 1984, Schuster 1992, **Engel 1978**, Miller et al. 1983, **So 2003c, Costa 2008**. Seram: **Akiyama 1986, 2009**.

***Microlejeunea* (Spruce) Steph.**

*Microlejeuneafilicuspis* (Steph.) Heinrichs, Schäf.-Verw., Pócs et S.Dong. **MOLUCCAS**: **Grolle 1979a** as *Harpalejeuneafilicuspis*, Miller et al. 1983 as *Drepanolejeuneafilicuspis*. Ambon: **Grolle 1979a** as *Harpalejeuneafilicuspis*.

***Mnioloma* Herzog**

*Mniolomafuscum* (Lehm.) R.M.Schust. **MOLUCCAS**: Seram: **Akiyama 1986, 2009** both as *Calypogeiafusca*.

***Neolepidozia* Fulford et J.Taylor**

*Neolepidoziacuneifolia* (Steph.) Fulford et J.Taylor. **MOLUCCAS**: Ambon: **Engel and Smith Merrill 2004** as *Telaraneacuneifolia*, von Konrat et al. 2014.

*Neolepidoziapapulosa* (Steph.) Fulford et J.Taylor. **MOLUCCAS**: Seram: **Akiyama 1986, 2009** both as *Lepidoziapapulosa*, **Doei 1987b** as *Lepidoziapapulosa*.

*Neolepidoziawallichiana* (Gottsche) Fulford et J.Taylor. **MOLUCCAS**: Ambon: **Sande Lacoste 1864** as *Lepidoziawallichiana*, De Notaris 1874 as *Lepidoziawallichiana*, **Schiffner 1893b, 1898** both as *Lepidoziawallichiana*, Hattori and Mizutani 1958 as *Lepidoziawallichiana*, Inoue and Miller 1965 as *Lepidoziawallichiana*, Swanson and Miller 1969, Mizutani 1974, 1976 both as *Lepidoziawallichiana*, Del Rosario 1975b as *Lepidoziawallichiana*, Kamimura 1975 as *Lepidoziawallichiana*, Miller et al. 1983, Piippo 1984b as *Lepidoziawallichiana*, Doei 1987b as *Lepidoziawallichiana*, Sharma and Srivastava 1993 as *Lepidoziawallichiana*, Bapna and Kachroo 2000a as *Lepidoziawallichiana*, Engel and Smith Merrill 2004 as *Telaraneawallichiana*. Seram: **Doei 1987b** as *Lepidoziawallichiana*, **Akiyama 2009** as *Lepidoziawallichiana*.

***Pallavicinia* Gray**

*Pallaviciniaambigua* (Mitt.) Steph. **MOLUCCAS**: Seram: **Grolle and Piippo 1986**, Koponen et al. 2000, Furuki 2002.

*Pallavicinialyellii* (Hook.) Gray. **MOLUCCAS**: Inoue and Miller 1965, Swanson and Miller 1969, Miller et al. 1983, Bapna and Kachroo 2000b, Manju et al. 2015. Halmahera: **Umagap 2019**.

***Plagiochila* (Dumort.) Dumort.**

*Plagiochilaabietina* (Nees) Mont. et Nees. **MOLUCCAS**: Ambon: **Stephani 1903b**, Bonner 1962a, Miller et al. 1983.

*Plagiochilaakiyamae* Inoue. **MOLUCCAS**: Seram: **HOLOTYPE, Inoue 1986, Engel 1993, Grolle and So 1998, Grolle and So 1999, So and Grolle 2000, So 2001b, Crosby and Engel 2006, Akiyama 2009**, Singh and Rawat 2020.

*Plagiochilaamboynensis* Taylor. **MOLUCCAS**: **Geissler and Bischler 1989** [type, Amboina], **So and Grolle 2000** [type, Amboina]. Ambon: **LECTOTYPE** [So and Grolle 2000: 201 as “**holotype**”], T**aylor 1846, Gottsche et al. 1847** as *Plagiochila* ‘*amboinensis*’, **Schiffner 1898** as *Plagiochila* ‘*amboinensis*’, **Stephani 1904a** as *Plagiochila* ‘*amboinensis*’, **Bonner 1962a, Inoue 1984, So 2001c**, Schuster 2021.

*Plagiochilaarbuscula* (Brid. ex Lehm. et Lindenb.) Lindenb. **MOLUCCAS**: Halmahera: **Sande Lacoste 1864** as *Plagiochilabelangeriana*, **Schiffner 1898** as *Plagiochilabelangeriana*, Miller et al. 1983. Ternate/Tidore: **Sande Lacoste 1864** as *Plagiochilabelangeriana*, Miller et al. 1983. Ambon: **Sande Lacoste 1864** as *Plagiochilabelangeriana* [Saparoea I], **Schiffner 1893b, 1898** both as *Plagiochilabelangeriana*, Miller et al. 1983. Seram: **Inoue 1986, So 2001b, Akiyama 2009**.

*Plagiochilabantamensis* (Reinw., Blume et Nees) Mont. **MOLUCCAS**: Seram: **Inoue 1986, Akiyama 2009**.

*Plagiochilabicornuta* Steph. **MOLUCCAS**: Pócs et al. 2011. Ambon: **Stephani 1903a**, Bonner 1962a, Inoue 1981, Miller et al. 1983, **So 2000a, 2001a**, Schuster 2021. Seram: So 2000a, 2001a.

*Plagiochilablepharophora* (Nees) Lindenb. **MOLUCCAS**: Ternate/Tidore: **Schiffner 1898**, 1900, Stephani 1903c, Miller et al. 1983.

*Plagiochilachauviniana* Mont. **MOLUCCAS**: Seram: **Inoue 1986** as *Plagiochilanovae-guineae*, **Akiyama 2009** as *Plagiochilanovae-guineae*.

*Plagiochilafrondescens* (Nees) Lindenb. **MOLUCCAS**: Ambon: **Sande Lacoste 1864** as *Plagiochilafrondescens* γ *rigida*, **Schiffner 1893b, 1898** 1900 all as Plagiochilafrondescensvar.tenerrima, **1898** as *Plagiochilafrondescens* γ *rigida*, **Stephani 1903a**, Swanson and Miller 1969, Miller et al. 1983, Piippo 1989a. Seram: **Inoue 1986, Akiyama 2009**.

*Plagiochilagymnoclada* Sande Lac. **MOLUCCAS**: Singh Deo and Singh 2014. Buru: **Herzog 1921, Carl 1931b, Inoue 1984**, Inoue 1989. Ternate/Tidore: **Sande Lacoste 1864**, **Schiffner 1898**, 1900, Stephani 1903b. Seram: **Inoue 1986** as *Plagiochilapseudaberrans*, **Akiyama 2009** as *Plagiochilapseudaberrans*.

*Plagiochilahampeana* Gottsche. **MOLUCCAS**: Seram: **Inoue 1986** as *Plagiochilagedeana*, So and Grolle 2001, **Akiyama 2009** as *Plagiochilagedeana*.

*Plagiochilajunghuhniana* Sande Lac. **MOLUCCAS**: Ambon: **So 2000b, 2001b**.

*Plagiochilakuhliana* Sande Lac. **MOLUCCAS**: Seram: **Sande Lacoste 1864**, **Schiffner 1898**, 1900, **Stephani 1903d**, Bonner 1962a, **Geissler and Bischler 1989**.

*Plagiochilakurzii* Steph. **MOLUCCAS**: Seram: **Sande Lacoste 1864** as Plagiochilabantamensisvar.denticulata, **Schiffner 1898** as Plagiochilabantamensisvar.denticulata, Bonner 1962a as Plagiochilabantamensisvar.denticulata.

*Plagiochilamassalongoana* Schiffn. **MOLUCCAS**: Seram: **Inoue 1986, Akiyama 2009**.

*Plagiochilapulvinata* Steph. **MOLUCCAS**: **So and Grolle 1999**. Seram: **HOLOTYPE** of *Plagiochilaceramica*, **Inoue 1986** as *Plagiochilaceramica*, **Engel 1993** as *Plagiochilaceramica*, **So and Grolle 2000, Crosby and Engel 2006** as *Plagiochilaceramica*, **Akiyama 2009** as *Plagiochilaceramica*.

*Plagiochilarenitens* (Nees) Lindenb. **MOLUCCAS**: ?Halmahera: Schiffner 1900, Stephani 1903b. Seram: **Inoue 1984, 1986**, 1989, Piippo 1989a, **Akiyama 2009**, Schuster 2021. Note: we are not aware of any first hand report from Halmahera.

*Plagiochilasalacensis* Gottsche. **MOLUCCAS**: Seram: **Inoue 1986, Akiyama 2009**.

*Plagiochilasandei* Dozy. **MOLUCCAS**: **So and Grolle 1999**. Ternate/Tidore: Schiffner and Stephani 1901 as Plagiochilablepharophoravar.major. Ambon: **Sande Lacoste 1864** [Saparoea I], **Schiffner 1898**, 1900 [both Saparoea I], Stephani 1903d [Saparoea I], **Grolle 1968b** as *Plagiochilaseemannii*, Miller et al. 1983 as *Plagiochilaseemannii*, Inoue 1986 as *Plagiochilaseemannii*, Piippo 1989a as *Plagiochilaseemannii*. Seram: **Sande Lacoste 1864**, Stephani 1903d, **Inoue 1986** also as *Plagiochilaseemannii*, 1989, Müller and Schäfer-Verwimp 1999, **Akiyama 2009** also as *Plagiochilaseemannii*.

*Plagiochilasciophila* Nees. **MOLUCCAS**: ?Ambon: Pócs 1971 as *Plagiochilaacanthophylla*. Seram: **Inoue 1986** also as *Plagiochiladecidua*, 1989, **Akiyama 2009** also as *Plagiochiladecidua*. Note: we aree not aware of any first hand report from Ambon.

*Plagiochilateysmannii* Sande Lac. **MOLUCCAS**: **So and Grolle 1999**. Bacan: **LECTOTYPE** of *Plagiochilabatjanensis* [Inoue 1984: 90 as “type”], **Herzog 1938** as *Plagiochilabatjanensis*, **Bonner 1962a** as *Plagiochilabatjanensis*, **Inoue 1984, Geissler and Bischler 1989** as *Plagiochilabatjanensis*, Piippo 1989a, **So and Grolle 2000**. Ambon: **Schiffner 1893b, 1898** both as *Plagiochilasemialata*. Seram: **Inoue 1986**, 1989, **Akiyama 2009** as *Plagiochila* ‘*teysmanii*’.

*Plagiochilatrabeculata* Steph. **MOLUCCAS**: Seram: **So 2001b**.

***Pleurozia* Dumort.**

*Pleuroziaconchifolia* (Hook. et Arn.) Austin. **MOLUCCAS**: Pócs et al. 2011. Ambon: **Schiffner 1898***Pleurozia* ‘*conchaefolia*’, Miller et al. 1983.

*Pleuroziagigantea* (F.Weber) Lindb. **MOLUCCAS**: Ambon: **Schiffner 1893b, 1898**, Horikawa 1935, Pócs et al. 1967, Tixier 1973b, 1974b, Bizot and Pócs 1974, Miller et al. 1983, **Thiers 1993**. Seram: **Akiyama 1986, 2009**.

***Plicanthus* R.M.Schust.**

*Plicanthushirtellus* (F.Weber) R.M.Schust. **MOLUCCAS**: Seram: **LECTOTYPE** of *Chandonanthusgracilis* [here designated], **Herzog 1921** as *Chandonanthushirtellus*, 1926a as *Chandonanthusgracilis*, **Herzog 1926b, 1926b** both as *Chandonanthushirtellus*, **Bonner 1963b** as *Chandonanthusgracilis*, **Akiyama 1986, 2009** both as *Chandonanthushirtellus*, **Váňa et al. 2013**.

***Podomitrium* Mitt.**

*Podomitriummalaccense* (Steph.) Campb. **MOLUCCAS**: Ambon: Troll 1930 as *Hymenophytonmalaccense*, Grolle 1966a, Schuster 1969 as *Podomitrium* ‘*mallacense*’, Miller et al. 1983, **Grolle and Piippo 1986**. Seram: **Akiyama 1986, 2009**.

***Porella* L.**

Porellaacutifolia(Lehm. et Lindenb.)Trevis.var.acutifolia**MOLUCCAS**: Halmahera: **Sande Lacoste 1864** as *Madothecaacutifolia*, **Schiffner 1898** as *Madothecaacutifolia*, **Hattori 1970** as Porellaacutifoliavar.subligulifera.

*Porellageheebii* (Steph.) S.Hatt. **MOLUCCAS**: Seram: **Bi et al. 2019**.

*Porellajavanica* (Gottsche) Inoue. **MOLUCCAS**: Hattori 1978, Miller et al. 1983. Buru: **LECTOTYPE** of *Madothecacrenilobula* [here designated], **Herzog 1921**, 1926a both as *Madothecacrenilobula*.

***Psiloclada* Mitt.**

PsilocladaclandestinaMitt.subsp.clandestina**MOLUCCAS**: Ambon: **LECTOTYPE** of *Psilocladaunguligera* [to be designated], **Schiffner 1893a**, 1893b, **1898** all as *Psilocladaunguligera*, **Stephani 1909a**, Herzog 1953, Arnell 1955, **Fulford and Taylor 1959** as *Psilocladaunguligera*, Arnell 1963, Mizutani 1974, 1976, Schuster 1980, Hürlimann 1983, Miller et al. 1983. Seram: **Akiyama 1986, 2009**.

— subsp. melanesica R.M.Schust. ?**MOLUCCAS**: Ambon: Miller 1985, Schuster 2000a with a “?”], Engel and Glenny 2008. Note: we are not aware of any positively identified specimen of this subspecies from **Moluccas**.

***Ptychanthus* Nees**

Ptychanthusstriatus(Lehm. et Lindenb.)Neesvar.striatus. **MOLUCCAS**: Mizutani 1977, Miller et al. 1983 as *Ptychanthusmoluccensis*, Haerida et al. 2010, Siregar et al. 2014, Siregar 2015, Siregar et al. 2020a. Buru: **Herzog 1921**, 1926a both as *Ptychanthusmoluccensis*. Halmahera: **Sande Lacoste 1864** as *Ptychanthusjavanicus*, **Schiffner 1898** as *Ptychanthusjavanicus*, **Verdoorn** 1934a, **1934b, Umagap 2019**. Ambon: **LECTOTYPE** of *Ptychanthussquarrosus* [to be designated], **Lehmann 1844** as *Ptychanthussquarrosus*, **Gottsche et al. 1845a** as *Ptychanthussquarrosus*, **Montagne 1856** as *Ptychanthussquarrosus*, **Stephani 1890a, Schiffner 1898**, Verdoorn 1933 as *Ptychanthusmoluccensis*, 1934a, Mizutani 1961, Tixier 1971, Kamimura 1974, Miller et al. 1983, Bapna and Kachroo 2000b. Seram: **SYNTYPE** of *Ptychanthusmoluccensis* [**lectotype** to be designated], **Sande Lacoste 1864** as *Ptychanthusmoluccensis*, **Schiffner 1898** as *Ptychanthusmoluccensis*, **Verdoorn** 1933 as *Ptychanthusmoluccensis*, **1934b**, Miller et al. 1983 as *Ptychanthusstriatus*, **Mizutani 1986a**, **Akiyama 2009**.

***Pycnolejeunea* (Spruce) Schiffn.**

*Pycnolejeuneacavistipula* (Steph.) Mizut. **MOLUCCAS**: Ambon: **LECTOTYPE** of *Strepsilejeuneacavistipula* [Mizutani 1972: 161 as “type”], **Stephani 1896b** as *Strepsilejeuneacavistipula*, 1913 as *Trachylejeuneacavistipula*, **Schiffner 1898** as *Strepsilejeuneacavistipula*, **Mizutani 1972, Thiers 1986**.

*Pycnolejeuneaconnivens* Schiffn. ex P.Syd. **MOLUCCAS**: Ambon: **LECTOTYPE** of *Pycnolejeuneaconnivens* [to be designated], **Schiffner 1890** as *Pycnolejeuneaconnivens*, **Sydow 1894**.

***Radula* Dumort.**

*Radulaanceps* Sande Lac. **MOLUCCAS**: Seram: **Akiyama 1986, 2009**, Yamada 1989, Bapna and Kachroo 2000b.

*Raduladensifolia* Castle. **MOLUCCAS**: Seram: **Akiyama 1986, 2009**, Yamada and Piippo 1989, Yamada 1989, Yamada 2002.

*Radulaformosa* (C.F.W.Meissn. ex Spreng.) Nees. **MOLUCCAS**: Ambon: **LECTOTYPE** of *Radulapycnolejeuneoides* [Castle 1950: 267; second-step lectotypification needed], **Schiffner 1893b, 1898** both as *Radulapycnolejeuneoides*, Stephani 1910b also as *Radulapycnolejeuneoides*, Jovet-Ast 1949, **Castle 1950**, 1968, Steere 1953, Inoue and Miller 1965, Grolle 1966a, **Kitagawa 1979a** as Radulaformosavar.pycnolejeuneoides, Miller et al. 1983 also as *Radulapycnolejeuneoides*, **So 2006**. ?Seram: Yamada 1979, 1989, Miller et al. 1983, Siregar 2015, Ginting and Batubara 2019. Note: we are not aware of any first hand report from Seram.

*Radulajavanica* Gottsche. **MOLUCCAS**: Buru: New to Moluccas: Buru, E hoe, 1921.09, *Toxopens 299a*, det. H. Castle (BO4137]. Seram: **LECTOTYPE** of *Radulaceramensis* [to be designated], **LECTOTYPE** of *Radulaovalifolia* [Castle 1966: 68; second-step lectotypification needed)], **Stephani 1884** as *Radulaovalifolia* and *Radulaceramensis*, **Schiffner 1898** as *Radulaceramensis* and *Radulaovalifolia*, **Stephani** 1910b, **1924** both as *Radulaceramensis*, **Castle 1966** as *Radulawallichiana*, Miller et al. 1983 as *Radulaceramensis*, **Akiyama 1986, 2009, Yamada 1989**.

*Radulamultiflora* Gottsche ex Schiffn. **MOLUCCAS**: Seram: **Akiyama 1986**.

*Radulanymannii* Steph. **MOLUCCAS**: Ambon: Swanson and Miller 1969, Miller et al. 1983. Seram: **Akiyama 1986, 2009** as *Radula* ‘*nymanii*’, Yamada 1989 as *Radula* ‘*nymanii*’.

*Radulareflexa* Nees et Mont. **MOLUCCAS**: Ambon: **LECTOTYPE** [Castle 1965: 374], **Montagne 1843**, 1846, **1856, Gottsche et al. 1845a, Schiffner 1898, Castle 1965, 1968, Yamada 1976, 1979**, 2002, Miller et al. 1983, **So 2005, 2006**. Seram: **LECTOTYPE** of *Radulasubsimilis* [Castle 1965: 376 as “type”], **Sande Lacoste 1864, Stephani 1884** as *Radulasubsimilis*, **Schiffner 1898** also as *Radulasubsimilis*, Stephani 1910b as *Radulasubsimilis*, **Castle 1965, 1968** both as *Radulasubsimilis*, Yamada 1979 as *Radulasubsimilis*, 1987, Miller et al. 1983 also as *Radulasubsimilis*, Yamada and Piippo 1989, Streimann 1991 as *Radulasubsimilis*, Piippo et al. 2002, **So 2006**, Siregar 2015.

*Radulasumatrana* Steph. **MOLUCCAS**: Seram: **Akiyama 1986, 2009**, Yamada 1989.

*Radulatjibodensis* K.I.Goebel. **MOLUCCAS**: Seram: **Akiyama 1986, 2009**, Yamada 1989, Zhu 1999, Zhu and So 2001.

*Radulavan-zantenii* K.Yamada. **MOLUCCAS**: Seram: **Akiyama 1986, 2009** as *Radula* ‘*vanzantenii*’, Yamada and Piippo 1989.

*Radulavrieseana* Sande Lac. **MOLUCCAS**: Siregar 2015. Obi: **Yamada 1979**. Banda: **Sande Lacoste 1864**, **Schiffner 1898**. Seram: **LECTOTYPE** [Castle 1965: 372], **Sande Lacoste 1864**, **Schiffner 1898**, Stephani 1910b as *Radula* ‘*vriesei*’, **Castle 1965, 1968** both as *Radula* ‘*Vriesei*’, **Yamada 1974** as *Radula* ‘*vriesei*’, **1979, Akiyama 1986, 1989, 2009, Yamada and Piippo 1989**, Bapna and Kachroo 2000b, Siregar 2015.

***Riccardia* Gray**

*Riccardiaalbomarginata* (Steph.) Schiffn. **MOLUCCAS**: Ambon: **LECTOTYPE** of *Aneuraalbomarginata* [Bonner 1962b: 85, cf. also Furuki 1995: 112 as “holotype”], **Stephani 1893a**, 1893b, **1899c** all as *Aneura* ‘*albo-marginata*’, **Schiffner 1898, Bonner 1962b** as *Aneuraalbomarginata*, Grolle 1967, 1968b, Miller et al. 1983 also as *Aneuraalbomarginata*, **Furuki 1995**, Thouvenot et al. 2018.

*Riccardiaaspera* (Steph.) Grolle. **MOLUCCAS**: Seram: **Furuki 1998**.

*Riccardiakarstenii* (Steph.) Schiffn. **MOLUCCAS**: Ambon: **LECTOTYPE** of *Aneurakarstenii* [Bonner 1962b: 114], **Stephani 1893a**, 1893b, **1899c** all as *Aneurakarstenii*, **Schiffner 1898, Bonner 1962b** as *Aneurakarstenii*.

***Riccia* L.**

*Ricciaamboinensis* Schiffn. **MOLUCCAS**: Ambon: **LECTOTYPE** [to be designated], **Schiffner 1890, 1898, Sydow 1894, Stephani 1898**, Campbell 1915 as *Riccia* ‘*amboiniana*’.

*Ricciabillardierei* Mont. et Nees. **MOLUCCAS**: Bapna and Kachroo 2000b, Chaudhary et al. 2006. Halmahera: **Meijer 1958**, Jovet-Ast 2000, 2003. Banda: **Jovet-Ast 2003**.

*Ricciajunghuhniana* Nees et Lindenb. **MOLUCCAS**: Ambon: **Xiang et al. 2022**.

***Saccogynidium* Grolle**

*Saccogynidiummuricellum* (De Not.) Grolle. **MOLUCCAS**: Miller et al. 1983, Enroth 1991. Bacan: **Grolle and Schultze-Motel 1972**. ?Ambon: Piippo 1985, 1989b, Gao et al. 2001. Seram: **Akiyama 1986, 2009**. Note: we are not aware of any first hand report from Ambon.

*Saccogynidiumrigidulum* (Nees) Grolle. **MOLUCCAS**: Inoue and Miller 1965, **Grolle and Schultze-Motel 1972**, Miller et al. 1983, Enroth 1991. Ambon: **LECTOTYPE** of *Chiloscyphusgranulatus* [to be designated], **Schiffner 1893b, 1898** as *Chiloscyphusgranulatus*, **Stephani 1908a** as *Saccogynarigidula*, Hattori 1951 as *Saccogynarigidula*, **Bonner 1963b** as *Chiloscyphusgranulatus*, Inoue and Miller 1965, **Grolle 1968b** as *Saccogynidiumjugatum*, Miller et al. 1983 also as *Chiloscyphusgranulatus*, Piippo 1985, 1989b, Gao et al. 2001.

***Sandeothallus* R.M.Schust.**

*Sandeothallusradiculosus* (Schiffn.) R.M.Schust. **MOLUCCAS**: Ambon: **Akiyama 2009**. Seram: **Akiyama 1986**.

***Schiffneria* Steph.**

*Schiffneriahyalina* Steph. **MOLUCCAS**: Engel 1987, Long and Grolle 1990, Piippo and Tan 1992, Váňa 1993, O’Shea et al. 1997, Bapna and Kachroo 2000a, Schuster 2002. Halmahera: Bacan: **LECTOTYPE** [Grolle and Piippo 1984: 305 ad “holotype”], **Stephani 1894b, 1908a, Schiffner 1898, Kitagawa 1973, Grolle and Piippo 1984, Asthana et al. 1994, Váňa et al. 2014**.

***Schiffneriolejeunea* Verd.**

*Schiffneriolejeuneacumingiana* (Mont.) Gradst. **MOLUCCAS**: Ambon: **Verdoorn** 1934a, **1934b** both as *Ptychocoleuscumingianus*, Tixier 1966, 1967b, 1972 all as *Ptychocoleuscumingianus*, Miller 1968 as *Ptychocoleuscumingianus*, Swanson and Miller 1969 as *Ptychocoleuscumingianus*, Kachroo 1970 as *Ptychocoleuscumingianus*, Miller et al. 1983, Bapna and Kachroo 2000b. Seram: **Verdoorn** 1934a, **1934b** both as *Ptychocoleuscumingianus*, Miller 1968 as *Ptychocoleuscumingianus*, Swanson and Miller 1969 as *Ptychocoleuscumingianus*, **Gradstein and Terken 1981**, Miller et al. 1983.

*Schiffneriolejeuneapulopenangensis* (Gottsche) Gradst. **MOLUCCAS**: Bapna and Kachroo 2000b, Siregar et al. 2020a. Ambon: **ORIGINAL MATERIAL** of *Acrolejeuneadensifolia*, **Schiffner 1890** as *Acrolejeuneadensifolia*, **1898** as *Acrolejeuneapulopenangensis*, **Sydow 1894** as *Acrolejeuneadensifolia*, Stephani 1912b as *Ptychocoleusdensifolius*, **Verdoorn 1934b** as *Ptychocoleuspulopenangensis*, **Bonner 1962b** as *Acrolejeuneadensifolia*, Mizutani 1964 as *Ptychocoleuspulopenangensis*, Miller et al. 1983 also as *Acrolejeuneadensifolia*, Hürlimann 1989.

*Schiffneriolejeuneatumida* (Nees) Gradst. **MOLUCCAS**: Ambon: **Gradstein and Terken 1981**. Seram: **Mizutani 1986a**, **Akiyama 2009**.

— var. haskarliana (Gottsche) Gradst. et Terken. **MOLUCCAS**: Mizutani 1977, 1978 both as *Schiffneriolejeunea* ‘*hasskarliana*’. Ambon: **Schiffner 1893b** as *Lejeuneahaskarliana*, **1898** as *Acrolejeuneahaskarliana*, **Verdoorn** 1934a, **1934b** as *Ptychocoleus* ‘*Hasskarlianus*’, Amakawa 1960 as *Ptychocoleushaskarlianus*, Miller et al. 1983 as *Acrolejeuneahaskarliana* and *Schiffneriolejeuneahaskarliana*.

***Schistochila* Dumort.**

*Schistochilaacuminata* Steph. **MOLUCCAS**: Seram: **LECTOTYPE** of *Schistochilapurpurascens* [here designated, cf. So 2003a: 80 as “**holotype**”)], **Herzog** 1926a, **1926b** both as *Schistochilapurpurascens*, Grolle 1966b as *Schistochilapurpurascens*, Hässel 1976 as *Schistochilapurpurascens*, **So 2003a**.

*Schistochilaaligera* (Nees et Blume) J.B.Jack et Steph. **MOLUCCAS**: Pócs 1971, Tixier 1972. Buru: **Herzog 1921**, 1926a also as *Schistochilagraeffeana*, Miller et al. 1983 as *Schistochilastergraeffeanus* and *Schistochilagraeffeana*, **So 2003a**. Bacan: **Buch 1939**. ?Halmahera: Miller et al. 1983 as *Schistochilasteraliger*. Ambon: **ORIGINAL MATERIAL** of Schistochilaphilippinensisvar.transiens, **Schiffner 1893b** as ‘Schistocheila’ aligera, **1898**, Stephani 1896a as ‘*Schistocheila’ philippinensis*, 1909c, **Buch 1939** also as *Schistochilaphilippinensis*, Bonner 1966 as *Gottscheaaligera*, Grolle 1966b also as *Schistochilaphilippinensis*, Grolle 1966b, Pócs 1968, 1971, Tixier 1972, Miller et al. 1983 as *Gottscheaaligera*, *Schistochilasteraliger* and as *Schistochilasterphilippinensis*, Piippo 1984a also *Schistochilaphilippinensis*, Bapna and Kachroo 2000b, **So 2003a**. Seram: **Akiyama 1986, 2009** both also as *Schistochilaphilippinensis* and *Schistochilarecurvata*.

*Schistochilabeccariana* (De Not.) Trevis. **MOLUCCAS**: Ambon: **LECTOTYPE** of *Schistochilaamboinensis* [here designated, cf. So 2003a: 87 as “holotype”], **Stephani 1909c** as *Schistochilaamboinensis*, **So 2003a**.

*Schistochilablumei* (Nees) Trevis. **MOLUCCAS**: Tixier 1974a. Halmahera: **Sande Lacoste 1864** as *Gottscheablumei*, **Schiffner 1898** as *Schistochila ‘blumii*’, **Buch 1939**, Grolle 1966b, Pócs 1971, Kitagawa 1973, 1978, Miller et al. 1983, Piippo 1984a, Inoue 1985, Enroth 1991 as *Schistochila* ‘*blumii*’. Ambon: **So 2003a**. Seram: **Akiyama 1986, 2009** both as *Schistochilaformosana*.

*Schistochiladoriae* (De Not.) Trevis. **MOLUCCAS**: Ambon: **Schiffner 1893b** as ‘Schistocheila’ doriae, **1898**.

*Schistochilareinwardtii* (Nees) Schiffn. **MOLUCCAS**: Ambon: **So 2003a**.

Schistochilasciurea(Nees)Schiffn.var.sciurea. **MOLUCCAS**: Ambon: **Schiffner 1893b** as ‘Schistocheila’ sciurea f. robustior, **1898**, Verdoorn 1937, Hattori 1968, Miller et al. 1983, So 2003a. Seram: **LECTOTYPE** of *Schistochilainversa* [here designated, cf. So 2003a: 98 as “holotype”], **Herzog 1926b** as *Schistochilainversa*, Grolle 1966b as *Schistochilainversa*, Hässel 1976 as *Schistochilainversa*, **Akiyama 1986, 2009, So 2003a**.

***Schizophyllopsis* Váňa et L.Söderstr.**

*Schizophyllopsisbidens* (Reinw., Blume et Nees) Váňa et L.Söderstr. **MOLUCCAS**: Seram: **Akiyama 1986, 2009** both as *Anastrophyllumbidens*, Váňa and Piippo 1989b as Anastrophyllumbidensvar.bidens as *Anastrophyllumbidens*, Váňa 1991b as *Anastrophyllumbidens*, Schill and Long 2003 as *Anastrophyllumbidens*, Eggers 2006 as *Anastrophyllumbidens*.

***Solenostoma* Mitt.**

*Solenostomaariadne* (Taylor) R.M.Schust. ex Váňa et D.G.Long. **MOLUCCAS**: Ambon: **Sande Lacoste 1864** as *Jungermanniaariadne*, **Schiffner 1898**, 1900 both as *Nardiaariadne*, **Stephani 1901a** as *Jungermanniaariadne*, Amakawa 1968 as *Jungermanniaariadne*, Swanson and Miller 1969 as *Jungermanniaariadne*, Váňa 1972 as *Jungermanniaariadne* [rejecting report by Sande Lacoste 1864 as *J.tetragona*], Bonner 1976 as *Jungermanniaariadne*, Miller et al. 1983 as *Plectocoleaariadne*, Váňa and Piippo 1989a as *Jungermanniaariadne*. Seram: **Akiyama 1986, 2009** both as *Jungermanniaariadne*, Váňa and Piippo 1989a as *Jungermanniaariadne*, Váňa 1991a as *Jungermanniaariadne*, Bapna and Kachroo 2000a as *Jungermanniaariadne*.

Solenostomacomatum(Nees)C.Gaovar.comatum. **MOLUCCAS**: Ternate/Tidore: Sande **Lacoste 1856b** as *Plagiochilacomata*, **Schiffner 1898**, 1900 both as *Nardiacomata*, **Stephani 1901a** as *Jungermanniacomata*, Hattori 1951 as *Plectocoleacomata*, Bonner 1965 as *Eucalyx* ‘*comatus*’, Váňa 1972 as *Jungermanniacomata*, 1973 as *Jungermanniacomata* [with a ‘?’], Miller et al. 1983 as *Jungermanniacomata*, Váňa and Piippo 1989a as *Jungermanniacomata*.

*Solenostomatetragonum* (Lindenb.) R.M.Schust. ex Váňa et D.G.Long. **MOLUCCAS**: Ambon: **Váňa 1972**, 1973, 1975, 1991a all both as *Jungermanniatetragona*, Hattori 1975b as *Jungermanniatetragona*, Miller et al. 1983 as *Jungermanniatetragona*, Váňa and Piippo 1989a as *Jungermanniatetragona*, Bapna and Kachroo 2000a as *Jungermanniatetragona*, Srivastava and Sharma 2000 as *Jungermanniatetragona*, Easa 2003 as *Jungermanniatetragona*. Seram: **Akiyama 1986, 2009** both as *Jungermanniatetragona*, Váňa and Piippo 1989a as *Jungermanniatetragona*, Váňa 1991a as *Jungermanniatetragona*.

*Solenostomatruncatum* (Nees) R.M.Schust. ex Váňa et D.G.Long. **MOLUCCAS**: Halmahera: **Váňa and Piippo 1989a** as *Jungermanniatruncata*, Váňa 1991a as *Jungermanniatruncata*. Ambon: Váňa 1973, 1991a as *Jungermanniatruncata*, Váňa and Piippo 1989a as *Jungermanniatruncata*, Bapna and Kachroo 2000a as *Jungermanniatruncata*, **Akiyama 2009** as *Jungermanniatruncata*. Seram: **Akiyama 1986, 2009** both as *Jungermanniatruncata*, Váňa and Piippo 1989a as *Jungermanniatruncata*, Váňa 1991a as *Jungermanniatruncata*.

***Spruceanthus* Verd.**

*Spruceanthuspolymorphus* (Sande Lac.) Verd. **MOLUCCAS**: Halmahera: **Umagap 2019**. Seram: **Mizutani 1986a**, **Akiyama 2009**.

***Symphyogynopsis* Grolle**

*Symphyogynopsisgottscheana* (Mont. et Nees) Grolle. **MOLUCCAS**: Wigginton and Porley 2001, Schaumann et al. 2002. Seram: **Grolle and Piippo 1986** as *Symphyogynopsisfilicum*, **Grolle 1987**, Enroth 1991, Furuki 2002 as *Symphyogynopsisfilicum*.

***Syzygiella* Spruce**

*Syzygiellaovalifolia* Inoue. **MOLUCCAS**: Seram: **Akiyama 1986, 2009**, So and Grolle 2003.

*Syzygiellasecurifolia* (Nees) Inoue. **MOLUCCAS**: Halmahera: **Sande Lacoste 1864** as *Plagiochilavariegata*, **Schiffner 1898**, 1900 as *Syzygiellavariegata*, Miller et al. 1983 as *Syzygiellavariegata*. Seram: **Akiyama 1986, 2009**.

***Telaranea* Spruce ex Schiffn.**

*Telaraneamajor* (Herzog) J.J.Engel et G.L.Merr. **MOLUCCAS**: Schuster 2000a as *Arachniopsismajor*. Ambon: **Grolle 1968b** as *Arachniopsismajor*. Seram: **Akiyama 1986, 2009** as *Arachniopsismajor*, von Konrat et al. 2014.

*Telaraneatrisetosa* (Steph.) Grolle. **MOLUCCAS**: Seram: **Akiyama 1986, 2009**.

***Temnoma* Mitt.**

*Temnomasetigerum* (Lindenb.) R.M.Schust. **MOLUCCAS**: Seram: **Grolle 1967, Akiyama 1986, 2009**, Long and Grolle 1990, Bapna and Kachroo 2000a.

***Thysananthus* Lindenb.**

*Thysananthusappendiculatus* Steph. **MOLUCCAS**: Ambon: **Sukkharak 2015**. Seram: **Mizutani 1986a**, **Akiyama 2009**.

ThysananthusconvolutusLindenb.var.convolutus. **MOLUCCAS**: Haerida et al. 2010, Siregar et al. 2014, 2017, 2020a, Siregar 2015, Ginting and Batubara 2019. Buru: **Verdoorn 1934b**, Eggers and Schäfer-Verwimp 1987. Ambon: **Sukkharak 2011**. Seram: **Mizutani 1986a**, **Akiyama 2009**.

*Thysananthusfrauenfeldii* Reichardt. **MOLUCCAS**: Thiers and Gradstein 1989 as *Mastigolejeuneaundulata*, Gradstein et al. 2002 as *Mastigolejeuneaundulata*, Sass-Gyarmati 2003 as *Mastigolejeuneaundulata*, Sukkharak 2011 as *Thysananthusundulatus*. Seram: **Mizutani 1986a**, 1986b both as *Mastigolejeuneaundulata*, **Akiyama 2009** as *Mastigolejeuneaundulata*.

*Thysananthusfruticosus* (Lindenb. et Gottsche) Schiffn. **MOLUCCAS**: Mizutani 1977. Obi: **Verdoorn 1934b**. Banda: **Sande Lacoste 1864** as *Bryopterisfruticosa*, **Schiffner 1898**, Hattori 1951. Seram: **Sande Lacoste 1864** as *Bryopterisfruticosa*, **Verdoorn** 1934a, **1934b**, Hattori 1951, Miller et al. 1983, **Mizutani 1986a, 1987, Akiyama 2009**.

*Thysananthushumilis* (Gottsche) Sukkharak et Gradst. **MOLUCCAS**: Mizutani 1977 as *Mastigolejeuneahumilis*. Ambon: Stephani 1912a as *Mastigolejeuneahumilis*, **Verdoorn** 1934a, **1934b** both as *Mastigolejeuneahumilis*, Miller et al. 1983 as *Mastigolejeuneahumilis*, Awasthi and Udar 1984 as *Mastigolejeuneahumilis*, Bapna and Kachroo 2000b as Mastigolejeuneahumilisvar.humilis. Banda: **Verdoorn 1934b** as *Mastigolejeuneahumilis*, **Bonner 1963a** as *Brachiolejeuneamolukkensis*, Tixier 1966 as *Mastigolejeuneahumilis*, Bapna and Kachroo 2000b as *Mastigolejeuneahumilis*. Seram: **LECTOTYPE** of *Brachiolejeuneamolukkensis* [Bonner 1963a: 456], Awasthi and Udar 1984 as *Mastigolejeuneahumilis*, **Mizutani 1986a, 1986b** both as *Mastigolejeuneahumilis*, Bapna and Kachroo 2000b as *Mastigolejeuneahumilis*, **Akiyama 2009** as *Mastigolejeuneahumilis*.

*Thysananthusligulatus* (Lehm. et Lindenb.) Sukkharak et Gradst. **MOLUCCAS**: Buru: **Verdoorn 1934b** as *Mastigolejeunealigulata*, Swanson and Miller 1969 as *Mastigolejeunealigulata*, Miller et al. 1983 as *Mastigolejeunealigulata*. Ambon: **Mizutani 1986b** as *Mastigolejeunealigulata*. Seram: **Sande Lacoste 1864** as *Phragmicomaligulata*, **Schiffner 1898** as *Mastigolejeunealigulata*, Swanson and Miller 1969 as *Mastigolejeunealigulata*, Miller et al. 1983 as *Mastigolejeunealigulata*, **Mizutani 1986a, 1986b** both as *Mastigolejeunealigulata*, **Akiyama 2009** as *Mastigolejeunealigulata*, **Sukkharak and Gradstein 2014** as *Mastigolejeunealigulata*.

*Thysananthusrepletus* (Taylor) Sukkharak et Gradst. **MOLUCCAS**: Ambon: **Verdoorn 1934b** as *Mastigolejeuneaatypos*, Grolle 1968b as *Mastigolejeuneaatypos*, Miller et al. 1983 as *Mastigolejeuneaatypos*. Seram: **Verdoorn 1934b** as *Mastigolejeuneaatypos*, Grolle 1968b as *Mastigolejeuneaatypos*, Miller et al. 1983 as *Mastigolejeuneaatypos*.

*Thysananthusspathulistipus* (Reinw., Blume et Nees) Lindenb. **MOLUCCAS**: Mizutani 1977, Haerida et al. 2010, Siregar et al. 2014, Siregar 2015, Siregar et al. 2017. Ambon: **LECTOTYPE** of *Mastigolejeuneaamboinensis* [to be designated], **LECTOTYPE** of Mastigolejeuneaamboinensisvar.paucidentata [to be designated], **Sande Lacoste 1864** [Saparoea I], **Schiffner 1890** as *Mastigolejeuneaamboinensis*, **Schiffner 1890** as Mastigolejeuneaamboinensisvar.paucidentata, **1898, Sydow 1894** as *Mastigolejeuneaamboinensis*, **Verdoorn 1934b** [also Saparoea I], Swanson and Miller 1969, Miller et al. 1983, Bapna and Kachroo 2000b. Seram: **Sande Lacoste 1864**, **Schiffner 1898, Verdoorn** 1934a, **1934b**, Grolle 1965, Swanson and Miller 1969, **Mizutani 1986a**, **Akiyama 2009** as *Thysananthus* ‘*spatulistipus*’.

*Thysananthustruncatus* (Mizut.) Sukkharak et Gradst. **MOLUCCAS**: Seram: **Mizutani 1986a, 1986b** both as *Mastigolejeuneatruncata*, Pócs et al. 1994 as *Mastigolejeuneatruncata*, **Akiyama 2009** as *Mastigolejeuneatruncata*, **Sukkharak and Gradstein 2014** as *Mastigolejeuneatruncata*.

*Thysananthusvirens* Ångstr. **MOLUCCAS**: Haerida et al. 2010 as *Mastigolejeuneavirens*, Siregar et al. 2014, 2020a both as *Mastigolejeuneavirens*, Siregar 2015 as *Mastigolejeuneavirens*, Ginting and Batubara 2019 as *Mastigolejeuneavirens*, Siregar et al. 2020a as *Mastigolejeuneavirens*. Seram: **Mizutani 1986a** as *Mastigolejeuneavirens*, **Mizutani 1986b** as *Mastigolejeuneavirens*, **Akiyama 2009** as *Mastigolejeuneavirens*.

***Treubia* K.I.Goebel**

*Treubiainsignis* K.I.Goebel. **MOLUCCAS**: Seram: **Akiyama 1986, 2009**.

***Trichocolea* Dumort.**

*Trichocoleapluma* (Reinw., Blume et Nees) Mont. **MOLUCCAS**: Buru: **Herzog 1921** as *Trichocoleastriolata*. Ambon: **Schiffner 1893b, 1898** both as Trichocoleatomentellavar.javanica, **Katagiri et al. 2013**. Seram: **Akiyama 1986, 2009**.

*Trichocoleatomentella* (Ehrh.) Dumort. **MOLUCCAS**: Morotai: **New to Moluccas**: Morotai, Sangowo, *Exp. Kostermans*) (BO13087)]. Ambon: Miller et al. 1983 as *Trichocoleatomentella*, Bapna and Kachroo 2000a. Note: we are not aware of any first hand report from Ambon but it is here confirmed for Moluccas.

***Tricholepidozia* (R.M.Schust.) E.D.Cooper**

*Tricholepidoziakogiana* (Steph.) E.D.Cooper. **MOLUCCAS**: Seram: **Akiyama 1986, 2009** both as *Telaraneakogiana*.

*Tricholepidozianeesii* (Lindenb.) E.D.Cooper. **MOLUCCAS**: Halmahera: **Grolle 1966c** as *Telaraneaneesii*, Pócs 1971 as *Telaraneaneesii*, Mizutani 1974, 1976 both as *Telaraneaneesii*, Engel 1978 as *Telaraneaneesii*, Kitagawa 1978 as *Telaraneaneesii*, Yamaguchi 1983 as *Telaraneaneesii*, Miller 1986 as *Telaraneaneesii*, Enroth 1991 as *Telaraneaneesii*, Engel and Smith Merrill 2004 as *Telaraneaneesii*. Seram: Piippo 1984b as *Telaraneaneesii*, **Akiyama 1986, 2009** both as *Telaraneaneesii*.

***Tuyamaella* S.Hatt.**

*Tuyamaellaserratistipa* S.Hatt. **MOLUCCAS**: Seram: **Mizutani 1986a**, Zhu and So 1998a, 2000, **Akiyama 2009**.

***Wettsteinia* Schiffn.**

*Wettsteiniainversa* (Sande Lac.) Schiffn. **MOLUCCAS**: Seram: **Akiyama 1986, 2009**.

***Zantenia* (S.Hatt.) Váňa et J.J.Engel**

*Zanteniaborneensis* (Herzog) Váňa et J.J.Engel. **MOLUCCAS**: Seram: **Váňa and Piippo 1989b** as *Anastrophyllumborneense*, Váňa 1991b as *Anastrophyllumborneense*.

*Zanteniakarstenii* (Schiffn.) Váňa et J.J.Engel. **MOLUCCAS**: Herzog 1926a as *Anastrophyllumkarstenii*. Ambon: **LECTOTYPE** of *Anastrophyllumkarstenii* [Bonner 1962b: 73], **Schiffner** 1893a, **1893b, 1898** all as *Anastrophyllumkarstenii*, **Stephani 1901c** as *Anastrophyllumkarstenii*, **Bonner 1962b** as *Anastrophyllumkarstenii*, Hattori 1966 as *Anastrophyllumkarstenii*.

***Zoopsis* Hook.f. ex Gottsche, Lindenb. et Nees**

*Zoopsisliukiuensis* Horik. **MOLUCCAS: Grolle and Piippo 1984**. Ambon: **Grolle 1968b**, Schuster 1969, Miller et al. 1983, **Schuster 1999**, Engel and Glenny 2008. Seram: **Akiyama 1986, 2009**.

*Zoopsissetigera* K.I.Goebel. **MOLUCCAS**: Ambon: **LECTOTYPE** [Grolle and Piippo 1984: 303 as “neotype”], **Grolle and Piippo 1984, Schuster 1999**, Engel and Glenny 2008. Seram: **Akiyama 1986, 2009**.

### ﻿Names not referred to any accepted taxon

We are not able to refer the following published names to any accepted taxon.

***Phaeoceros* Prosk.**

*Phaeocerosvelutinus* J.Haseg. ex H.Akiyama *nom. inval.***MOLUCCAS**: Ambon: **ORIGINAL MATERIAL**. Seram: Akiyama 1986. Note: we have not found the name *Phaeocerosvelutinus* anywhere else than in Akiyama (1986) listing it from Moluccas, and we do not know where to refer it.

***Jungermannia* L.**

*Jungermanniagigantea* β *laxior* (Nees) Lindenb. **MOLUCCAS**: Ternate/Tidore: **SYNTYPE** of *Jungermanniagigantea* β *laxior* [lectotype to be designated], **ORIGINAL MATERIAL** of *Plagiochilatrapezoidea* γ *major*, **Nees 1830, 1831** both as *Jungermanniagigantea* β *laxior*, **Lindenberg 1840** as *Plagiochilatrapezoidea* γ ‘*maior*’, **Schiffner 1898**, 1900 as Plagiochilatrapezoideavar.major, **Geissler and Bischler 1987** as *Jungermanniagigantea* β *laxior*. Note: this name does have a confusing history and before someone really select and identify a **lectotype**, it is impossible to know what taxon it represents

Jungermanniatamarisci L. var. β minus-ramosa Schwägr. *nom. inval.***MOLUCCAS**: **ORIGINAL MATERIAL, Gaudichaud 1828**. Note: *Frullaniatamarisci* is a mainly Eurasian taxon and this name must refer to a tropical taxon

***Plagiochila* (Dumort.) Dumort.**

*Plagiochilaopposita* (Reinw., Blume et Nees) Lindenb. β *falcata* (Nees) Schiffn. **MOLUCCAS**: Ternate/Tidore: **Sande Lacoste 1856b, Schiffner 1898**. Note: *Plagiochilaopposita* is now *Chiastocaulonoppositum* but we are not sure if unranked β *falcata* also belong to that species and is worth recognizing.

### ﻿Taxa doubtfully occurring in Moluccas

We doubt the identification of the following taxa or we are not aware of any first-hand report from Moluccas.

***Acromastigum* A.Evans**

*Acromastigumechinatum* (Gottsche) A.Evans. **MOLUCCAS**: Ambon: Stephani 1909a as *Mastigobryumechinatum*, Bonner 1962b, Swanson and Miller 1969, Miller et al. 1983. Note: we are not aware of any first hand report from Moluccas although it is reported to occur on neighbouring islands.

***Bazzania* Gray**

*Bazzaniaceylanica* (Mitt.) Steph. **MOLUCCAS**: Ambon: Stephani 1902 as *Mastigobryumceylanicum*. Note: we are not aware of any first hand report from Moluccas although it is reported to occur on neighbouring islands.

*Bazzaniajaponica* (Sande Lac.) Lindb. **MOLUCCAS**: Siregar and Pasaribu 2019. Note: we are not aware of any first hand report from Moluccas although it is reported to occur on neighbouring islands.

***Calypogeia* Raddi**

*Calypogeiatosana* (Steph.) Steph. **MOLUCCAS**: Halmahera: **Umagap 2019**. Note: a Sino-Himalayan taxon that seems unlikely to occur in the tropics.

***Chiloscyphus* Corda**

*Chiloscyphusernstianus* Steph. **MOLUCCAS**: Seram: **Akiyama 1986, 2009** both as Lophocoleaaff.ernstiana. Note: we are not aware of any positively identified report from Moluccas. It is otherwise only known from the type from Sumatra and if worth recognizing it certainly belongs to some other genus.

***Cololejeunea* (Spruce) Steph.**

*Cololejeuneacordiflora* Steph. **MOLUCCAS**: Ambon: Tixier 1962 as *Cololejeuneakarstenii*. Note: we are not aware of any first hand report from Moluccas although it is reported to occur on neighbouring islands.

*Cololejeuneaverrucosa* Steph. **MOLUCCAS**: Miller 1968, Pócs and Ninh 2012, Pócs 2012. Ambon: Miller et al. 1983. Note: we are not aware of any first hand report from Moluccas although it is reported to occur on neighbouring islands.

***Cryptolophocolea* L.Söderstr., Crand.-Stotl., Stotler et Váňa**

*Cryptolophocoleaciliolata* (Nees) L.Söderstr., Crand.-Stotl., Stotler et Váňa. **MOLUCCAS**: Seram: **Akiyama 1986, 2009** both as Lophocoleaaff.ciliolata. Note: we are not aware of any positively identified report from Moluccas although it is reported to occur on neighbouring islands.

***Leptolejeunea* (Spruce) Steph.**

*Leptolejeuneabalansae* Steph. **MOLUCCAS**: Ambon: Jovet-Ast 1958. Note: we are not aware of any first hand report from Moluccas although it is reported to occur on neighbouring islands.

*Leptolejeuneasubacuta* Steph. ex A.Evans. **MOLUCCAS**: Inoue and Miller 1965, Miller 1968, Swanson and Miller 1969, Miller et al. 1983, Bapna and Kachroo 2000b. Note: we are not aware of any first hand report from Moluccas although it is reported to occur on neighbouring islands.

***Mastigophora* Nees**

*Mastigophorawoodsii* (Hook.) Nees. **MOLUCCAS**: Seram: **LECTOTYPE** of *Mastigophoraramentifissa* [to be designated], **Herzog** 1926a, **1926b** as *Mastigophoraramentifissa*, Inoue 1971. Note: Inoue (1971) synonymized *Mastigophoraramentifissa* with *Mastigophorawoodsii* but doubted the origin of the type of the former as *Mastigophorawoodsii* is unlikely to occur in the tropics.

***Plagiochila* (Dumort.) Dumort.**

*Plagiochilagracilis* Lindenb. et Gottsche. **MOLUCCAS**: Miller et al. 1983. Note: we are not aware of any first hand report from Moluccas although it is reported to occur on neighbouring islands.

***Radula* Dumort.**

*Radulaamentulosa* Mitt. **MOLUCCAS**: Seram: Yamada 1999. Note: we are not aware of any first-hand report from Moluccas.

***Schistochila* Dumort.**

*Schistochilaschultzei* Steph. **MOLUCCAS**: Seram: **Akiyama 1986, 2009** both as Schistochilaaff.schultzei. Note: we are not aware of any positively identified specimen from Moluccas although it is reported from neighboring islands.

***Spruceanthus* Verd.**

*Spruceanthussemirepandus* (Nees) Verd. **MOLUCCAS**: Haerida et al. 2010. Note: we are not aware of any positively identified specimen from Moluccas although it is reported from neighboring islands.

***Thysananthus* Lindenb.**

*Thysananthusauriculatus* (Wilson et Hook.) Sukkharak et Gradst. **MOLUCCAS**: Haerida et al. 2010 as *Mastigolejeuneaauriculata*, Singh et al. 2010 as *Mastigolejeuneaauriculata*. Note: we are not aware of any positively identified specimen from Moluccas although it is reported from neighboring islands.

### ﻿Taxa reported but rejected from Moluccas

The following taxa are reported to occur in Moluccas but the reports are rejected either earlier or here as misidentified or highly unlikely to occur in the area.

***Cheilolejeunea* (Spruce) Steph.**

*Cheilolejeuneamariana* (Gottsche) B.M.Thiers et Gradst. **MOLUCCAS**: Seram: **Mizutani 1986a** as *Spruceanthusmarianus*, **Akiyama 2009** as *Spruceanthusmarianus*. Note: a Pacific species frequently, but erroneously, reported from SE Asia and many other areas. Reports should mainly be referred to *Archilejeuneaplaniuscula*.

***Chiastocaulon* Carl**

*Chiastocaulonfimbriatum* (Mitt.) S.D.F.Patzak, M.A.M.Renner, Schäf.-Verw. et Heinrichs. **MOLUCCAS**: Seram: **Akiyama 1986, 2009** both as Plagiochilionaff.fimbriatum. Note: a Sino-Himalayan taxon very unlikely to occur in the tropics.

***Conocephalum* Hill**

*Conocephalumconicum* (L.) Dumort. **MOLUCCAS**: Ambon: Sopacua et al. 2020 as ‘Conocephelum’ conicum [Saparua I]. Note: presumably a mainly European taxon reported *sensu lato* from many other places. It is unlikely that the genus *Conocephalum* occurs in the tropics, but if it is a *Conocephalum* sp. it is probably more related to *Conocephalumsalebrosum* than to *Conocephalumconicum*.

***Cryptolophocolea* L.Söderstr., Crand.-Stotl., Stotler et Váňa**

*Cryptolophocoleaconnata* (Sw.) L.Söderstr. et Váňa. **MOLUCCAS**: **Gaudichaud 1828** as *Jungermanniaconnata*. Note: an American species and this old record must be rejected as either mis-identified or mis-labelled.

***Dendroceros* Nees**

*Dendrocerosexalatus* Steph. **MOLUCCAS**: Ambon: **LECTOTYPE** [Hasegawa 1980: 302], **Stephani 1909b**, 1917, **Bonner 1965**. Note: Stephani erroneously reported the type locality as “Amboina” but as this is a Brazilian species that must be due to mislabeling (cf. Hasegawa 1980).

***Heteroscyphus* Schiffn.**

*Heteroscyphuscaledonicus* (Steph.) Schiffn. **MOLUCCAS**: Ambon: Miller et al. 1983. Note a Pacific species possibly endemic to New Caledonia.

***Lepicolea* Dumort.**

*Lepicoleaochroleuca* (Spreng.) Spruce. **MOLUCCAS**: Seram: **Herzog 1921**. Note: an Afro-American species only once reported from SE Asia. This report must be an error.

*Lepicoleascolopendra* (Hook.) Dumort. ex Trevis. **MOLUCCAS**: Seram: **Herzog 1926b**. Note: an Australian/New Zealandian species frequently, but erroneously, reported from many other places.

***Lepidozia* (Dumort.) Dumort.**

*Lepidoziamicrophylla* (Hook.) Lindenb. **MOLUCCAS**: Seram: Siregar and Pasaribu 2019. Note: a species endemic to New Zealand (cf. Engel and Schuster 2001) and all reports from other areas must be rejected.

*Lepidoziarigida* Steph. **MOLUCCAS**: Buru: **ORIGINAL MATERIAL** of Lepidoziarigidaf.minor, **Herzog 1921** as Lepidoziarigidaf.minor, Herzog 1926a as Lepidoziarigidavar.minor. Note: a species from Vanuatu. Herzog’s material must belong to another species.

***Plagiochila* (Dumort.) Dumort.**

*Plagiochilagigantea* Lindenb. **MOLUCCAS**: Ternate/Tidore: **Nees 1830** as *Jungermanniagigantea*. Note: a New Zealand taxon.

*Plagiochilatrapezoidea* Lindenb. **MOLUCCAS**: Ternate/Tidore: **Reinwardt et al. 1824** as *Jungermanniasimplex* Web, Schiffner 1900, Bapna and Kachroo 2000b. Note: Inoue (1984) designated the only specimen from Java of *Jungermanniasimplex* Web. that he could locate as a neotype of *Plagiochilatrapezoidea* and *Plagiochilatrapezoidea* β *tenera* is described as based on *Jungermanniasimplex* Web. However, Inoue (1984) reject the report of β *tenera* in Gottsche et al. (1844; all other reports probably based on this) as *Plagiochilaciliata*. The occurrence of *Plagiochilatrapezoidea* in Moluccas is therefore not confirmed and reports should be rejected following Inoue, until confirmed, although it occurs in most neighbouring islands.

***Podomitrium* Mitt.**

*Podomitriumphyllanthus* (Hook.) Mitt. **MOLUCCAS**: Ambon: **Schiffner 1890, 1898** as ‘*Hymenophytum’ phyllanthus*, **Sydow 1894**. Note: this is an Australian/New Zealandian species.

***Porella* L.**

*Porellanitens* (Steph.) S.Hatt. **MOLUCCAS**: Long and Grolle 1990. Note: the second-hand reports from Malesia in Long and Grolle (1990) of this Sino-Himalayan species is due to supposed, but rejected, synonymy with *Porellajavanica* (cf. also Hattori 1978).

***Pycnolejeunea* (Spruce) Schiffn.**

*Pycnolejeuneaschwaneckei* (Steph.) Schiffn. ex P.Syd. **MOLUCCAS**: Ambon: **Schiffner 1890**, **Sydow 1894** as *Pycnolejeunea* ‘*schwaneckii*’. Note: this is a neotropical species.

***Radula* Dumort.**

*Radulapinnulata* Mitt. **MOLUCCAS**: Miller et al. 1983. Note: Yamada (2002) rejected all reports from Moluccas.

***Riccardia* Gray**

*Riccardialatifrons* (Lindb.) Lindb. **MOLUCCAS**: Ambon: **Schiffner 1890** as *Aneuralatifrons*, **Sydow 1894** as *Aneuralatifrons*. Note: a mainly boreal species and all reports from tropical areas should be rejected.

***Schistochila* Dumort.**

*Schistochilaneesii* (Mont.) Lindb. **MOLUCCAS**: Ambon: **Schiffner 1893b, 1898**, Buch 1939, Hässel 1976 as *Paraschistochilaneesii*. Note: an African species rejected from Moluccas by So (2003a).

***Symbiezidium* Trevis.**

*Symbiezidiumtransversale* (Sw.) Trevis. **MOLUCCAS**: **Gaudichaud 1828** as *Jungermanniatransversalis*. Note: an American species.

***Zoopsis* Hook.f. ex Gottsche, Lindenb. et Nees**

*Zoopsisargentea* (Hook.f. et Taylor) Gottsche, Lindenb. et Nees. **MOLUCCAS**: Ambon: Stephani 1908a, Del Rosario 1975b, Miller et al. 1983. Note: an Australian/New Zealandian species.

*Zoopsissetulosa* Leitg. **MOLUCCAS**: Miller et al. 1983 as *Zoopsisrigida*. Ambon: **Schiffner 1893b** as *Cephaloziasetulosa*, **1898, Stephani 1908b**, Hattori 1951, Hodgson 1965, Schuster 1969. Note: an Australian/New Zealandian/ New Caledonian species rejected from Asia as *Zoopsissetigera* by Grolle and Piippo (1984).

### ﻿Synonyms

*Acrolejeuneadensifolia* (Schiffn.) Schiffn. ex P.Syd. *nom. inval*. = *Schiffneriolejeuneapulopenangensis*

*Acrolejeuneadensifolia* Schiffn. *nom. inval*. = *Schiffneriolejeuneapulopenangensis*

*Acrolejeuneahaskarliana* (Gottsche) Schiffn. ≡ Schiffneriolejeuneatumidavar.haskarliana

*Acrolejeuneapulopenangensis* (Gottsche) Schiffn. ≡ *Schiffneriolejeuneapulopenangensis*

*Acrolejeunearostrata* Schiffn. *nom. inval*. = Acrolejeuneapycnocladasubsp.pycnoclada

*Acrolejeunearostrata* var. α *minor* Schiffn. *nom. inval*. = Acrolejeuneapycnocladasubsp.pycnoclada

Acrolejeunearostrata var. β major Schiffn. *nom. inval*. = Acrolejeuneapycnocladasubsp.pycnoclada

*Acrolejeuneasecurifolia* (Nees) Steph. *nom. inval*. ≡ *Acrolejeuneasecurifolia*

*Acromastigumbidenticulatum* A.Evans = *Acromastigumbancanum*

*Anastrophyllumbidens* (Reinw., Blume et Nees) Steph. ≡ *Schizophyllopsisbidens*

*Anastrophyllumborneense* Herzog ≡ *Zanteniaborneensis*

*Anastrophyllumcontractum* (Reinw., Blume et Nees) Steph. ≡ *Cuspidatulacontracta*

*Anastrophyllumkarstenii* Schiffn. ≡ *Zanteniakarstenii*

*Anastrophyllumrevolutum* Steph. ≡ *Anastrophyllopsisrevoluta*

*Aneuraalbomarginata* Steph. ≡ *Riccardiaalbomarginata*

*Aneurakarstenii* Steph. ≡ *Riccardiakarstenii*

*Aneuralatifrons* Lindb. ≡ *Riccardialatifrons*

Aneurapinguisf.normalis Schiffn. *nom. inval*. = *Aneurapinguis*

*Anomacaulisflaccidus* (Steph.) Grolle ≡ *Cuspidatulaflaccida*

*Anthocerosamboinensis* Schiffn. ≡ *Foliocerosamboinensis*

*Anthocerosappendiculatus* Steph. ≡ *Foliocerosappendiculatus*

*Anthocerosglandulosus* Lehm. et Lindenb. ≡ *Foliocerosglandulosus*

*Anthocerosgrandis* Ångstr. = *Megacerosflagellaris*

*Arachniopsismajor* Herzog ≡ *Telaraneamajor*

*Aspiromitusamboinensis* (Schiffn.) Steph. ≡ *Foliocerosamboinensis*

*Bazzaniaaustralis* (Mont.) Trevis. = *Bazzaniatridens*

*Bazzaniaconcinna* (De Not.) Trevis. = *Bazzaniaintermedia*

*Bazzaniadivaricata* (Nees) Trevis. = *Acromastigumdivaricatum*

*Bazzaniaechinatiformis* (De Not.) Trevis. ≡ *Acromastigumechinatiforme*

*Bazzaniaintermedia* auct. = Bazzaniatridensvar.tridens

*Bazzanialongidens* (Steph.) Schiffn. = *Bazzaniaconophylla*

*Bazzaniaoshimensis* (Steph.) Horik. = *Bazzaniatridens*

*Brachiolejeuneamolukkensis Steph*. = *Thysananthushumilis*

*Bryopterisfruticosa* Lindenb. et Gottsche ≡ *Thysananthusfruticosus*

*Calypogeiafusca* (Lehm.) Steph. ≡ *Mniolomafuscum*

*Campylolejeuneaciliatilobula* (Schiffn.) S.Hatt. = *Cololejeuneainflectens*

*Campylolejeuneainflectens* (Mitt.) Mizut. ≡ *Cololejeuneainflectens*

*Caudalejeuneacircinata* Steph. = *Caudalejeuneacristiloba*

*Caudalejeunearecurvistipula* (Gottsche) Schiffn. = *Caudalejeuneareniloba*

*Cephaloziasetulosa* (Leitg.) Spruce ≡ *Zoopsissetulosa*

*Chandonanthusgracilis* Herzog = *Plicanthushirtellus*

*Chandonanthushirtellus* (F.Weber) Mitt. ≡ *Plicanthushirtellus*

*Cheilolejeuneaimbricata* (Nees) S.Hatt. = *Cheilolejeuneatrapezia*

*Cheilolejeuneameyeniana* (Nees, Lindenb. et Gottsche) R.M.Schust. et Kachroo = *Cheilolejeuneatrapezia*

*Chiloscyphusamboinensis* (Schiffn.) Schiffn. ≡ *Heteroscyphusamboinensis*

*Chiloscyphusaselliformis* (Reinw., Blume et Nees) Nees ≡ *Heteroscyphusaselliformis*

*Chiloscyphuscoalitus* (Hook.) Nees ≡ *Heteroscyphuscoalitus*

*Chiloscyphusdecurrens* (Reinw., Blume et Nees) Nees = *Heteroscyphussplendens*

Chiloscyphusdecurrensvar.amboinensis (Schiffn.) Schiffn. ex Bonner *nom. inval*. ≡ *Heteroscyphusamboinensis*

*Chiloscyphusendlicherianus* δ *amboinensis* Schiffn. ≡ *Heteroscyphusamboinensis*

*Chiloscyphusfalcifolius* Steph. = *Acroscyphellatjiwideiensis*

*Chiloscyphusgranulatus* Schiffn. = *Saccogynidiumrigidulum*

*Clasmatocoleatjiwideiensis* (Sande Lac.) Grolle ≡ *Acroscyphellatjiwideiensis*

*Cololejeuneaciliatilobula* (Schiffn.) Schiffn. = *Cololejeuneainflectens*

*Cololejeuneacrenulata* (Herzog) Benedix *nom. inval.* = *Cololejeuneaangustiflora*

*Cololejeuneafalcatoides* Benedix = *Cololejeuneafalcata*

*Cololejeuneagoebelii* (Gottsche ex Schiffn.) Schiffn. = *Cololejeuneatrichomanis*

*Cololejeuneajavanica* (Steph.) Mizut. = *Cololejeuneaangustiflora*

*Cololejeuneakarstenii* (Steph.) Benedix = *Cololejeuneacordiflora*

*Cololejeuneanymannii* (Steph.) Benedix = *Cololejeuneaobliqua*

*Cololejeuneapeculiaris* (Herzog) Benedix = *Cololejeuneainflectens*

*Cololejeuneayulensis* (Steph.) Benedix = *Cololejeuneaaequabilis*

*Coluraparadoxa* (Schiffn.) Steph. ≡ *Calatholejeuneaparadoxa*

*Colurolejeuneakarstenii* (K.I.Goebel) Steph. ≡ *Colurakarstenii*

Colurolejeuneakarsteniivar.latifolia (Schiffn.) Schiffn. *nom. inval*. = *Colurakarstenii*

*Colurolejeuneaparadoxa* (Schiffn.) Schiffn. ≡ *Calatholejeuneaparadoxa*

*Colurolejeuneasuperba* (Mont.) Steph. ≡ *Colurasuperba*

*Colurolejeuneasuperba* Schiffn. *nom. inval*. = *Coluraamboinensis*

*Colurolejeuneasuperba* var. α *typica* Schiffn. *nom. inval*. = *Coluraamboinensis*

*Dendroceroskarstenii* Schiffn. ex Steph. = *Dendrocerosacutilobus*

*Drepanolejeuneadactylophora* (Nees, Lindenb. et Gottsche) Schiffn. ≡ *Drepanolejeuneadactylophora*

*Drepanolejeuneadentata* Steph. ≡ *Lejeuneastenodentata*

*Drepanolejeuneafilicuspis* Steph. ≡ *Microlejeuneafilicuspis*

Drepanolejeunealevicornuavar.longicornua Herzog ≡ *Drepanolejeunealongicornua*

*Drepanolejeuneamicholitzii* Steph. = *Drepanolejeuneapentadactyla*

Drepanolejeuneamicholitziivar.dactylophoroides Herzog ≡ Drepanolejeuneapentadactylavar.dactylophoroides

Drepanolejeuneamicholitziivar.genuina Herzog *nom. illeg.* = *Drepanolejeuneapentadactyla*

Drepanolejeuneateneravar.genuina Herzog *nom. illeg*. = *Drepanolejeuneatenera*

*Drepanolejeuneaternatensis* (Gottsche) Steph. *nom. inval*. ≡ *Drepanolejeuneaternatensis*

*Eucalyxcomata* (Nees) Verd. ≡ *Solenostomacomatum*

*Eulejeuneafissistipula* Steph. ≡ *Lejeuneafissistipula*

*Eulejeuneaparallela* (Schiffn.) Schiffn. = *Metalejeuneacucullata*

*Euosmolejeuneaheterophylla* (Sande Lac.) Schiffn. = *Cheilolejeuneatrifaria*

*Euosmolejeuneaintegristipula* Steph. = *Cheilolejeunealindenbergii*

*Euosmolejeuneatrifaria* (Reinw., Blume et Nees) Steph. ≡ *Cheilolejeuneatrifaria*

*Frullaniaamboinensis* Schiffn. = Frullaniaintermediasubsp.intermedia

*Frullaniabillardiereana* Nees et Mont. = Frullaniaintermediasubsp.intermedia

*Frullaniadapitana* Steph. = *Frullanianodulosa*

Frullaniaericoidesvar.planescens (Verd.) S.Hatt. = *Frullaniaericoides*

Frullaniagracilisvar.lacerifolia (Steph.) Verd. = *Frullaniagracilis*

Frullaniaintermediaf.billardiereana (Nees et Mont.) Verd. = Frullaniaintermediasubsp.intermedia

Frullaniaintermediavar.amboinensis (Schiffn.) Verd. = Frullaniaintermediasubsp.intermedia

*Frullaniakarstenii* Schiffn. = *Frullaniaapiculata* var. goebelii

*Frullanialacerifolia* Steph. = *Frullaniagracilis*

Frullanianodulosaf.dapitana (Steph.) Verd. = *Frullanianodulosa*

Frullanianodulosaf.irreflexa Verd. = *Frullanianodulosa*

*Frullanianodulosa* β *plana* Schiffn. = *Frullanianodulosa*

*Frullaniapicta* Steph. = *Frullaniatrichodes*

*Frullaniareplicata* (Nees) Mont. = *Frullanianodulosa*

*Frullaniarugosa* Mitt. = *Frullaniagrandistipula*

Frullaniaserrataf.crispulo-dentata Verd. = Frullaniaserratavar.serrata

*Frullaniasquarrosa* (Mont.) Nees = *Frullaniaericoides*

Frullaniasquarrosaf.campanuloides Verd. = *Frullaniaericoides*

Frullaniasquarrosavar.planescens Verd. = *Frullaniaericoides*

*Frullaniatenuicaulis* Mitt. = *Frullaniatrichodes*

*Frullaniathuillieri* Nees = *Frullanianodulosa*

*Gamoscyphusaselliformis* (Reinw., Blume et Nees) Trevis. ≡ *Heteroscyphusaselliformis*

*Gottscheaaligera* (Nees et Blume) Nees ≡ *Schistochilaaligera*

*Gottscheablumei* (Nees) Nees ≡ *Schistochilablumei*

*Harpalejeuneaexigua* Steph. = *Lejeuneaexilis*

*Harpalejeuneafilicuspis* (Steph.) Mizut. ≡ *Microlejeuneafilicuspis*

*Herbertusarmitanus* (Steph.) H.A.Mill. = *Herbertussendtneri*

*Herbertusdivaricatus* (Herzog) H.A.Mill. = *Herbertussendtneri*

*Herbertusjavanicus* (Steph.) H.A.Mill. = *Herbertusramosus*

*Herbertusjavanicus* (Steph.) Schiffn. *nom. inval.* = *Herbertusramosus*

*Heteroscyphusdecurrens* (Nees) Schiffn. = *Heteroscyphussplendens*

*Hygrolejeuneaamboinensis* (Schiffn.) Schiffn. *nom. inval.* = *Lejeuneasordida*

*Hygrolejeuneaamboinensis* (Schiffn.) Schiffn. ex P.Syd. = *Lejeuneasordida*

*Hygrolejeuneadecurvifolia* Steph. = *Lepidolejeuneabidentula*

*Hygrolejeuneadiscreta* (Lindenb.) Schiffn. ≡ *Lejeuneadiscreta*

*Hygrolejeuneadiscreta* (Lindenb.) Steph. *nom. inval.* ≡ *Lejeuneadiscreta*

*Hygrolejeuneaparkinsonii* Steph. = *Lejeuneasordida*

*Hygrolejeuneasordida* (Nees) Schiffn. ≡ *Lejeuneasordida*

*Hygrolejeuneasordida* (Nees) Steph. *nom. inval.* ≡ *Lejeuneasordida*

*Hygrolejeuneaumbilicata* (Nees) Steph. *nom. inval.* ≡ *Lejeuneaumbilicata*

*Hymenophytonmalaccense* Steph. ≡ *Podomitriummalaccense*

*Hymenophytonphyllanthus* (Hook.) Steph. ≡ *Podomitriumphyllanthus*

*Jamesoniellacontracta* (Reinw., Blume et Nees) N.Kitag. ≡ *Cuspidatulacontracta*

*Jamesoniellaflexicaulis* (Nees) Schiffn. ≡ *Cuspidatulaflexicaulis*

Jamesoniellaflexicaulis var. β microphylla (Nees) Schiffn. = *Gottscheliaschizopleura*

*Jamesoniellamicrophylla* (Nees) Schiffn. = *Gottscheliaschizopleura*

*Jamesoniellaovifolia* (Schiffn.) Schiffn. = *Denotarisialinguifolia*

*Jungermanniaariadne* Taylor ≡ *Solenostomaariadne*

*Jungermanniaaselliformis* Reinw., Blume et Nees ≡ *Heteroscyphusaselliformis*

*Jungermanniacomata* Nees ≡ *Solenostomacomatum*

*Jungermanniaconnata* Sw. ≡ *Cryptolophocoleaconnata*

*Jungermanniacontracta* Reinw., Blume et Nees ≡ *Cuspidatulacontracta*

*Jungermanniaflexicaulis* Nees ≡ *Cuspidatulaflexicaulis*

*Jungermanniaflexicaulis* β *microphylla* Gottsche, Lindenb. et Nees = *Gottscheliaschizopleura*

*Jungermanniagigantea* Hook. *nom. illeg*. ≡ *Plagiochilagigantea*

*Jungermanniagigantea* β *laxior* Nees ≡ *Plagiochilagigantealaxior*

*Jungermanniaindica* Nees = Lejeuneaflavasubsp.orientalis

*Jungermanniaopposita* Reinw., Blume et Nees ≡ *Chiastocaulonoppositum*

*Jungermanniaovifolia* Schiffn. = *Denotarisialinguifolia*

*Jungermanniasimplex* Weber *nom. illeg.* = *Plagiochilatrapezoidea*

*Jungermanniatetragona* Lindenb. ≡ *Solenostomatetragonum*

*Jungermanniatransversalis* Sw. ≡ *Symbiezidiumtransversale*

*Jungermanniatruncata* Nees ≡ *Solenostomatruncatum*

*Jungermanniavaginata* Sw. ≡ *Frullaniavaginata*

*Lejeuneaapplanata* (Reinw., Blume et Nees) Nees ≡ *Lopholejeuneaapplanata*

*Lejeuneacatanduana* (Steph.) H.A.Mill., Bonner et Bischl. = *Lejeuneaanisophylla*

*Lejeuneachalmersii* (Steph.) Mizut. = *Lejeuneamicroloba*

*Lejeuneaconnivens* Gottsche = *Cheilolejeuneaceylanica*

*Lejeuneacucullata* (Reinw., Blume et Nees) Nees ≡ *Metalejeuneacucullata*

*Lejeuneahaskarliana* (Gottsche) Spruce *nom. illeg*. ≡ Schiffneriolejeuneatumidavar.haskarliana

*Lejeuneaheterophylla* Sande Lac. = *Cheilolejeuneatrifaria*

*Lejeuneamicrostipula* (Steph.) Prantl = *Lejeuneaexilis*

*Lejeuneaparadoxa* Schiffn. ≡ *Calatholejeuneaparadoxa*

*Lejeuneapusilla* (K.I.Goebel) Schiffn. *nom. illeg.* = *Cololejeuneametzgeriopsis*

*Lejeunearecurvistipula* Gottsche = *Caudalejeuneareniloba*

*Lejeunearostrata* var. α *minor* Schiffn. ex P.Geissler et Bischl. *nom. inval*. = Acrolejeuneapycnocladasubsp.pycnoclada

*Lejeuneasagrana* (Mont.) Gottsche, Lindenb. et Nees = *Lopholejeuneasubfusca*

Lejeuneasagranavar.dentistipula Schiffn. = *Caudalejeuneareniloba*

*Lejeuneasuperba* var. α *typica* Schiffn. *nom. inval.* = *Colurasuperba*

*Lejeuneaternatensis* Gottsche ≡ *Drepanolejeuneaternatensis*

*Lejeuneatrapezia* (Nees) Nees ≡ *Cheilolejeuneatrapezia*

*Lejeuneatrifaria* (Reinw., Blume et Nees) Nees ≡ *Cheilolejeuneatrifaria*

*Lepidoziapapulosa* Steph. ≡ *Neolepidoziapapulosa*

Lepidoziarigidaf.minor (Steph.) Herzog *nom. inval.* = *Lepidoziarigida*

Lepidoziarigidavar.minor (Steph.) Herzog *nom. inval*. = *Lepidoziarigida*

*Lepidoziawallichiana* Gottsche ≡ *Neolepidoziawallichiana*

*Leptolejeuneafoliicola* (Horik.) R.M.Schust. *nom. illeg*. ≡ *Drepanolejeuneafoliicola*

Leptolejeuneaschiffnerif.angustifolia Herzog *nom. inval*. = *Leptolejeuneaschiffneri*

Leptolejeuneaschiffnerivar.genuina Herzog *nom. inval*. = *Leptolejeuneaschiffneri*

*Leptolejeunea*schiffneri (Steph. ex Schiffn.) Steph. = *Leptolejeuneaschiffneri*

*Lobatiriccardialobata* (Schiffn.) Furuki = *Lobatiriccardiacoronopus*

*Lophocoleaciliolata* (Nees) Gottsche ≡ *Cryptolophocoleaciliolata*

*Lophocoleaernstiana* (Steph.) N.Kitag. ≡ *Chiloscyphusernstianus*

*Lophocoleagiulianettii* Steph. = *Cryptolophocoleacostata*

*Lopholejeuneadentistipula* (Schiffn.) Schiffn. = *Caudalejeuneareniloba*

*Lopholejeuneaeulopha* (Taylor) Steph. *nom. inval*. ≡ *Lopholejeuneaeulopha*

*Lopholejeuneajavanica* (Nees) Schiffn. = *Lopholejeuneanigricans*

*Lopholejeuneapyriflora* Steph. = *Lopholejeuneasubfusca*

*Lopholejeuneasagrana* (Mont.) Steph. = *Lopholejeuneasubfusca*

Lopholejeuneasagranavar.dentistipula (Schiffn.) Schiffn. ex P.Syd. = *Caudalejeuneareniloba*

Lopholejeuneasagranavar.dentistipula Schiffn. *nom. inval*. = *Caudalejeuneareniloba*

Lopholejeuneasagrana var. β subfusca (Nees) Schiffn. ≡ *Lopholejeuneasubfusca*

*Madothecaacutifolia* Lehm. et Lindenb. ≡ *Porellaacutifolia*

*Madothecacrenilobula* Herzog = *Porellajavanica*

*Marchantiaamboinensis* Nees et Mont. = Marchantiaemarginatasubsp.emarginata

*Mastigobryumamboinense* Steph. = *Bazzaniatridens*

*Mastigobryumasymmetricum* Steph. ≡ *Bazzaniaasymmetrica*

*Mastigobryumceylanicum* Mitt. ≡ *Bazzaniaceylanica*

*Mastigobryumconcinnum* De Not. = *Bazzaniaintermedia*

*Mastigobryumcrassitextum* (Steph.) Steph. ≡ *Bazzaniacrassitexta*

*Mastigobryumdeningeri* Herzog = *Bazzanialongicaulis*

*Mastigobryumechinatiforme* De Not. ≡ *Acromastigumechinatiforme*

*Mastigobryumechinatum* Gottsche ≡ *Acromastigumechinatum*

*Mastigobryumfallax* Sande Lac. ≡ *Bazzaniafallax*

*Mastigobryumhorridulum* (Schiffn.) Steph. ≡ *Bazzaniahorridula*

*Mastigobryuminsigne* De Not. ≡ *Bazzaniainsignis*

*Mastigobryumintermedium* Gottsche et Lindenb. ≡ *Bazzaniaintermedia*

*Mastigobryumirregulare* Steph. ≡ *Bazzaniairregularis*

*Mastigobryumjavanicum* Sande Lac. ≡ *Bazzaniajavanica*

*Mastigobryumlongidens* Steph. = *Bazzaniaconophylla*

*Mastigobryummerrillanum* Steph. ≡ *Bazzaniamerrillana*

*Mastigobryumpectinatum* (Nees) Lindenb. et Gottsche = *Bazzaniapectinata*

*Mastigobryumstresemannii* Herzog ≡ *Bazzaniastresemannii*

*Mastigobryumsubtile* Sande Lac. ≡ *Bazzaniasubtilis*

*Mastigobryumtridens* (Reinw., Blume et Nees) Dumort. ≡ *Bazzaniatridens*

*Mastigobryumuncigerum* (Reinw., Blume et Nees) Nees ≡ *Bazzaniauncigera*

*Mastigobryumvittatum* Gottsche ≡ *Bazzaniavittata*

*Mastigobryumwallichianum* Lindenb. ≡ *Bazzaniawallichiana*

*Mastigolejeuneaamboinensis* (Schiffn.) Schiffn. ex P.Syd. = *Thysananthusspathulistipus*

*Mastigolejeuneaamboinensis* Schiffn. *nom. inval.* = *Thysananthusspathulistipus*

Mastigolejeuneaamboinensis var. β paucidentata Schiffn. *nom. inval*. = *Thysananthusspathulistipus*

*Mastigolejeuneaatypos* Schiffn. ex Sydow = *Thysananthusrepletus*

*Mastigolejeuneaauriculata* (Wilson et Hook.) Steph. ≡ *Thysananthusauriculatus*

*Mastigolejeuneahumilis* (Gottsche) Schiffn. ≡ *Thysananthushumilis*

*Mastigolejeunealigulata* (Lehm. et Lindenb.) Schiffn. ≡ *Thysananthusligulatus*

*Mastigolejeuneatruncata* Mizut. ≡ *Thysananthustruncatus*

*Mastigolejeuneaundulata* Gradst. et Grolle = *Thysananthusfrauenfeldii*

*Mastigolejeuneavirens* (Ångstr.) Steph. ≡ *Thysananthusvirens*

Mastigophoradiclados var. β scorpioides (Reinw., Blume et Nees) Schiffn. = *Mastigophoradiclados*

*Mastigophoraramentifissa* Herzog = *Mastigophorawoodsii*

*Mastigophoravrieseana* (Sande Lac.) Schiffn. = *Mastigophoradiclados*

*Megacerosgrandis* (Ångstr.) Steph. = *Megacerosflagellaris*

*Metahygrobiellastolonacea* (Herzog) R.M.Schust. ≡ *Cephaloziastolonacea*

*Metzgeriadecipiens* (C.Massal.) Schiffn. = *Metzgeriaciliata*

*Metzgeriahamatiformis* Schiffn. = Metzgerialeptoneuravar.leptoneura

*Metzgeriopsispusilla* K.I.Goebel = *Cololejeuneametzgeriopsis*

*Microlejeuneamicrostipula* Steph. = *Lejeuneaexilis*

*Microlejeuneaparallela* Schiffn. = *Metalejeuneacucullata*

*Microlejeuneaternatensis* (Gottsche) Herzog nom. inval. ≡ *Drepanolejeuneaternatensis*

*Microlepidoziagonyotricha* (Sande Lac.) Del Ros. ≡ *Kurziagonyotricha*

*Nardiaariadne* (Taylor) Schiffn. ≡ *Solenostomaariadne*

*Nardiacomata* (Nees) Schiffn. ≡ *Solenostomacomatum*

*Noguchiaopposita* (Reinw., Blume et Nees) Inoue ≡ *Chiastocaulonoppositum*

*Omphalanthusumbilicatus* (Nees) Nees ≡ *Lejeuneaumbilicata*

*Otolejeuneasemperiana* (Steph.) Grolle ≡ *Allorgellasemperiana*

*Paraschistochilaneesii* (Mont.) R.M.Schust. ≡ *Schistochilaneesii*

*Phaeoceroshirticalyx* (Steph.) J.Haseg. ≡ *Phaeomegaceroshirticalyx*

Phaeoceroslaevissubsp.carolinianus (Michx.) Prosk. ≡ *Phaeoceroscarolinianus*

*Phaeocerospolyandrus* (Steph.) J.Haseg. = *Phaeomegaceroshirticalyx*

*Phragmicomafertilis* (Reinw., Blume et Nees) Nees ex Mont. ≡ *Acrolejeuneafertilis*

*Phragmicomaligulata* (Lehm. et Lindenb.) Gottsche, Lindenb. et Nees ≡ *Thysananthusligulatus*

*Phragmicomareniloba* Gottsche ≡ *Caudalejeuneareniloba*

*Physocoleaciliatilobula* (Schiffn.) Steph. = *Cololejeuneainflectens*

*Physocoleahamata* (Steph.) Steph. ≡ *Cololejeuneahamata*

*Plagiochilaacanthophylla* Gottsche = *Plagiochilasciophila*

Plagiochilabantamensisvar. γdenticulata Sande Lac. = *Plagiochilakurzii*

*Plagiochilabatjanensis* Schiffn. ex Herzog = *Plagiochilateysmannii*

*Plagiochilabelangeriana* Lindenb. = *Plagiochilaarbuscula*

Plagiochilablepharophoravar.major (Schiffn.) Bonner = *Plagiochilasandei*

*Plagiochilabrauniana* (Nees) Lindenb. ≡ *Chiastocaulonbraunianum*

*Plagiochilaceramica* Inoue = *Plagiochilapulvinata*

*Plagiochilacomata* (Nees) Lindenb. ≡ *Solenostomacomatum*

*Plagiochiladecidua* Inoue et Grolle = *Plagiochilasciophila*

*Plagiochiladendroides* (Nees) Lindenb. ≡ *Chiastocaulondendroides*

Plagiochilafrondescensvar.tenerrima (Nees) Schiffn. = *Plagiochilafrondescens*

*Plagiochilafrondescens* α *tenerrima* (Nees) Lindenb. = *Plagiochilafrondescens*

*Plagiochilafrondescens* γ *rigida* (Nees) Lindenb. = *Plagiochilafrondescens*

*Plagiochilagedeana* Schiffn. = *Plagiochilahampeana*

*Plagiochilanovae-guineae* Sande Lac. = *Plagiochilachauviniana*

*Plagiochilaopposita* (Reinw., Blume et Nees) Lindenb. ≡ *Chiastocaulonoppositum*

Plagiochilaopposita var. β falcata (Nees) Schiffn. ≡ Plagiochilaconjugatavar.falcata

Plagiochilaoppositavar. γfiliformis (Lindenb.) Schiffn. = *Chiastocaulonoppositum*

*Plagiochilaopposita* β *falcata* (Nees) Lindenb. ≡ Plagiochilaconjugatavar.falcata

*Plagiochilaopposita* γ *filiformis* Lindenb. *nom. inval*. = *Chiastocaulonoppositum*

*Plagiochilapseudaberrans* Inoue et Grolle = *Plagiochilagymnoclada*

*Plagiochilaseemannii* Mitt. = *Plagiochilasandei*

*Plagiochilasemialata* Sande Lac. = *Plagiochilateysmannii*

*Plagiochilasingularis* Schiffn. ≡ *Dinckleriasingularis*

Plagiochilatrapezoidea var. β tenera (Nees) Schiffn. = *Dinckleriasingularis*

Plagiochilatrapezoideavar. γmajor (Lindenb.) Schiffn. = *Plagiochilagigantealaxior*

*Plagiochilatrapezoidea* β *tenera* (Nees) Lindenb. = *Dinckleriasingularis*

*Plagiochilatrapezoidea* γ *major* Lindenb. *nom. illeg*. = *Plagiochilagigantealaxior*

*Plagiochilavariegata* Lindenb. = *Syzygiellasecurifolia*

*Plagiochilionbraunianum* (Nees) S.Hatt. ≡ *Chiastocaulonbraunianum*

*Plagiochilionfimbriatum* (Mitt.) Inoue ≡ *Chiastocaulonfimbriatum*

*Plagiochilionoppositum* (Reinw., Blume et Nees) S.Hatt. ≡ *Chiastocaulonoppositum*

*Plectocoleaariadne* (Taylor) Mitt. ≡ *Solenostomaariadne*

*Plectocoleacomata* (Nees) S.Hatt. ≡ *Solenostomacomatum*

Porellaacutifoliavar.subligulifera S.Hatt. = Porellaacutifoliavar.acutifolia

*Psilocladaunguligera* Schiffn. = Psilocladaclandestinasubsp.clandestina

*Ptychanthusjavanicus* Nees = Ptychanthusstriatusvar.striatus

*Ptychanthusmoluccensis* Sande Lac. = Ptychanthusstriatusvar.striatus

*Ptychanthussquarrosus* Mont. = *Ptychanthusstriatus*

*Ptychocoleusbrunneus* Steph. = Acrolejeuneapycnocladasubsp.pycnoclada

*Ptychocoleuscumingianus* (Mont.) Trevis. ≡ *Schiffneriolejeuneacumingiana*

*Ptychocoleusdensifolius* (Schiffn. ex P.Syd.) Steph. *nom. illeg.* = *Schiffneriolejeuneapulopenangensis*

*Ptychocoleushaskarlianus* (Gottsche) Steph. ≡ Schiffneriolejeuneatumidavar.haskarliana

*Ptychocoleuspulopenangensis* (Gottsche) Trevis. ≡ *Schiffneriolejeuneapulopenangensis*

*Ptychocoleuspycnocladus* (Taylor) Steph. ≡ *Acrolejeuneapycnoclada*

*Ptychocoleustener* Steph. = *Acrolejeuneafertilis*

*Pycnolejeuneabadia* Steph. = *Lepidolejeuneabidentula*

*Pycnolejeuneaceylanica* (Gottsche) Schiffn. ≡ *Cheilolejeuneaceylanica*

*Pycnolejeuneaceylanica* (Gottsche) Steph. *nom. inval*. ≡ *Cheilolejeuneaceylanica*

*Pycnolejeuneaconnivens* (Gottsche) Gottsche ex Schiffn. *nom. inval*. = *Pycnolejeuneaconnivens*

*Pycnolejeuneadecurvifolia* (Steph.) Steph. = *Lepidolejeuneabidentula*

*Pycnolejeuneademissa* Steph. ex Kachroo et R.M.Schust. *nom. inval.* = *Cheilolejeunealongidens*

*Pycnolejeuneafalsinervis* (Sande Lac.) Schiffn. ≡ *Cheilolejeuneafalsinervis*

*Pycnolejeuneagigantea* Steph. ≡ *Cheilolejeuneagigantea*

*Pycnolejeuneaincisa* (Gottsche) Schiffn. ≡ *Cheilolejeuneaincisa*

*Pycnolejeuneameyeniana* (Nees, Lindenb. et Gottsche) Steph. *nom. inval*. = *Cheilolejeuneatrapezia*

*Pycnolejeuneaschwaneckei* Steph. nom. inval. = *Pycnolejeuneaschwaneckei*

*Pycnolejeuneatrapezia* (Nees) Schiffn. ≡ *Cheilolejeuneatrapezia*

*Pycnolejeuneaventricosa* (Schiffn.) Schiffn. ex P.Syd. = *Cheilolejeuneaventricosa*

*Pycnolejeuneaventricosa* Schiffn. *nom. inval*. = *Cheilolejeuneaventricosa*

*Radulacampanigera* Mont. ≡ *Cladoradulacampanigera*

*Radulaceramensis* Steph. = *Radulajavanica*

Radulaformosavar.pycnolejeuneoides (Schiffn.) N.Kitag. = *Radulaformosa*

*Radulaovalifolia* Steph. = *Radulajavanica*

*Radulapycnolejeuneoides* Schiffn. = *Radulaformosa*

*Radulasubsimilis* Steph. = *Radulareflexa*

*Radulawallichiana* Lehm. = *Radulajavanica*

*Rhaphidolejeunealongicruris* (Steph.) Herzog ≡ *Drepanolejeunealongicruris*

*Riccardiapinguis* (L.) Gray ≡ *Aneurapinguis*

*Saccogynarigidula* (Nees) Schiffn. ≡ *Saccogynidiumrigidulum*

*Saccogynidiumjugatum* (Mitt.) Grolle = *Saccogynidiumrigidulum*

*Schiffneriolejeuneahaskarliana* (Gottsche) Gradst. ≡ Schiffneriolejeuneatumidavar.haskarliana

*Schismadivaricatum* Herzog = *Herbertussendtneri*

*Schismapilifera* (Schiffn.) Steph. = *Herbertuspilifer*

*Schismapiligerum* Steph. *nom. inval*. = *Herbertuspilifer*

*Schistochilaamboinensis* Steph. = *Schistochilabeccariana*

*Schistochilaformosana* Horik. = *Schistochilablumei*

*Schistochilagraeffeana* J.B.Jack et Steph. = *Schistochilaaligera*

*Schistochilainversa* Herzog = *Schistochilasciurea*

*Schistochilaphilippinensis* (Mont.) J.B.Jack et Steph. *nom. inval*. = *Schistochilaaligera*

*Schistochilaphilippinensis* (Mont.) Steph. = *Schistochilaaligera*

Schistochilaphilippinensisvar.transiens H.Buch *nom. inval*. = *Schistochilaaligera*

*Schistochilapurpurascens* Herzog = *Schistochilaacuminata*

*Schistochilarecurvata* H.Buch *nom. inval*. = *Schistochilaaligera*

Schistochilasciureaf.robustior Schiffn. = *Schistochilasciurea*

*Schistochilasteraliger* (Nees et Blume) H.A.Mill. ≡ *Schistochilaaligera*

*Schistochilastergraeffeanus* (J.B.Jack et Steph.) H.A.Mill. = *Schistochilaaligera*

*Schistochilasterphilippinensis* (Mont.) H.A.Mill. = *Schistochilaaligera*

*Sendtneradiclados* α *scorpioides* (Reinw., Blume et Nees) Nees = *Mastigophoradiclados*

*Sendtneradiclados* β *calcarata* (Reinw., Blume et Nees) Gottsche, Lindenb. et Nees *nom. inval*. = *Mastigophoradiclados*

*Sendtneravrieseana* Sande Lac. = *Mastigophoradiclados*

*Spruceanthusmarianus* (Gottsche) Mizut. ≡ *Cheilolejeuneamariana*

*Stenolejeuneadentata* (Steph.) Pócs ≡ *Lejeuneastenodentata*

*Strepsilejeuneacavistipula* Steph. ≡ *Pycnolejeuneacavistipula*

*Symphyogynopsisfilicum* (Nadeaud) Grolle = *Symphyogynopsisgottscheana*

*Syzygiellavariegata* (Lindenb.) Spruce = *Syzygiellasecurifolia*

*Taxilejeuneaalbescens* Steph. ≡ *Lejeuneaalbescens*

*Taxilejeuneakarstenii* Steph. = *Lejeunealumbricoides*

*Taxilejeunealaxiretis* (Steph.) Eifrig = *Lejeuneasordida*

*Taxilejeunealumbricoides* (Nees) Steph. ≡ *Lejeunealumbricoides*

*Taxilejeuneasordida* (Nees) Eifrig ≡ *Lejeuneasordida*

*Telaraneacuneifolia* (Steph.) J.J.Engel et G.L.Merr. ≡ *Neolepidoziacuneifolia*

*Telaraneakogiana* (Steph.) Grolle ≡ *Tricholepidoziakogiana*

*Telaraneaneesii* (Lindenb.) Fulford ≡ *Tricholepidozianeesii*

*Telaraneawallichiana* (Gottsche) R.M.Schust. ≡ *Neolepidoziawallichiana*

*Thysananthusrenilobus* (Gottsche) Schiffn. ≡ *Caudalejeuneareniloba*

*Thysananthusundulatus* (Gradst. et Grolle) Sukkharak *nom. inval*. = *Thysananthusfrauenfeldii*

*Trachylejeuneacavistipula* (Steph.) Steph. ≡ *Pycnolejeuneacavistipula*

*Trichocoleabreviseta* Steph. ≡ *Leiomitrabreviseta*

*Trichocoleastriolata* Steph. = *Trichocoleapluma*

Trichocoleatomentellavar.javanica (Reinw., Blume et Nees) Schiffn. = *Trichocoleapluma*

*Xenolejeuneaceylanica* (Gottsche) Tixier *nom. illeg*. ≡ *Cheilolejeuneaceylanica*

*Zoopsisrigida* Pearson = *Zoopsissetulosa*

## ﻿Additional information

### Author ORCIDs

Ainun Nadhifah https://orcid.org/0000-0003-0575-4306

Ida Haerida https://orcid.org/0000-0001-5769-9383

Fandri Sofiana Fastanti https://orcid.org/0000-0002-0925-7270

Lars Söderström https://orcid.org/0000-0002-9315-4978

Matt von Konrat https://orcid.org/0000-0001-9579-5325

### Data availability

All of the data that support the findings of this study are available in the main text.
